# Complexes of N‐Confused Porphyrin Derivatives as *Ortho*‐Metallating Ligands. Synthesis, Structure, Redox Properties, and Chirality

**DOI:** 10.1002/advs.202306696

**Published:** 2023-11-21

**Authors:** Sebastian Koniarz, Kinga Szydełko, Michał J. Białek, Karolina Hurej, Piotr J. Chmielewski

**Affiliations:** ^1^ Department of Chemistry University of Wrocław 14 F. Joliot‐Curie Wrocław 50383 Poland

**Keywords:** catalysis, chirality, coordination chemistry, organometallic complexes, porphyrinoids, transition metals

## Abstract

A family of transition metal complexes of meso‐aryl‐2‐aza‐21‐carbaporphyrin (N‐confused porphyrin, **NCP**) derivatives acting as *ortho*‐metallating ligands for ruthenium(II), rhodium(III), and iridium(III) is synthesized and characterized by XRD, spectroscopic, and electrochemical methods. The chirality of these systems is shown by the separation of the enantiomers and analyzed by circular dichroism and DFT. A preliminary catalytic study indicates the activity of the iridium(III) *ortho*‐metallated complexes in the N‐heterocyclization of primary amines with diols.

## Graphical abstract

N‐confused porphyrin and its 21‐substituted nickel(II) and ruthenium(II) complexes are used as chelating ligands for a series of late transition metals by exploiting external nitrogen of the confused pyrrole and an *ortho*‐carbon of the adjacent meso‐aryl. A new chirality center appears on the externally coordinated metal. Two higher oxidation states are available for the *ortho*‐metallated systems.

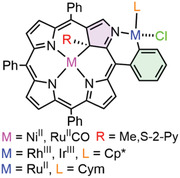

## 1 Introduction

**Figure 1:**
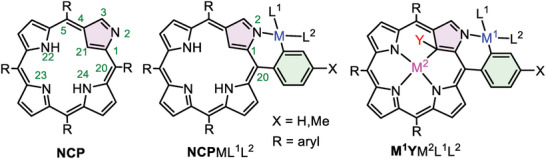
Schematic structures of NCP and its *ortho*‐metallated complexes.

The coordination chemistry of 2‐aza‐21‐carbaporphyrin, a.k.a. N‐confused porphyrin (**NCP**, **Figure** **1**) started simultaneously with the discovery of this porphyrinoid.^[^ ^1^, ^2^ ^]^ For almost three decades this macrocycle has attracted the attention of the researchers involved in the coordination chemistry of macrocycles due to its unusual NNNC coordination core, several distinct coordination modes that can be adopted by this porphyrinoid, as well as stabilization of the uncommon oxidation states.^[^ ^3^, ^4^, ^5^, ^6^, ^7^, ^8^, ^9^, ^10^, ^11^, ^12^, ^13^, ^14^, ^15^, ^16^, ^17^, ^18^ ^]^ The unique feature of **NCP** is the presence of a built‐in extra‐annular nitrogen donor that can be treated as an additional ligation site, naturally enriching the coordination chemistry of this macrocycle when compared with its isomer, i.e., regular porphyrin or various core‐modified analogues, including carbaporphyrins.^[^ ^19^, ^20^ ^]^ Thus, for the group 12 metals, manganese(II), or iron(II) complexes, the external nitrogen  N2 is coordinated to the metal center bound within the core of the adjacent **NCP** subunit forming a bridgeless homodimer or homotrimer.^[^ ^8^, ^10^, ^21^, ^22^, ^23^ ^]^ In a quite complicated structure of mixed‐valence tetrarhodium bis(**NCP**) tetracarbonyl complex, two external nitrogens are coordinated to a bridging dicarbonylrhodium(0) unit.^[^ ^13^ ^]^ Meanwhile, in a monomeric dirhodium(I) system, one dicarbonylrhodium(I) center occupies two internal nitrogen sites and the external nitrogen is coordinated to the chlorodicarbonylrhodium(I) unit.^[^ ^7^ ^]^ Upon coordination of two dicarbonyliridium(I) moieties, the **NCP** ligand undergoes inversion which results in the ligation of all four nitrogens inside the distorted macrocycle.^[^ ^12^ ^]^ Owing to the close location of the meso‐aryl at C20, the metal binding N2 can be a part of a six‐membered metallacycle involving C1, C20, C*_ipso_*, and C*_ortho_* (Figure 1). Such an *ortho*‐metallation has been relatively rarely observed and structurally characterized for **NCP** derivatives. In palladium(II) and platinum(II) dimers, the **NCP** subunits are bridged by the metal ions,^[^ ^24^, ^25^, ^26^ ^]^ while doubly‐*ortho*‐metallation of Pt^II^ or Pt^IV^ has been found to occur in [Pt(3,3′‐(**NCP**)_2_)] or [Pt{3,3′‐(**NCP**)}L^1^L^2^] comprising two directly linked **NCP** subunits.^[^ ^27^, ^28^ ^]^ In some of these complexes, the macrocyclic core is not involved in coordination, and in all of them, the confused pyrrole is tipped from the mean plane of the regular pyrroles which may be a prerequisite for the *ortho*‐metallation.

In this paper, we report the synthesis and characterization of several late transition metal complexes comprising **NCP** or its derivatives with the *ortho*‐C20–N2 chelating motif. We focus on the structural features of the **NCP** complexes that can be useful for transferring chirality onto the exposed “external” metal center M^1^ or potential catalytic activity.

## 2 Results and Discussion

### 2.1 Syntheses and Characterizations

**Scheme 1:**
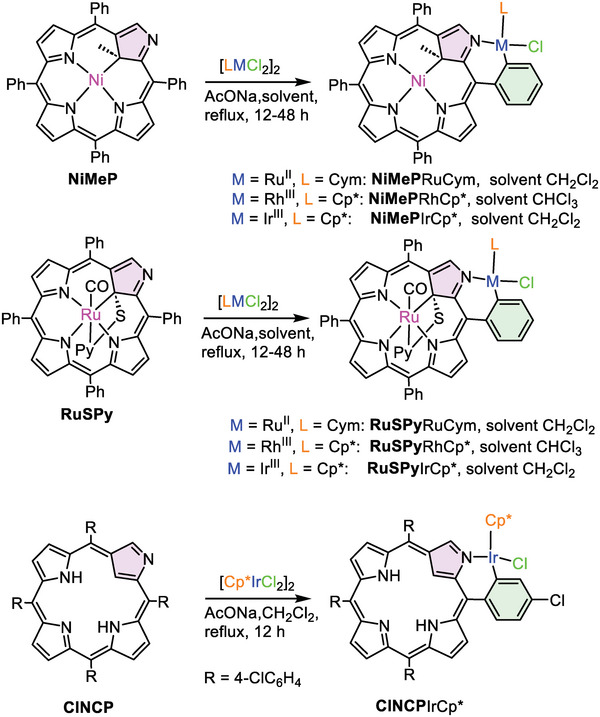
Syntheses of the *ortho*‐metallated **NCP** complexes.

As starting materials for our syntheses of bis‐metallic systems, we chose two previously reported compounds bearing a substituent at the C21 position coordinated to either nickel(II)^[^ ^29^, ^30^, ^31^, ^32^, ^33^ ^]^ or ruthenium(II).^[^ ^34^, ^35^ ^]^ The common features of these, otherwise different complexes are chirality, significant deviation from planarity of the porphyrin ring in the region of confused pyrrole, and unoccupied/non‐protonated N2. These systems were subjected to reaction with organometallic dimeric complexes of ruthenium(II), rhodium(III), or iridium(III) with two chlorides and either neutral η^6^‐*para‐*cymene (Cym) or η^5^‐pentamethylcyclopentadienyl anion (Cp*). Under mild conditions (reflux of DCM solution in the presence of sodium acetate as a proton scavenger) the reaction resulted in the substitution of one chloride by carbanionic *ortho‐*C20 and coordination of N2 atoms (**Scheme** **1**). The *ortho*‐metallation efficacy varied depending on the metal ion from 35–40% reaction yield for Rh^III^, 54–60% for Ru^II^, and up to 85–93% for Ir^III^. Slightly better results were obtained for **NiMeP** compared with those for **RuSPy**. We showed also that under the analogous condition, the reaction of [IrCl_2_Cp*]_2_ with **ClNCP** free base yielded *ortho*‐metallated derivative **ClNCP**IrCp* (Scheme 1) with a good outcome (83% yield).

**Figure 2:**
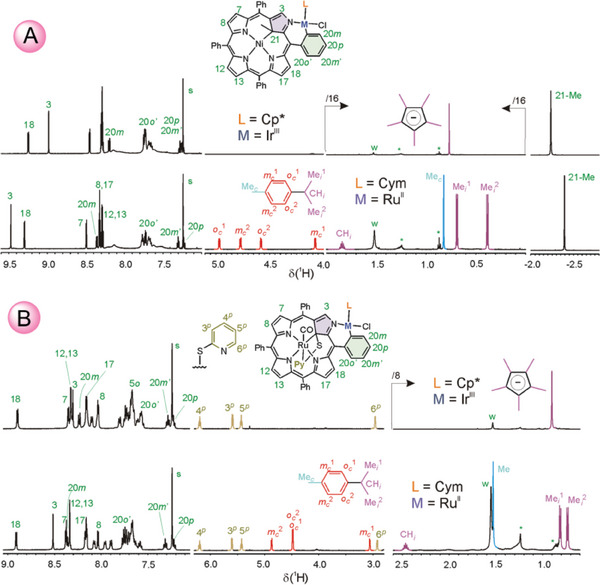
A) Selected regions of ^1^H NMR spectra (500 MHz, CDCl_3_, 300 K) of **NiMeP**IrCp* (top) and **NiMeP**RuCym (bottom) along with a partial signal assignment. B) Selected regions of ^1^H NMR spectra (500 MHz, CDCl_3_, 300 K) of **RuSPy**IrCp* (top) and **RuSPy**RuCym (bottom) along with a partial signal assignment. s, residual CHCl_3_ signal; w, dissolved water signal. The signal of impurities is marked with asterisks.

The new complexes were characterized by high‐resolution mass spectrometry, ^1^H and ^13^C NMR, including homo‐ and heteronuclear 2D correlation experiments, UV–vis–NIR spectroscopy, and single crystal XRD analyses. The ESI+ HRMS indicated the presence of the incoming metal ion as well as Cym or Cp* moieties in each case, though all cations were stripped of chloride. The ^1^H NMR spectra revealed the preservation of most of the spectral features typical of **NiMeP**, **RuSPy**, or **ClNCP** in the complexes formed and the appearance of new signals related to the presence of Cp* or Cym (**Figure** **2**). Thus, the spectra of the systems with Cp* comprise a very strong (15H) signal at about *δ* 0.8 ppm due to methyl protons, indicating fast rotation around the M–Cp* direction. On the other hand, the differentiation of aryl and isopropyl resonances of the RuCym moiety is in line with the slow motions of the Cym ligand at the ^1^H NMR time scale. Moreover, an upfield shift of all the Cym protons with respect to those observed for starting [RuCl_2_Cym]_2_, indicates the position of this moiety over the aromatic frame of the macrocyclic ligands. Significantly, this aromatic ring shielding effect is strongest for the cymene methyl Me*_c_* (Δ*δ* 0.7–1.4 ppm) suggesting the orientation of the ligand with the isopropyl group situated rather outside, while Me*_c_* is above the macrocycle. The number of meso‐aryl distinct signals in the spectra of **RuSPy**IrCp* and **RuSPy**RuCym recorded at room temperature, reflects diastereotopic inequivalence of the macrocyclic faces and slow, at the NMR timescale, rotation of these substituents around C_ipso_─C_meso_ bonds, resulting in differentiation of *ortho‐* and *meta‐*H resonances of phenyls at C5, C10, and C15. Conversely, these meso‐phenyl protons in **NiMeP**IrCp* and **NiMeP**RuCym give rise to severely broadened signals indicating intermediate rotation rate of the substituents and diastereotopicity of the complex faces. The only sharp meso‐aryl signals are those of phenyl at the C20 position due to the complete freezing of its rotation by *ortho*‐metallation. The chemical shifts and coupling patterns are similar in all these *ortho*‐metallated complexes comprising a doublet for 20‐*m* at about 8.3 ppm, a doublet for 20‐*o*′ at about 7.7 ppm, and two triplets for 20‐*m*′ and 20‐*p* at ≈7.3 and 7.2 ppm, respectively (Figure 2). For the *ortho*‐metallated rhodium(III) complexes, **NiMeP**RhCp* and **RuSPy**RhCp*, a close resemblance of

^1^H NMR spectral patterns to those of the respective Ir^III^ systems were observed. The major difference is an additional splitting of all multiplets of the 20‐phenyl protons due to ^1^H‐^103^Rh spin–spin coupling (*J_RhH_* = 1.3–1.5 Hz). Significantly, the *ortho*‐carbon of the C20 phenyl substituent, identified on the basis of ^1^H,^13^C HSQC and ^1^H,^13^C HMBC experiments (Figure S7B and S13B, Supporting Information), gives rise to a doublet at *δ_C_* 169.8 ppm with ^1^ *J_RhC_* = 31.1 Hz for **NiMeP**RhCp* and at *δ_C_* 169.9 ppm with ^1^ *J_RhC_* = 30.9 Hz for **RuSPy**RhCp* unequivocally proving coordination of the carbanion. The ^13^C–^103^Rh coupling could be observed also for cyclopentadiene carbon atoms in ^13^C NMR of both complexes giving rise to doublets at about *δ_C_* 96 ppm with ^1^ *J_RhC_* = 6.1 Hz. The ^1^H NMR characteristics of **Cl** **NCP**IrCp* differ from the nickel(II) and ruthenium(II) complexes with 21‐CH and 22,24‐NH resonances arising in the upfield region of the spectrum (*δ* −4.69 and broad signals at *δ* −1.05, −1.10 ppm, respectively) reflecting a free‐base character of the macrocyclic core. Interestingly, for all pentamethylcyclopentadienyl‐comprising systems, correlations of the coordinated *ortho*‐C with methyl protons of the η^5^‐Cp* ligand were observed in the ^1^H,^13^C HMBC maps (Figures S3B, S7B, S9B, S13B, and S15B, Supporting Information), regardless of the metal ion. Such correlations appeared despite the fact that the coupled nuclei were separated by four bonds. The correlations may be accounted for by the anionic character of the Cp* ligand resulting in a relatively high electron density available that enhanced ^1^H‐^13^C coupling. Significantly, for the neutral η^6^‐Cym ligand in **NiMeP**RuCym or **RuSPy**RuCym, there was no such a correlation observed, in spite of only three bonds separating aryl protons of this ligand and the Ru^II^‐coordinating *ortho*‐C at 20‐Ph.

**Figure 3:**
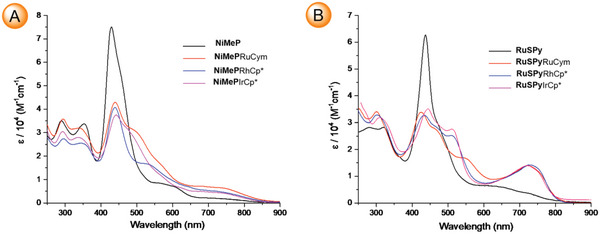
Optical spectra (DCM, 298 K): A) **NiMeP** and its *ortho*‐metallated derivatives and B) **RuSPy** and its *ortho*‐metallated derivatives.

The electronic spectrum of **ClNCP**IrCp* resembles that of the starting porphyrin **ClNCP** with ≈65 nm and 15 nm bathochromic shifts of the lowest‐energy Q band and the Soret band, respectively observed for the complex (Figure S1, Supporting Information). For the **NiMeP** and **RuSPy,** the spectral changes due to external coordination are much more profound, though similar to each other, regardless of the *ortho*‐metallated cation (**Figure** **3**). These alterations involve a decrease in the relative intensity of the spectra in the Soret region near 430 nm and an increase of the absorbance in the Q band region, i.e., above 500 nm.

### 2.2 Crystal Structures

**Figure 4:**
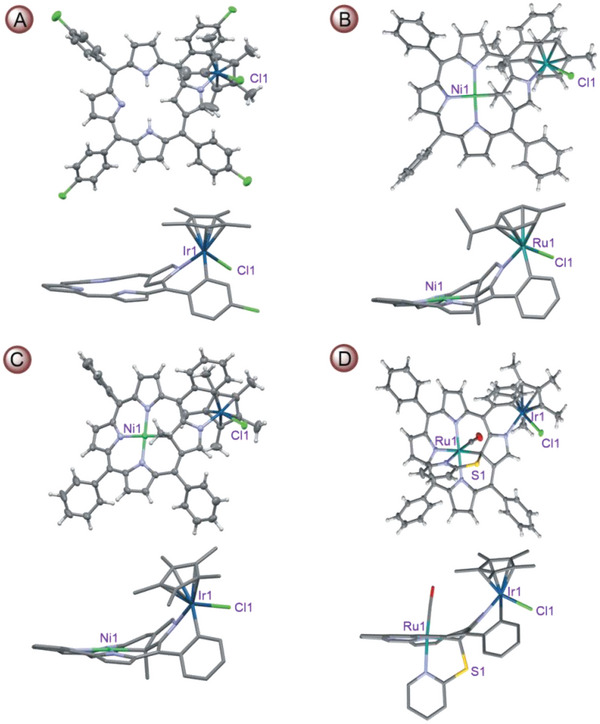
Perspective views (50% displacement ellipsoid plots and stick diagrams) of molecular structures of A) **ClNCP**IrCp*, B) **NiMeP**RuCym, C) **NiMeP**IrCp*, and D) **RuSPy**IrCp*. All solvent molecules are omitted. In the stick representations of the side views, all hydrogens and all but ortho‐metallated aryl substituents are removed for clarity.

Solid state structures of the selected *ortho*‐metallated systems were elucidated using the single crystal X‐ray diffraction analyses (**Figure** **4** Figures S39–S44, Supporting Information). The structures determined based on diffraction data clearly showed the coordination mode of iridium(III), ruthenium(II), and rhodium(III) ions to **ClNCP** or metalloligands **NiMeP** and **RuSPy**. Metalloligands bind iridium(III), ruthenium(II), and rhodium(III) ions through the N2 donor atom and the *ortho*‐carbon of the aryl ring from the C20 meso‐position. The coordination sphere of metal ions located at the periphery of the macrocycle is supplemented by chloride and pentamethylcyclopentadienyl ligands (in **ClNCP**IrCp*, **NiMeP**IrCp^*^, **RuSPy**IrCp*, and **RuSPy**RhCp*) or chloride and *p‐*cymene ligands (in **NiMeP**RuCym). Coordinating metal ions at the edges of metalloligands retain a structural motif referred to as a “piano stool” or a half‐sandwich complex.^[^ ^36^ ^]^ Data on bond lengths around iridium(III), ruthenium(II), and rhodium(III) ions are summarized in **Table** **1** along with selected bond lengths for the dimeric metal sources. The average M–L distances (where L = Cp* or Cym) are significantly longer than in starting half‐sandwich organometallic chlorides, while M–Cl bond lengths are close to those observed for terminally bound chlorides in the respective dimeric precursors or only slightly longer.^[^ ^37^, ^38^, ^39^ ^]^ Interestingly, **RuSPy**IrCp* and **RuSPy**RhCp* are isostructural when crystallized from benzene/hexane, both forming tris(benzene) solvates.

**Table 1:** Bond lengths *d* for the externally chelated metal ions.

Compound	*d* M–N [Å]	*d* M–C [Å]	*d* M–Cl [Å]	*d* M–(*η^n^─*L)^a)^ [Å]
**ClNCP**IrCp*	2.066(5)	2.051(6)	2.406(2)	2.187(6)
**NiMeP**IrCp*	2.065(2)	2.041(3)	2.407(1)	2.193(2)
**NiMeP**RuCym	2.069(3)	2.047(3)	2.408(1)	2.215(3)
**RuSPy**IrCp*	2.060(4)	2.083(5)	2.420(1)	2.198(3)
**RuSPy**RhCp*	2.072(4)	2.030(4)	2.402(1)	2.201(4)
[RuCl_2_Cym]_2_ ^b)^	–	–	2.444(4)^#^ 2.392(4)^&^	2.158
[RhCl_2_Cp*]_2_ ^c)^	–	–	2.458(9)^#^ 2.397(1)^&^	2.126
[IrCl_2_Cp*]_2_ ^d)^	–	–	2.453(5)^#^ 2.387(4)^&^	2.132

^a)^ Mean values of the M–C distances for *η* ^5^‐Cp*–M or *η* ^6^‐Cym–Ru;

^b)^ Data from ref. [39];

^c)^ Data from ref. [38];

^d)^ Data from ref. [37];

^#^ Bridging chloride;

^&^ Terminal chloride.

**Figure 5:**
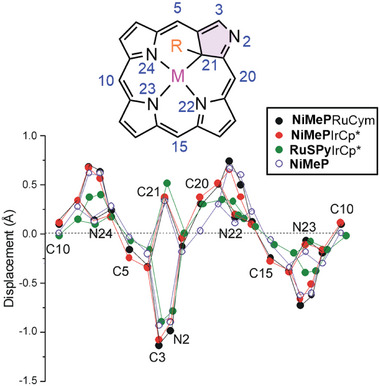
Atom displacements from the porphyrin mean plane calculated from SCXRD data of **NiMeP**RuCym (black dots), **NiMeP**IrCp* (red dots), **RuSPy**IrCp* (green dots), and **NiMeP** (violet circles).

The steric hindrance introduced by Cp* or Cym ligands forces them to be specifically positioned relative to methyl or mercaptopyridyl substituents at the C21 atom of metalloligand. As a consequence, they are located on opposite sides of the macrocyclic plane, and, in the case of compounds **RuSPy**IrCp* and **RuSPy**RhCp*, on the same side as the CO ligand. The specific setting of the *p*‐cymene relative to the macrocyclic system was established for **NiMeP**RuCym with the isopropyl group above the metalloligand plane. Such an orientation of the Cym ligand is somewhat unexpected considering solution ^1^H NMR results suggesting methyl rather than the isopropyl group directed toward the macrocycle interior (vide supra). It may be accounted for by packing forces for which the ligand orientation in the crystal with a smaller substituent situated beyond the perimeter of the macrocycle is more favorable. The deviation of the porphyrin ring from planarity due to sp^3^ hybridization of C21, characteristic for the starting metalloligand **NiMeP** and **RuSPy**, is retained upon coordinating the metal ion to the periphery of the macrocycle. However, the external *ortho*‐metallation does not significantly change the bond distance between the metal ion [nickel(II) or ruthenium(II)] located in the cavity of the macrocycle and the C21 carbon atom. The Ni─C21 bond length is 2.004(4) Å^[^ ^29^ ^]^ in **NiMeP**, while in **NiMeP**IrCp* and **NiMeP**RuCym it is 2.022(1) Å and 2.015(2) Å, respectively. For **RuSPy**‐containing systems, this bond length alteration is even less pronounced: from 2.118(3) Å in the metalloligand to 2.110(2) and 2.116(1) Å in **RuSPy**IrCp* and **RuSPy**RhCp*, respectively. Analyses of the out‐of‐plane porphyrin ring distortions were carried out using the *PorphStruct* tool^[^ ^40^ ^]^ which was based on the normal‐coordinate structure decomposition (NSD) approach^[^ ^41^, ^42^ ^]^ (**Table** **2** and **Figure** **5**). The comparative NSD analyses applied for starting systems, i.e., **ClNCP**, **NiMeP**, and **RuSPy**, indicated a moderate effect of the *ortho*‐metallation on the total out‐of‐plane distortion of the porphyrin (*D_oop_*). In fact, in some instances (**Cl** **NCP**IrCp*, **RuSPy**IrCp*, **RuSPy**RhCp*, Table 2, entries 2, 7, and 8, respectively) the chelation at the **NCP** perimeter led to a less pronounced displacement than that observed in the starting ligand (**NCP**, entry 1; **RuSPy**, entry 6). The most significant deviation increase due to *ortho*‐metallation was observed for the **NiMeP** metalloligand (Table 2, entry 3) upon chelation of RuCymCl moiety (entry 5). All systems indicated a significant saddling distortion which, again, increased only in **NiMeP**RuCym. The increasing doming, ruffling, and waving distortions were observed for almost all systems upon external chelation, although these components gave rather minor contributions to *D_oop_* which is dominated by the saddling. Displacement of metal ions from the mean plane of the porphyrin in a metalloligand (*d_M‐mpln_*) slightly increased after an external metal ion was introduced, though an opposite effect was observed for the displacement from an MCNNN mean plane (*d_M‐ccpln_*). A common structural feature for the complexes under study as well as for **NCP** free base, is a pronounced deviation of the confused pyrrole plane from that of the porphyrin ring. Such a deviation can be parametrized by a dihedral angle (*DH*, Table 2) between the confused pyrrole mean plane and the mean plane defined by all non‐hydrogen atoms of the macrocycle, except N2, C3, and C21. Apparently, a significant increase of this angle upon external chelation was observed only for the **NiMeP‐**containing complexes (Table 2, entries 4 and 5). The individual atom displacements from the mean plane are typical for the saddle‐distorted porphyrins with alternate directions of the pyrrole deviation (Figure 5). Significantly, in the bimetallic systems **NiMeP**IrCp* and **NiMeP**RuCym, the displacement of the meso‐carbons is significantly more pronounced than in the starting **NiMeP**, where those atoms are located almost in the mean plane. It is particularly evident for C20 and is related to the coordination of N2 and the aryl at C20. The external chelation slightly increases the displacement of N2 and C3 in these two systems with respect to the metalloligand, in line with the increasing *DH*. Generally, the atoms are more displaced in **NiMeP** and its *ortho*‐metallated derivatives than in **RuSPy** and its complexes.

**Table 2:** Analyses of the out‐of‐plane displacements for the macrocyclic ring of **NCP** calculated by means of PorphyStruct.^[^ ^40^ ^]^ on the basis of SCXRD structures.

Entry	Compound	*doming* [Å]	*saddling* [Å]	*ruffling* [Å]	*wavingX* [Å]	*wavingY* [Å]	*propellering* [Å]	*d_M‐mpln_* ^a)^ [Å]	*d_M‐ccpln_* ^b)^ [Å]	*D_oop_* ^c)^ [Å]	*DH* ^d)^ [deg]
1	**NCP** ^e)^	0.196	1.360	−0.070	0.259	−0.055	0.040	–	–	1.465	27.0
2	**ClNCP**IrCp*	0.341	−1.165	0.047	−0.108	−0.354	−0.026	–	–	1.325	28.0
3	**NiMeP** ^f)^	0.271	−2.069	−0.092	−0.011	−0.353	0.003	0.022	−0.077	2.181	38.2
4	**NiMeP**IrCp*	0.428	2.020	0.729	−0.515	−0.180	0.027	0.088	0.061	2.290	43.7
5	**NiMeP**RuCym	−0.437	−2.233	−0.580	0.499	0.090	−0.028	−0.083	0.067	2.445	45.5
6	**RuSPy** ^g)^	0.415	−1.804	0.282	0.058	0.326	−0.003	0.046	−0.108	1.990	40.2
7	**RuSPy**IrCp*	0.559	1.343	0.312	−0.607	−0.246	0.018	0.086	−0.098	1.689	41.3
8	**RuSPy**RhCp*	0.554	1.315	0.291	−0.590	−0.258	0.018	0.088	−0.096	1.657	41.2
11	**NCP**PtPPh_3_ ^h)^	−0.278	1.290	−0.320	0.107	0.437	0.008	–	–	1.501	31.5

^a)^ The metal ion (Ni^II^ or Ru^II^) displacement out of the porphyrin mean plane;

^b)^ The metal ion (Ni^II^ or Ru^II^) displacement out of the mean plane of the coordination core (*M*CNNN);

^c)^ total out‐of‐plane distortion;

^d)^ dihedral angle between the mean plane defined by all non‐hydrogen atoms of the macrocyclic ring except C21, N2, and C3 but including core‐coordinated metal, and the mean plane of the confused pyrrole;

^e)^ X‐ray data taken from ref. [2];

^f)^ X‐ray data taken from ref. [29];

^g)^ X‐ray data taken from ref. [34];

^h)^ X‐ray data taken from ref. [25];

All *ortho*‐metallated complexes are chiral, as are their precursors in the solid state. However, these systems crystallized in centrosymmetric space groups as racemates and in Figure 4 only one enantiomer for each of the complexes has been shown.

### 2.3 Chirality

**Figure 6:**
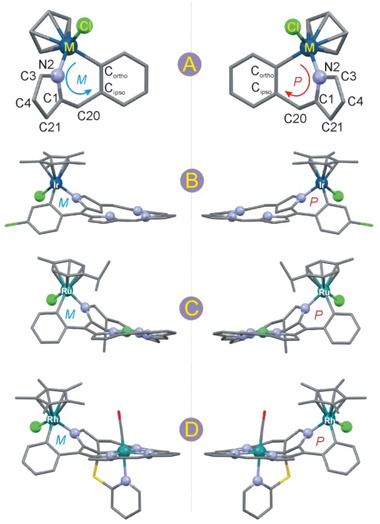
Definition of absolute configuration at metalacyclic part of the systems A) and enantiomers along with chirality definitions for the selected *ortho*‐metallated **NCP** complexes, B) **ClNCP**IrCp*, C) **NiMeP**RuCym, and D) **RuSPy**RhCp*.

Our attempt to separate enantiomers of the externally metallated **NCP** derivatives involved HPLC methods with a chiral stationary phase. The enantiomers of several of these systems are presented in **Figure** **6** as they appear in the solid‐state structures, along with definitions of their absolute configurations. For these definitions, we took an external metallacycle which is common to all these *ortho*‐metallated systems, i.e., M─N2─C1─C20─C*_ipso_* ─C*_ortho_* as a chirality plane.

**Scheme 2:**
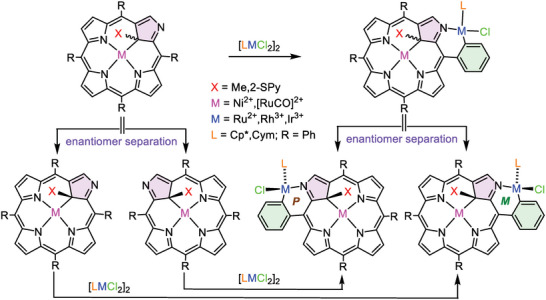
Two approaches applicable for the separation of enantiomers of the *ortho*‐metallated complexes.

**Figure 7:**
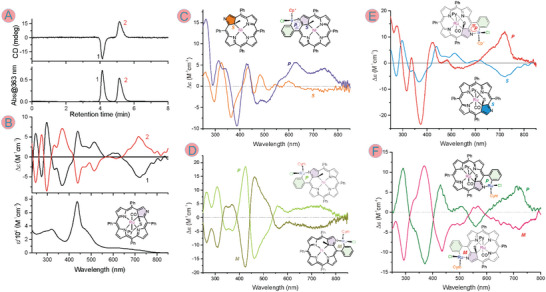
A) HPLC profiles for **RuSPy** on chiral stationary phase column (Chirex 3010, 1% MeOH in CH_2_Cl_2_, 2 mL min^−1^); top, CD and bottom, absorbance detection at 363 nm. B) CD (top) and absorbance (bottom) spectra of the HPLC‐separated fractions of **RuSPy**. C) superimposed CD spectra (CH_2_Cl_2_, 298 K) of *S‐* **NiMeP** (orange trace) and *P*‐**NiMeP**IrCP* (purple trace) obtained by metalation of the former with [IrCl_2_Cp*]_2_. D) CD spectra of enantiomers of **NiMeP**RuCym separated by the chiral stationary phase HPLC. E) superimposed CD spectra (CH_2_Cl_2_, 298 K) of *S‐* **RuSPy** (blue trace) and *P*‐**RuSPy**IrCP* (red trace) obtained by metallation of the former with [IrCl_2_Cp*]_2_. F) CD spectra of enantiomers of **RuSPy**RuCym separated by the chiral stationary phase HPLC. The absolute configurations were assigned to the enantiomers on the basis of TD‐DFT simulations of the CD spectra (Figures S23–S26, Supporting Information).

Although metalloligands **NiMeP** and **RuSPy** are intrinsically chiral due to the presence of a chirality center at the coordinated C21,^[^ ^43^ ^]^ the *ortho*‐metallation introduces its own chirality related to a differentiation of the porphyrin faces. Apparently, these two chirality sources are not independent, that is, upon external chelation of the racemic mixture of **NiMeP** or **RuSPy** only a pair of enantiomers is formed and no other NMR‐distinguishable stereoisomers can be observed. Hence, the external metallation is stereoselective although two diastereomers can be potentially formed, differing in orientation of chloride and the organometallic ligands (Cp* or Cym) at the externally chelated metal with respect to the macrocycle. The crystal structures reveal roughly the same orientation of the chloride ligand at the external metal center and the substituent at C21. Thus, the chirality of the bimetallic monomers in the solid state and solution is predefined by the starting metalloligands^[^ ^43^, ^44^ ^]^ with absolute configurations *S* or *R* at C21 in the metalloligands invariantly giving rise to the configurations *P* or *M*, respectively in the *ortho*‐metallated complexes. The separation of the bimetallic enantiomers by the HPLC method was expected to be effective through two approaches (**Scheme** **2**). The first method involved a separation of enantiomers prior to the external chelation (**Figure** **7**A,B,C,E), while in the second approach, separation proceeded *ortho*‐metallation (Figure 7D,F; Figures S29 and S30, Supporting Information). The first method allows high enantiopurity of the bimetallic systems and, in principle, can be applied for many other metal ions resulting in chirality transfer from the metalloligand toward the external metal center which may be a site of catalytic reaction. Importantly, the absolute configurations of such complexes can be deduced directly from the absolute configuration of the metalloligand. The second method is more useful for the systems for which the external chelation is less effective, such as **NiMeP**RhCp* or **RuSPy**RhCp*.

The asymmetry of these configurationally stable systems arises from the presence of the chiral center at the C21 atom. Conversely, the **NCP** free base is chiral in the solid state owing to its non‐planarity but in solution, the molecule is configurationally unstable and no enantiomer separation is possible. This is due to a flipping of the confused pyrrole allowing fast interconversion of the enantiomers, unlike in several 21‐substituted **NCP** derivatives.^[^ ^45^, ^46^ ^]^ Thus, chelation of metal ion by N2 and the *ortho* carbon atom of the adjacent meso‐aryl such as in **ClNCP**IrCp* or **NCP**PtPPh_3_^[^ ^25^ ^]^ as well as double chelation in directly bound 3,3′‐(**NCP**)_2_Pt^[^ ^28^ ^]^ is sufficient to stabilize the configuration. The external *ortho*‐metallation provides a lock preventing fast interconversion of enantiomers and participates in the differentiation of the macrocycle faces. Thus, for **ClNCP**IrCp*, we were able to separate enantiomers and record their circular dichroic spectra (Figures S27 and S28, Supporting Information) indicating the chirality of this system and its configurational stability. The absolute configurations of the separated enantiomers were assigned based on TD–DFT simulations of the CD spectra (Figures S23–S27, Tables S7–S12, Supporting Information).

### 2.4 Redox Properties

**Figure 8:**
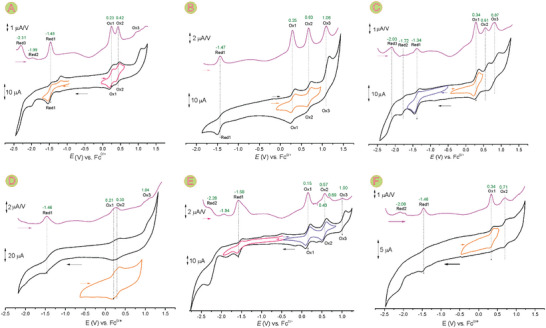
Cyclic (CV, black traces) and differential pulse (DP, purple traces) voltammograms of A) **NiMeP**IrCp*, B) **RuSPy**IrCp*, C) **ClNCP**IrCp*, D) **NiMe**RuCym, E) **RuSPy**RuCym, and F) **RuSPy**RhCp*. The experiments were carried out in a dichloromethane solution of [Bu_4_N]PF_6_ (0.1 m) using a glassy carbon working electrode, a platinum wire as an auxiliary electrode, and Ag/AgCl as a pseudoreference electrode. The green numbers associated with DP peaks are electrode potentials in volts. The partial CV scans are given to indicate the reversibility of some of the processes.

The electrochemical properties of the *ortho*‐metallated complexes were studied by means of cyclic and differential pulse voltammetry (**Figure** **8**; Figure S35, Supporting Information). The electrode potentials were collected in **Table** **3**. For all but one complex system the first oxidations were reversible, and for a majority, the second oxidations appeared to be reversible as well. Conversely, for almost all complexes there was no reversible reduction. Oxidation potentials were relatively low and the external coordination did not significantly affect the first oxidation potentials compared to the metalloligands **NiMeP** and **RuSPy** (Table 3, entries 8, 9) or ligand **ClNCP** (Table 3, entry 10). It was not the case for **RuSPy**IrCp* and **RuSPy**RuCym (Table 3, entries 5 and 7) for which 90 and 190 mV cathodic shifts were observed, respectively.

**Table 3:** Electrode potentials of the orthometallated complexes and metalloligands.

Entry	Compound	*E*_Red3_ [V]	*E*_Red2_ [V]	*E*_Red1_ [V]	*E*_Ox1_ [V]	*E*_Ox2_ [V]	*E*_Ox3_ [V]	Δ*E* ^a)^ [V]	oHLG^e)^ [eV]	cHLG^f)^ [eV]
1	**ClNCP**IrCp*	−2.03^b)^	−1.72^b)^	−1.34	0.34	0.61^b)^	0.87^b)^	1.68	1.53	2.02
2	**NiMeP**IrCp*	−2.31^b)^	−1.99^b)^	−1.48^b)^	0.23	0.42	0.92^b)^	1.71	1.60	2.29
3	**NiMeP**RhCp*	−2.18^b)^	−1.96^b)^	−1.46	0.29^b)^	0.52	0.91^b)^	1.75	1.56	N.A.
4	**NiMeP**RuCym	–	–	−1.46^b)^	0.21	0.30	1.04^b)^	1.67	1.57	2.27
5	**RuSPy**IrCp*	–	–	−1.47^b)^	0.25	0.63	1.06^b)^	1.72	1.61	2.15
6	**RuSPy**RhCp*	–	−2.08^b)^	−1.46^b)^	0.34	0.71^b)^	–	1.80	1.63	2.19
7	**RuSPy**RuCym	−2.28^b)^	−1.94^b)^	−1.58	0.15	0.57	1.00^b)^	1.73	1.60	N.A.
8	**NiMeP** ^c)^	–	–	−1.37	0.22	0.50^b)^	–	1.59	1.46	N.A.
9	**RuSPy** ^d)^	–	–	−1.39^b)^	0.34	0.79	–	1.73	1.65	2.39
10	**ClNCP**	–	−2.09^b)^	−1.42^b)^	0.36^b)^	0.63^b)^	0.84^b)^	1.78	1.61	N.A.

^a)^ Electrochemical HOMO–LUMO gaps Δ*E* = *E*_Ox1_ ─ *E*_Red1_;

^b)^ Irreversible process;

^c)^ Data from ref. [33];

^d)^ Data from ref. [34];

^e)^ Optical HOMO–LUMO energy gaps derived from onsets of the lowest‐energy bands in the electronic spectra;

^f)^ HOMO–LUMO energy gaps derived from DFT calculations.

**Figure 9:**
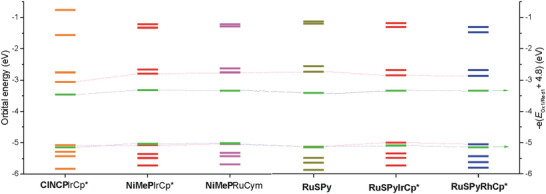
DFT‐calculated frontier orbitals energies of specified systems with the experimentally estimated energies of the HOMO and LUMO derived from the electrochemical data (green sticks).

The redox properties of the *ortho*‐metallated species and their precursors can be analyzed theoretically through comparison of the frontier orbitals energies (**Figure** **9**). Apparently, the calculated HOMO energies are very similar for all these systems with only a small rise of potential on going from **RuSPy** to the *ortho*‐metallated derivatives which can also be noticed experimentally as an increase of oxidation potentials. The DFT‐calculated HOMO energies are in good agreement with those estimated based on the first oxidation potentials [calculated as −e(*E*_Ox1_ + 4.8 V)], but LUMO energies are systematically higher than those derived from the first reduction potentials *E*_Red1_ [calculated as −e(*E*_Red1_ + 4.8 V)]. Consequently, the electrochemical HOMO–LUMO gaps [calculated as e(*E*_Ox1_ − *E*_Red1_)] are considerably smaller (by 0.4–0.6 eV) in comparison with those based on DFT calculations (cHLG in Table 3). On the other hand, the optical HOMO–LUMO energy gaps (oHLG in Table 3.), obtained from the experimental UV–vis spectra are even lower (by 0.1–0.2 eV) than the values derived from the electrochemical potentials.

**Figure 10:**
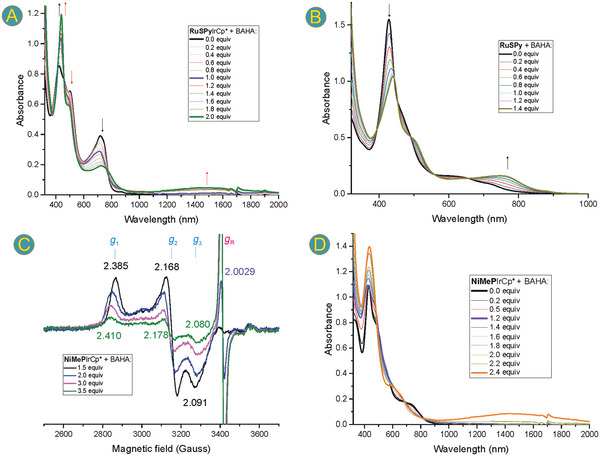
Spectrophotometric titration of dichloromethane solutions of A) **RuSPy**IrCp*, B) **RuSPy**, and D) **NiMeP**IrCp* with tris(4‐bromophenyl)ammoniumyl hexachloroantimonate (BAHA) and selected ESR spectra recorded in frozen dichloromethane solutions (120 K) upon addition of specified amounts of BAHA (C). The black arrows in (A) and (B) indicate the direction of the absorbance changes before, while the red arrows—after the addition of 1 equiv of BAHA.

Spectrophotometric titration of both **NiMeP** and **RuSPy** *ortho*‐metallated derivatives with tris(4‐bromophenyl)ammoniumyl hexachloroantimonate (BAHA, Magic blue), a one‐electron oxidant of reduction potential 0.70 V^[^ ^47^ ^]^ allowed monitoring of the spectral changes upon oxidation (**Figure** **10**). According to our electrochemical data, this oxidant was sufficiently strong for the first and the second oxidation to be achieved for all systems, except **RuSPy** for which *E*_Ox2_ is too high (Table 3, entry 9). The observed changes in the spectral region between 400 and 800 nm upon the addition of one equivalent of BAHA, suggested metal‐centered oxidation in all the bimetallic systems (Figure 10A,D; Figure S31–S34, Supporting Information). For the **RuSPy**MCp* complexes (M = Ir^III^, Rh^III^), the first oxidation resulted in an increase of the Soret‐like band intensity with a small (Δ*λ* = 15 nm) bathochromic shift and a decrease of the Q‐type band (Figure 10A; Figure S34, Supporting Information). Such changes suggest that the conjugation of the π‐electrons of the aromatic macrocycle remains intact after one electron is removed. Further addition of BAHA resulted in a gradual increase of the absorbance in the NIR region at ≈1500 nm which is indicative of a radical species, thus suggesting a ligand‐centered second oxidation process. These spectral changes are in sharp contrast to those observed for the **RuSPy** metalloligand for which a pronounced decrease of the Soret‐like and an increase of the Q‐like bands were observed and no further spectral alteration occurred after passing 1 equiv of BAHA (Figure 10B). Such a pattern of spectral changes may suggest a porphyrin‐centered oxidation to the cation metalloradical rather than Ru‐centered oxidation. On the other hand, titration of **ClNCP**IrCp* with BAHA indicated an initial decrease of the Soret‐like band at 448 nm and its bathochromic shift up to 463 nm upon the addition of 1 equiv of the oxidant. The changes were followed by an increase in intensity of this band with a further redshift to 469 nm which was accompanied by the absorbance increase at 816 nm and the formation of the broadband at 1500 nm when approaching 2 equiv of BAHA (Figure S31, Supporting Information). Thus, apparently despite oxidation of the “empty” macrocycle in **ClNCP**IrCp* the aromaticity of the system is retained and typical spectral features of the porphyrinoids, i.e., the strong Soret‐like band and weaker Q‐type bands are observed even for the two‐electron oxidized species. The first oxidations of the systems comprising **NiMeP** are nickel‐centered. The highly anisotropic orthorhombic frozen dichloromethane ESR spectra obtained by the BAHA addition (Figure 9C) closely resemble those of various 21‐alkylated nickel(III) **NCP** species.^[^ ^32^, ^33^, ^48^, ^49^ ^]^ For **NiMeP**IrCp*, the addition of more than 1.5 equiv of BAHA resulted in a gradual decrease of the nickel(III) signal intensity, moderate changes of the Zeeman tensor components, and in the appearance of a radical signal at *g* = 2.0029. Also, spectrophotometric titration with BAHA revealed fine changes in the Soret and Q regions up to 1.2 equiv, followed by a gradual increase of the band at 435 nm and the final appearance of the NIR band upon the addition of more than 2 equiv of the oxidant (Figure 9D).^[^ ^50^ ^]^ Interestingly, for the very similar complex, i.e., **NiMeP**RhCp*, the changes of the ESR spectra upon the addition of 1.5 or more equivalents of BAHA are different, involving slight alteration of *g*_2_ and *g*_3_ components, not the appearance of the radical signal (Figure S35, Supporting Information). Similarly, the addition of BAHA to the solution of **NiMeP**RuCym gave rise to an orthorhombic spectrum in frozen DCM (Figure S36, Supporting Information) but no strong radical signal was observed upon the addition of more than 2 equiv of BAHA. For reference purposes, we performed also the ESR‐monitored oxidation for the starting metalloligand **NiMeP** indicating a decrease of the Zeeman tensor anisotropy upon the addition of an excess of BAHA with changes of *g*_1_ from 2.382 to 2.285, *g*_2_ from 2.168 to 2.2019, and *g*_3_ from 2.086 to 2.111 for the spectra recorded in the presence of 1.2 and 3 equiv, respectively (Figure S37, Supporting Information). Again, no accompanying radical formation was observed. The differences among the systems comprising **NiMeP** in the second oxidation potentials and ESR behavior are surely related to the external coordination, although no clear tendencies can be derived from such a limited data set. It can be also rationally expected that for both groups of the *ortho*‐metallated complexes, oxidation of the metalloligand strongly affects electron density in the environment of the externally coordinated metal ion.

### 2.5 Catalysis

For preliminary studies of the catalytic function of the externally *ortho*‐metallated systems, we chose *N*‐heterocyclization reaction of benzylamine with 1,6‐hexanediol which has been shown to be effectively catalyzed by iridium(III) complex [IrCl_2_Cp*]_2_ in toluene under basic conditions (**Table** **4**).^[^ ^51^ ^]^ Small‐scale reactions (0.5 mmol) were carried out in the presence of all *ortho*‐metallated iridium(III) complexes described in this paper as well as for the original catalyst [IrCl_2_Cp*]_2_, **ClNCP** ligand, and both metalloligands, for the reference. The samples were prepared in the inert and dry atmosphere of a glove box to avoid any interference of oxygen and moisture with the reagents, and the reactions were carried out in sealed vials. The reaction results were analyzed qualitatively using GC/MS and quantitatively using the GC/FID technique, and for the selected systems, ^1^H NMR quantitative analysis was applied with 2,4,6‐collidine as the internal standard. As expected, only iridium(III)‐comprising systems appeared to be catalytically active in the heterocyclizaction reaction, while neither of the ligands supported the 7‐membered ring formation. The best catalyst, i.e., **ClNCP**IrCp* (Table 4, entry 6) gave rise to the yield exceeding that of the original catalyst [IrCl_2_Cp*]_2_ with higher glycol conversion and better chemoselectivity. Also **NiMeP**IrCp* seemed to be a more selective catalyst than [IrCl_2_Cp*]_2_ with a similar yield of the main reaction product (Table 4, entries 2 and 1). Surprisingly, no heterocyclization was observed for **RuSPy**IrCp* used as a catalyst, despite several attempts. It may be due to a less labile character of the Ir─Cl bond in this complex than in other systems. According to the proposed reaction mechanism,^[^ ^51^ ^]^ in the early stage of the catalytic process, coordination of the glycolic anion to the iridium center is required which implies chloride substitution (originally present in the *ortho*‐metallated systems). A significantly longer C─Ir bond in **RuSPy**IrCp* (2.083(5) Å) than those in **NiMeP**IrCp* (2.041(3) Å) or **ClNCP**IrCp* (2.051(6) Å) may be responsible for a weaker *trans* effect of the meso‐aryl carbanion coordinated to the iridium(III) center on the opposite side to the chloride, making its exchange less effective. Of course, some other structural features, such as the presence and type of the metal ion within the macrocyclic cavity, flexibility of the molecular skeleton and its deformations, etc., may be decisive for the catalytic activity of the iridium complexes. Although at the present stage, we cannot offer any more conclusive accounting for the observed differences, it is clear that distinctions in the reaction yields among the systems used in this study indicate an influence of the porphyrin‐chelating ligand on the catalytic activity of the externally coordinated metal center.

**Table 4:** Reaction conditions and yields for benzylamine (A) reacting with 1,6‐hexanediol (B) in the presence of various catalysts.

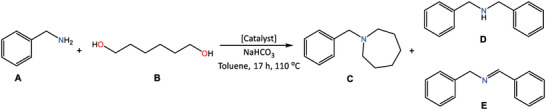							
Entry^a)^	Catalyst	%mol^b)^	µmol of Ir	Conversion of B [%]	Yield C [%]	Yield D [%]	Yield E [%]
1	[IrCl_2_Cp*]_2_	0.6	6	90	66^c)^	43	2
2	**NiMeP**IrCp*	1.0	5	96	67	18	<1
3	**NiMeP**	1.0	0	< 5	n.d.	n.d.	15^d)^
4	**RuSPy**	1.0	0	< 5	n.d.	n.d.	11^d)^
5	**RuSPy**IrCp*	1.0	5	< 5	n.d.	n.d.	18^d)^
6	**ClNCP**IrCp*	1.1	6	99	86	19	2
7	**ClNCP**	1.0	0	< 5	n.d.	n.d.	2^d)^

^a)^ Reactions were carried out for 0.5 mmol (59 mg) of 1,6‐hexanediol **B** and 0.75 mmol (80 mg) of benzylamine **A** in the presence of 1 mg of NaHCO_3_. The yields were estimated employing quantitative ^1^H NMR of the reaction mixtures with 2,4,6‐collidine as an internal reference unless stated otherwise;

^b)^ With respect to 1,6‐hexanediol;

^c)^ The reported yield was 74%^[^ ^51^ ^]^;

^d)^ Results from GC–MS/FID analysis only.

## 3 Conclusion

We have shown here that the *ortho*‐metallation of the **NCP** by representative late metals of the second and third rows of transition metals appears to be facilitated by a deviation of the confused pyrrole from the macrocycle mean plane. Such a deviation can be easily achieved when the macrocyclic crevice is empty, and thus, when the ligand is sufficiently flexible to conform the external donor set, i.e., the external N2 atom and a C*_ortho_* atom of the aryl at the meso‐C20 position to afford chelation. This strong out‐of‐plane deflection also allows the coordination of other ligands supplementing the coordination sphere of the metal. Importantly, in the already known *ortho*‐metallated **NCP** complexes, the metals (Pd^2+^, Pt^2+^) coordinated to the ligand exterior adopt invariantly a square‐planar geometry. In the present report, we show that a piano‐stool environment is also a suitable geometry for this type of complex, despite the presence of relatively voluminous ligands (Cym or Cp*). Although not as flexible as a free base, the C21‐substituted **NCP** derivatives coordinating in the macrocyclic interior to Ni^2+^ in the square planar or Ru^2+^ in the octahedral environments, comprise the confused pyrrole unit that is permanently deviated from the mean plane of the porphyrinoid. This arrangement makes complexes such as **NiMeP** or **RuSPy** effective metalloligands suitable for *ortho*‐metallation. The *ortho*‐metallated systems are chiral and can be obtained in a non‐racemic form by either separation of the final bimetallic complexes or by the external metalation of the separated enantiomers of metalloligands, as no racemization is possible upon N2–C*_ortho_* chelation. Favorably, the external chelation occurs stereoselectively with only one arrangement of the metal environment and the macrocycle. Thus, the chiral information can be transferred from the metalloligand to the externally situated metal center of the asymmetric environment. The redox properties of the bimetallic complexes are not profoundly altered in comparison to those of the appropriate metalloligands as has been shown here by electrochemical studies as well as by spectrophotometric and ESR‐monitored oxidation. Preliminary recognition of an influence of the *ortho*‐metallated ligand on the catalytic activity of the externally coordinated iridium(III) centers indicated that the heterocyclization reaction is totally absent for the system comprising **RuSPy** metalloligand (i.e., **RuSPy**IrCp*), despite the same donor set as in **NiMeP**IrCp* or **ClNCP**IrCp* that effectively catalyzed this reaction. The study on these and other analogous systems directed toward recognition of their catalytic potential and redox properties will be continued in our laboratory.

## 4 Experimental Section

### General Methods and Instrumentation

Commercial reagents were used without further purification. Solvents were freshly distilled from the appropriate drying agents or purified under nitrogen with the mBraun MBSPS‐800 before use. Column chromatography was performed by using silica gel 60 (200–300 mesh ASTM). The NMR spectra were recorded on a Bruker Avance III spectrometer, operating at 500 MHz for ^1^H and 125 MHz for ^13^C, or a Bruker Avance III spectrometer operating at 600 MHz for ^1^H and 150 MHz for ^13^C. TMS was used as an internal reference for ^1^H and ^13^C chemical shifts and CDCl_3_ was used as solvent. Standard pulse programs from the Bruker library were used for homo‐ and heteronuclear 2D experiments. ESR spectra (X‐band) were recorded on a Bruker ELEXSYS E500 spectrometer. Mass spectrometry measurements were conducted by using the electrospray ionization technique on a Bruker Daltonics microTOF‐Q or using the MALDI method on a Bruker ultrafleXtreme spectrometer. Absorption UV/Vis/NIR spectra were recorded by using a Varian Cary 60 and Jasco V‐770 spectrophotometers. Circular dichroic spectra were recorded by means of Jasco 1500 spectropolarimeter equipped with a flow cell attached to the Hitachi‐Merck LaChrom HPLC system allowing detection of the chiral fraction and CD spectra measurement in a stopped‐flow technique. Enantiomer resolutions were performed using either Chirex 3010 or Chirex 3014 column (25 × 0.46 cm). The product of the catalytic reactions was analyzed utilizing an Agilent 8890 gas chromatograph equipped with an Agilent 122–5532 column (30 m × 250 µm × 0.25 µm with DB‐5 ms stationary phase) and with mass spectrometer detector Agilent 5977B. Quantitative analyses were performed on the same chromatograph with Agilent 19091S‐433UI column and FID detector. Electrochemical measurements were performed by means of Autolab (Metrohm) potentiostat/galvanostat system for dichloromethane solutions with a glassy carbon, a platinum wire, and Ag/Ag^+^ as the working, auxiliary, and pseudoreference electrodes, respectively. Tetrabutylammonium hexafluorophosphate was used as a supporting electrolyte. The potentials were referenced with the ferrocene/ferrocenium couple used as an internal standard.

The X‐ray diffraction was measured using either XtaLAB Synergy R or Xcalibur, Onyx diffractometers using a copper source of radiation (λ = 1.54184 Å), and collected with CCD camera. The standard temperature of the measurement was 100 K. The structures were solved using direct methods with SHELXT^[^ ^52^ ^]^ and refined by the full‐matrix least‐squares method on all *F* ^2^ data by using the SHELXL^[^ ^53^ ^]^ incorporated in the OLEX2 program.^[^ ^54^ ^]^ All hydrogen atoms, including those located in the difference density map, were placed in calculated positions and refined as the riding model. Crystallographic details are collected in Tables S2–S6 (Supporting Information). CCDC 2 284 427, 2 284 430, 2 284 431, 2 284 433, and 2 284 434 contain supplementary crystallographic data for this paper. These data can be obtained free of charge from The Cambridge Crystallographic Data Centre via www.ccdc.cam.ac.uk/data_request/cif.

### Syntheses of Precursors

**NCP** ligands^[^ ^55^ ^]^ and metalloligands **NiMeP** ^[^ ^29^, ^30^, ^33^ ^]^ and **RuSPy** ^[^ ^34^ ^]^ were obtained by the literature methods.

### Synthesis of ClNCPIrCp*

A sample of 0.040 g (0.054 mmol) **ClNCP**, 0.044 g (0.054 mmol) dichloro(pentamethylcyclopentadienyl)iridium(III) dimer, 0.044 g (0.540 mmol) anhydrous sodium acetate and 20 mL dichloromethane were placed in a two‐necked flask. The reaction mixture was purged for 20 min with N_2_ and then refluxed for 12 h. After this time, the reaction mixture was filtered, and the solution was concentrated and separated on a silica‐gel column. The compound was eluted using 0.5% MeOH in dichloromethane. The collected fraction was evaporated to dryness and recrystallized from the dichloromethane/*n*‐hexane system. Yield 0.049 g (83%).

### Selected Data for **ClNCP**IrCp*

^1^H NMR (500 MHz, CDCl_3_, *δ*): 9.36 (d, *J* = 4.8 Hz, 1H, pyrrH), 8.56 (d, *J* = 4.7 Hz, 1H, pyrrH), 8.48 (d, *J* = 5.0 Hz, 1H, pyrrH), 8.31 (AB, *J* = 4.8 Hz, 1H, pyrrH), 8.30 (AB, *J* = 4.8 Hz, 1H, pyrrH), 8.28 (s, 1H, pyrrH), 8.26 (d, ^4^ *J* = 2.3 Hz, 1H, ArH), 8.21 (d, *J* = 4.8 Hz, 1H, pyrrH), 8.07 (td, *J* = 8.4 Hz, ^4^ *J* = 2.0 Hz, 2H, ArH), 8.04 (d, *J* = 8.4 Hz, 2H, ArH), 7.79 (td, *J* = 8.4 Hz, ^4^ *J* = 2.3 Hz, 3H, ArH), 7.75 (dd, *J* = 8.2 Hz, ^4^ *J* = 2.2 Hz, 2H, ArH), 7.71 (dd, *J* = 8.1 Hz, ^4^ *J* = 2.2 Hz, 1H, ArH), 7.62–7.66 (overlapping multiplets, 3H, ArH), 7.27 (dd, *J* = 8.1 Hz, ^4^ *J* = 2.3 Hz, 1H, ArH), 0,85 (s, 15H, Cp*H), –1.08(br, 2H, NH), –4.69 (s, 1H, ‐CH). ^13^C NMR (150 MHz, CDCl_3_, *δ_C_*): 159.1, 157.6, 155.4, 149.5, 141.8, 141.6, 140.7, 139.4, 139.3, 138.2, 138.1, 136.9, 136.6, 136.55, 136.49, 136.0, 135.6, 135.5, 135.4, 135.1, 134.9, 134.8, 134.7, 134.5, 134.4, 134.0, 131.2, 129.8, 128.9, 127.72, 127.65, 127.6, 127.5, 127.4, 127.2, 125.4, 124.4, 117.7, 115.3, 88.0, 85.1 (*C*21), 8.3. HRMS (ESI) *m/z*: [M─Cl]^+^ calcd for C_54_H_40_N_4_Cl_4_Ir, 1079.1631; found, 1079,1638. UV–vis (CH_2_Cl_2_) *λ*_max_, (ε/10^4^ [M^−1^ cm^−1^]) = 299 (3.12), 335 (sh), 391 (4.01), 402 (sh), 455 (12.57), 550(sh), 564 (1.79), 606 (1.31), 642(sh), 794 (1.41).

### Synthesis of NiMePIrCp*

A sample of 0.025 g (0.036 mmol) of **NiMeP**, along with 0.016 g (0.020 mmol) of dichloro(pentamethylcyclopentadienyl)iridium(III) dimer, 0.016 g (0.2 mmol) anhydrous sodium acetate and 10 mL of dichloromethane were placed in a two‐neck round bottom flask. The mixture was purged for 20 min with N_2_ and then refluxed for 18 h. In the next stage, The reaction mixture was then filtered, concentrated, and passed down a silica gel column. The compound was eluted with 30% ethyl acetate in *n*‐hexane. The collected fraction was evaporated to dryness and recrystallized from the dichloromethane/*n*‐hexane. Yield 0.035 g (93%).

### Selected Data for NiMePIrCp*

^1^H NMR (500 MHz, CDCl_3_, *δ*): 9.25 (AB, *J* = 5.1 Hz, 1H, pyrrH), 8.98 (s, 1H, pyrrH), 8.45 (AB, *J* = 5.0 Hz, 1H, pyrrH), 8.30 (AB, *J* = 5,0 Hz, 1H, pyrrH), 8.29 (AB, *J* = 5.1 Hz, 2H, pyrrH), 8.28 (AB, *J* = 5.0 Hz, 1H, pyrrH), 8.20 (dd, ^3^ *J* = 7.5 Hz, ^4^ *J* = 1.4 Hz, 1H, ArH), 8.15 (br, 4H, ArH), 7.64–7.75 (overlapping multiplets, 10H, ArH), 7.61 (br, 3H, ArH), 7.28 (td, ^3^ *J* = 7.4 Hz, ^4^ *J* = 1.5 Hz, 1H, ArH), 7.24 (m, 1H, ArH), 0.7 (s, 15H, ─CH_3_(Cp*H)), –2.23 (s, 3H, ─CH_3_). ^13^C NMR (150 MHz, CDCl_3_, *δ_C_*): 170.5, 154.2, 151.9, 151.8, 151.7, 151.5, 150.1, 148.8, 147.5, 145.9, 141.2, 140.7, 140.6, 139.5, 136.0, 135.2, 134.9, 133.6, 133.5, 133.4, 133.3, 132.9, 129.2, 128.7, 128.6, 128.1, 127.9, 127.3, 127.1, 124.7, 120.3, 119.8, 87.8, 28.2, 8.1. HRMS (ESI) *m/z*: [M─Cl]^+^ calcd for: C_55_H_44_N_4_NiIr, 1011,2543; found, 1011,2540. UV–vis (CH_2_Cl_2_) *λ*_max_, (ε/10^4^ [M^−1^ cm^−1^]) = 294(3.57), 341(3.20), 392(sh), 439(4.29), 486(sh), 568(sh), 676 (0.7), 743(sh).

### Synthesis of NiMePRuCym

A sample of 0.020 g (0.030 mmol) of **NiMeP**, 0.009 g (0.015 mmol) dichloro(*p*‐cymene)ruthenium(II) dimer, 0.012 g (0.150 mmol) anhydrous sodium acetate were placed in a two‐neck flask and 10 mL of dichloromethane were added. The mixture was purged for 20 min. with N_2_, and then refluxed for 48 h. After that, the mixture was filtered, concentrated, and subjected to silica‐gel column chromatography. The compound was eluted with 30% ethyl acetate in *n*‐hexane. The collected fraction was evaporated to dryness and recrystallized from the dichloromethane/*n*‐hexane. Yield 0.017 g (60%).

### Selected Data for NiMePRuCym

^1^H NMR (500 MHz, CDCl_3_, *δ*): 9.47 (s, 1H, pyrrH), 9.30 (AB, *J* = 5.1 Hz, 1H, pyrrH), 8.49 (AB, *J* = 4.9 Hz, 1H, pyrrH), 8.36 (dd, ^3^ *J* = 7.6 Hz, ^4^ *J* = 1.0 Hz, 1H, ArH), 8.33 (AB, *J* = 5.1 Hz, 1H, pyrrH), 8.32 (AB, *J* = 4.8 Hz, 1H, pyrrH), 8.30 (AB, *J* = 4.9 Hz, 1H, pyrrH), 8.28 (AB, *J* = 4.9 Hz, 1H, pyrrH), 8.13 (br, 3H, ArH), 7.50–7.79 (overlapping multiplets + br, 13H, ArH), 7.30 (td, ^3^ *J* = 7.3 Hz, ^4^ *J* = 1.1 Hz, 1H, ArH), 7.23 (td, ^3^ *J* = 7.2 Hz, ^4^ *J* = 1.4 Hz, 1H, ArH), 4.97 (d, *J* = 5.8 Hz, 1H, ArH(*p*Cym)), 4.77 (AB, *J* = 5.8 Hz, 1H, ArH(*p*Cym)), 4.58 (AB, *J* = 5.8 Hz, 1H, ArH(*p*Cym)), 4.08 (d, *J* = 5.8 Hz, 1H, ArH(*p*Cym)), 1.84 (sep, *J* = 6.9 Hz, 1H, ─CH─(*p*Cym)), 0.82 (s, 3H, ─CH_3_(*p*Cym)), 0.69 (d, *J* = 6.9 Hz, 3H, ─CH_3_(*p*Cym)), 0.38 (d, *J* = 6.9 Hz, 3H, ─CH_3_(*p*Cym)), –2.39 (s, 3H, ─CH_3_). ^13^C NMR (150 MHz, CDCl_3_ *δ_C_*): 174.0, 170.7, 152.0, 151.8, 151.2, 151.0, 149.9, 149.3, 148.7, 147.5, 144.1, 140.69, 140.66, 139.6, 136.8, 135.8, 135.5, 134.9, 134.7, 134.1, 133.6, 133.54, 133.46, 132.8, 129.1, 128.6, 128.2, 128.1, 127.9, 127.1, 125.3, 124.4, 120,.9, 119.9, 104.7, 96.3, 88.7, 86.8, 84.73, 84.71, 29.9, 22.0, 21.9, 17.9, 15.6. HRMS (ESI) *m/z*: [M–Cl]^+^ calcd for C_55_H_43_N_4_NiRu, 919.1879; found, 919.1871. UV–vis (CH_2_Cl_2_) *λ*_max_, (ε/10^4^ [M^−1^ cm^−1^]) = 295(2.76), 343(2.58), 392(sh), 439(4.10), 525(sh), 655(sh) 738(sh).

### Synthesis of NiMePRhCp*

The compound was synthesized and purified in the same way as **NiMeP**RuCym, except that dichloro(p‐cymene)ruthenium(II) was replaced by dichloro(pentamethylcyclopentadienyl)rhodium(III) dimer (0.009 g, (0.015 mmol)). Yield 0.011 g (40%).

### Selected Data for NiMePRhCp*

^1^H NMR (500 MHz, CDCl_3_, *δ*): 9.29 (AB, *J* = 5.2 Hz, 1H, pyrrH), 9.19 (s, 1H, pyrrH), 8,.47 (AB, *J* = 5.1 Hz, 1H, pyrrH), 8.35 (AB, *J* = 5.1 Hz, 1H, pyrrH), 8.33 (AB, *J* = 5.0 Hz, 1H, pyrrH), 8.32 (AB, *J* = 4.9 Hz, 1H, pyrrH) 8.30 (AB, *J* = 4.7 Hz, 1H, pyrrH) 8.25 (dd, ^3^ *J* = 7.8 Hz, ^4^ *J* = 1.1 Hz, 1H, ArH), 8.16 (br, 3H, ArH), 7.65–7.74 (overlapping multiplets, 9H, ArH), 7.62 (br, 4H, ArH), 7.32 (td, *J* = 7.2, ^4^ *J* = 1.4, 1H, ArH), 7.27 (td, ^3^ *J* = 7.4 Hz, ^4^ *J* = 1.5 Hz, 1H, ArH), 0.69 (s, 15H, Cp*H), –2.36 (s, 3H, ─CH_3_). ^13^C NMR (150 MHz, CDCl_3_, *δ_C_*): 171.5, 169.9, 169.6, 152.44, 152.37, 152.0, 151.1, 150.0, 148.9, 147.3, 146.1, 140.8, 140.64, 140.58, 139.5, 136.3, 135.9, 135.30, 135.25, 134.3, 133.8, 133.6, 133.50, 133.47, 132.9, 129.2, 128.6, 128.1, 128.0, 127.5, 127.1, 126.6, 124.6, 121.0, 120.0, 95.40, 95.36, 15.2, 8.3. HRMS (ESI) *m/z*: [M–Cl]^+^ calcd for C_55_H_44_N_4_NiRh, 921.1969; found, 921,1963. UV–vis (CH_2_Cl_2_) *λ*_max_, (ε/10^4^ [M^−1^ cm^−1^]) = 293 (3.03), 336 (2.78), 392(sh), 442 (3.73), 606(sh) 656(sh), 734(sh).

### Synthesis of RuSPyIrCp*

The compound was synthesized and purified in the same way as **NiMeP**IrCp*. A sample of 0.020 g (0.024 mmol) of compound **RuSPy**, 0.010 g (0.012 mmol) dichloro(pentamethylcyclopentadienyl)iridium(III) dimer and 0.010 g (0.120 mmol) anhydrous sodium acetate were used. Yield 0.025 g (85%).

### Selected Data for RuSPyIrCp*

^1^H NMR (500 MHz, CDCl_3_, *δ*): 8.86 (AB, *J* = 5.1 Hz, 1H, pyrrH), 8,34 (AB, *J* = 5.1 Hz, 1H, pyrrH), 8.31 (AB, *J* = 5.1 Hz, 1H, pyrrH), 8.30 (AB, *J* = 5.2 Hz, 1H, pyrrH), 8.20 (dd, ^3^ *J* = 7.5 Hz, ^4^ *J* = 1.4 Hz, 1H, ArH), 8.15 (br, 2H, ArH), 8.14 (s, 1H, pyrrH), 8.13 (AB, *J* = 5.2 Hz, 1H, pyrrH), 8.08–8.10 (m, 1H, ArH), 8.02–8.04 (m, 1H, ArH), 8.01 (AB, *J* = 5.2 Hz, 1H, pyrrH), 7.79–7.80 (overlapping multiplets, 1H, ArH), 7.64–7.75 (overlapping multiplets, 8H, ArH), 7.54–7.62 (overlapping multiplets, 3H, ArH), 7.23 (td, ^3^ *J* = 7.4 Hz, ^4^ *J* = 1.7 Hz, 1H, ArH), 7.19 (td, ^3^ *J* = 7.4 Hz, ^4^ *J* = 1.7 Hz, 1H, ArH), 6.21 (td, ^3^ *J* = 7.7 Hz, ^4^ *J* = 1.6 Hz, 1H, pyH), 5.61 (d, *J* = 8.1 Hz, 1H, pyH), 5.44 (td, ^3^ *J* = 6.7 Hz, ^4^ *J* = 1.3 Hz, 1H, pyH), 3.00 (dq, ^3^ *J* = 6.0 Hz, ^4^ *J* = 0.8 Hz, 1H, pyH), 0.97 (s, 15H, Cp*H). ^13^C NMR (150 MHz, CDCl_3_, *δ_C_*): 195.5, 170.3, 167.5, 154.6, 154.1, 152.2, 151.9, 151.3, 150.7, 145.7, 144.5, 142.9, 141.4, 141.3, 141.2, 140.5, 138.5, 137.9, 137.1, 135.9, 135.3, 135.1, 134.9, 134.1, 133.94, 133.92, 133.8, 133.7, 133.6, 133.5, 133.2, 132.2, 128.9, 128.3, 127.7, 127.6, 127.03, 126.99, 126.92, 126.85, 126.7, 124.5, 121.6, 120.6, 117.0, 116.5, 88.2, 8.4. HRMS (ESI) *m/z*: [M─Cl]^+^ calcd for C_60_H_45_N_5_OSRuIr, 1178.2012; found, 1178.2014. UV–vis (CH_2_Cl_2_) *λ*_max_, (ε/10^4^ [M^−1^ cm^−1^]) = 302 (3.28), 367(sh), 433 (3.26), 498 (sh), 510 (2.51), 604(sh), 728 (1.41).

### Synthesis of RuSPyRuCym

The compound was synthesized and purified in the same way as **NiMeP**RuCym. A sample of 0.020 g (0.024 mmol) of compound **RuSPy**, 0.007 g (0.012 mmol) dichloro(*p*‐cymene) ruthenium(II) dimer and 0.010 g (0.120 mmol) of anhydrous sodium acetate were used. Yield 0.014 g (54%).

### Selected Data for RuSPyRuCym

^1^H NMR (500 MHz, CDCl_3_, *δ*): 8,91 (AB, *J* = 5.2 Hz, 1H, pyrrH), 8.52 (s, 1H, pyrrH), 8.38 (AB, *J* = 5.0 Hz, 1H, pyrrH), 8,37 (dd, ^3^ *J* = 7.5 Hz, ^4^ *J* = 1.3 Hz, 1H, ArH), 8.34 (AB, *J* = 5.0 Hz, 2H, pyrrH), 8.15–8.17 (overlapping multiplets, 2H, ArH), 8.15 (AB, *J* = 5.1 Hz, 1H, pyrrH), 8.06–8.09 (m, 1H, ArH), 8.03 (AB, *J* = 5.2 Hz, 1H, pyrrH), 7.95–7.97 (m, 1H, ArH), 7.78 (m, 1H, ArH), 7.56–7.75 (overlapping multiplets, 12H, ArH), 7.31 (td, ^3^ *J* = 7.3 Hz, ^4^ *J* = 1.3 Hz, 1H, ArH), 7.22 (td, ^3^ *J* = 7.4 Hz, ^4^ *J* = 1.4 Hz, 1H, ArH), 6.20 (td, ^3^ *J* = 7.7 Hz, ^4^ *J* = 1.7 Hz, 1H, pyH), 5.60 (d, *J* = 8,.1 Hz, 1H, pyH), 5,.43 (td, *J* = 6.7 Hz, ^4^ *J* = 1.0 Hz, 1H, pyH), 4.87 (d, *J* = 5.9 Hz, 1H, ArH(*p*Cym)), 4.48 (d, *J* = 6.0 Hz, 2H, ArH(*p*Cym)), 3.05 (d, *J* = 5.8 Hz, 1H, ArH(*p*Cym)), 2.92 (d, *J* = 6.0 Hz, 1H, pyH), 2.47 (sep, *J* = 7.0 Hz, 1H, ─CH─(*p*Cym)), 1.53 (s, 3H, ─CH_3_(*p*Cym)), 0.81 (d, *J* = 6.8 Hz, 3H, ─CH_3_(*p*Cym)), 0.73 (d, *J* = 7.1 Hz, 3H, ─CH_3_(*p*Cym)). ^13^C NMR (125 MHz, CDCl_3_, *δ_C_*): 195.8, 175.2, 172.6, 167.7, 154.9, 151.8, 151.5, 151.2, 150.5, 145.5, 144.5, 144.0, 142.7, 141.4, 141.3, 140.4, 140.3, 138.5, 138.0, 136.0, 135.3, 135.2, 134.5, 134.02, 133.96, 133.94, 133.8, 133.6, 133.54, 133.49, 133.0, 132.2, 128.9, 128.2, 127.7, 127.6, 126.98, 126.95, 126.8, 126.7, 125.2, 124.2, 121.9, 117.0, 116.5, 110.8, 97.8, 88.3, 86.8, 84.5, 30.2, 23.4, 20.9, 18.6. HRMS (ESI) *m/z*: [M─Cl]^+^ calcd for C_60_H_44_N_5_OSRu_2_, 1086.1348; found, 1086.1343; [M + Na]^+^ calcd for C_60_H_44_N_5_OSClRu_2_Na, 1144,0934; found, 1144,0936. UV–vis (CH_2_Cl_2_) *λ*_max_, (ε/10^4^ [M^−1^ cm^−1^]) = 300 (3.41), 366(sh), 424 (3.38), 459(sh), 538 (1.70), 625(sh), 723 (1.40).

### Synthesis of RuSPyRhCp*

A sample of 0.020 g (0.024 mmol) of compound **RuSPy**, 0.007 g (0.012 mmol) dichloro(pentamethylcyclopentadienyl)rhodium(III) dimer, 0.010 g (0.120 mmol) of anhydrous sodium acetate and 10 mL of chloroform was placed in a two‐neck round‐bottom flask. The mixture was purged with N_2_ gas for 20 min and then refluxed for 24 h. After this time, another portion of 0.007 g (0.012 mmol) of dichloro(cyclopentadienyl) rhodium(III) dimer and 0.010 g (0.120 mmol) of anhydrous sodium acetate was added and heated for another 24 h under reflux. In the next stage, the mixture was filtered, concentrated and separated by a silica gel chromatographic column. The compound was eluted with 30–35% ethyl acetate in *n*‐hexane. The collected fraction was evaporated to dryness and recrystallized from the dichloromethane/*n*‐hexane. Yield 0.009 g (35%).

### Selected Data for RuSPyRhCp*

^1^H NMR (500 MHz, CDCl_3_, *δ*): 8.90 (AB, *J* = 5.2 Hz, 1H, pyrrH), 8.35 (AB, *J* = 5.2 Hz, 1H, pyrrH), 8.33 (AB, *J* = 5.0 Hz, 1H, pyrrH), 8.32 (AB, *J* = 5.0 Hz, 1H, pyrrH), 8.30 (s, 1H, pyrrH), 8.24 (dd, *J* = 7.8 Hz, 1H, ArH), 8.16 (AB, *J* = 5.2 Hz, 1H, pyrrH), 8.14–8.17 (overlapping multiplets, 2H, ArH), 8.09–8.11 (m, 1H, ArH), 8.03–8.05 (overlapping multiplets, 1H, ArH), 8.03 (AB, *J* = 4.9 Hz, 1H, pyrrH), 7.79–7.80 (m, 1H, ArH), 7.64–7.76 (overlapping multiplets, 8H, ArH), 7.54–7.62 (overlapping multiplets, 3H, ArH), 7.28 (td, ^3^ *J* = 7.4 Hz, ^4^ *J* = 1.2 Hz, 1H, ArH), 7.23 (td, ^3^ *J* = 7.4 Hz, ^4^ *J* = 1.4 Hz, 1H, ArH), 6.20 (td, ^3^ *J* = 7.8 Hz, ^4^ *J* = 1.7 Hz, 1H, pyH), 5.60 (d, *J* = 8.0 Hz, 1H, pyH), 5.43 (td, *J* = 6.6 Hz, ^4^ *J* = 1.2 Hz, 1H, pyH), 2.96 (d, *J* = 6.0 Hz, 1H, pyH), 0.9 (s, 15H, Cp*). ^13^C NMR (150 MHz, CDCl_3_, *δ_C_*): 195.3, 172.1, 170.0, 169.8, 167.5, 155.0, 152.6, 152.0, 151.0, 150.7, 145.7, 144.3, 142.8, 141.3, 141.2, 140.7, 140.4, 139.0, 138.7, 136.6, 136.4, 135.9, 135.5, 134.8, 134.2, 134.0, 133.9, 133.7, 133.6, 133.5, 133.2, 131.0, 128.9, 128.3, 127.74, 127.66, 127.0, 126.9, 126.8, 126.7, 126.4, 124.4, 122.2, 120.6, 117.0, 116.5, 95.83, 95.79, 8.6. HRMS (ESI) *m/z*: [M─Cl]^+^ calcd for C_60_H_45_N_5_OSRuRh, 1088,1438; found, 1088,1432. UV–vis (CH_2_Cl_2_) *λ*_max_, (ε/10^4^ [M^−1^ cm^−1^]) = 303 (3.04), 369(sh), 439(3.43), 492(sh), 506(2.67), 598(sh), 719(1.30).

## Conflict of Interest

The authors declare no conflict of interest.

## Supporting information

Supporting Information

## Data Availability

The data that support the findings of this study are available in the supplementary material of this article.
