# Supplementary material for: Complexes of N‐Confused Porphyrin Derivatives as Ortho‐Metallating Ligands. Synthesis, Structure, Redox Properties, and Chirality
Source: Adv Sci (Weinh). 2023 Nov 21;11(2):2306696. doi: 10.1002/advs.202306696 (PMC10787092; doi:10.1002/advs.202306696)
Supplement: Supplementary file 1 — Supporting Information [file ADVS-11-2306696-s001.pdf]

ADVANCED SCIENCE
Open Access

Supporting Information

for *Adv. Sci.*, DOI 10.1002/advs.202306696

Complexes of N-Confused Porphyrin Derivatives as *Ortho*-Metallating Ligands. Synthesis, Structure, Redox Properties, and Chirality

*Sebastian Koniarz, Kinga Szydełko, Michał J. Białek, Karolina Hurej and Piotr J. Chmielewski**

Supporting Information

Complexes of N-confused Porphyrin Derivatives as Ortho-metallating Ligands. Synthesis, Structure, Redox Properties, and Chirality

*Sebastian Koniarz, Kinga Szydełko, Michał Białek, Karolina Hurej, and Piotr J. Chmielewski**

Department of Chemistry, University of Wrocław, 14 F. Joliot-Curie, Wrocław, 50 383, Poland.

E-mail: piotr.chmielewski@uwr.edu.pl

Table of contenst

Figure S1 Optical spectra of **ClNCP**IrCp* and **ClNCP**
Figure S2-S3B NMR spectra of **NiMeP**IrCp*
Figure S4-S5**B** NMR spectra of **NiMeP**RuCym
Figure S6-S7B NMR spectra of **NiMeP**RhCp*
Figure S8-S9B NMR spectra of **RuSPy**IrCp*
Figure S10-S11B NMR spectra of **RuSPy**RuCym
Figure S12-S13B NMR spectra of **RuSPy**RhCp*
Figure S14-S15B NMR spectra of **ClNCP**IrCp*
Figure S16. High-resolution mass spectra of **NiMeP**IrCp*
Figure S17. High-resolution mass spectra of **NiMeP**RuCym
Figure S18. High-resolution mass spectra of **NiMeP**RhCp*
Figure S19. High-resolution mass spectra of **RuSPy**IrCp*
Figure S20. High-resolution mass spectra of **RuSPy**RuCym
Figure S21. High-resolution mass spectra of **RuSPy**RhCp*
Figure S22. High-resolution mass spectra of **4ClNCP**IrCp*
Figure S23. Experimental and calculated absorbance and CD spectra of **RuSPy**
Figure S24. Experimental and calculated absorbance and CD spectra of **RuSPy**IrCp*
Figure S25. Experimental and calculated absorbance and CD spectra of **NiMeP**IrCp*
Figure S26. Experimental and calculated absorbance and CD spectra of **NiMeP**RuCym
Figure S27. Experimental and calculated absorbance and CD spectra of **ClNCP**IrCp*
Figure S28. CD spectra of enantiomers of **ClNCP**IrCp*
Figure S29. HPLC profiles for **NiMeP**RhCp* on a chiral stationary phase and CD spectra of enantiomers
Figure S30. HPLC profiles for **RuSPy**IrCp* on chiral stationary phase and CD spectra of enantiomers of **RuSPy**RhCp* and **RuSPy**IrCp*
Figure S31. Spectrophotometric titration of **ClNCP**IrCp* with BAHA
Figure S32. Spectrophotometric titration of **NiMeP**RuCym with BAHA
Figure S33. Spectrophotometric titration of **NiMeP** with BAHA
Figure S34. Spectrophotometric titration of of **RuSPy**RhCp* with BAHA
Figure S35. EPR-monitored titration of **NiMeP**RhCp* with BAHA
Figure S36. EPR-monitored titration of **NiMeP**RuCym with BAHA
Figure S37. EPR-monitored titration of **NiMeP** with BAHA
Figure S38. Voltamogramms of **ClNCP**
Computational methods
Table S1. Computational details for the optimized structures of compounds
Crystallographic data
Table S2. Crystal data for **NiMeP**IrCp*

Table S3. Crystal data for **NiMeP**RuCym
Table S4. Crystal data for **RuSPy**IrCp*
Table S5. Crystal data for **RuSPy**RhCp*
Table S6. Crystal data for **ClNCP**IrCp*
Figure S39-S43 ORTEP drawing of the asymmetric parts of the crystal structures
Table S7. Electronic transitions calculated for **RuSPy** by means of TD DFT
Table S8. Electronic transitions calculated for **RuSPy**IrCp* by means of TD DFT
Table S9. Electronic transitions calculated for **NiMeP**RuCym by means of TD DFT
Table S10. Electronic transitions calculated for **RuSPy**RhCp* by means of TD DFT
Table S11. Electronic transitions calculated for **NiMeP**IrCp* by means of TD DFT
Table S12. Electronic transitions calculated for **ClNCP**IrCp* by means of TD DFT
References

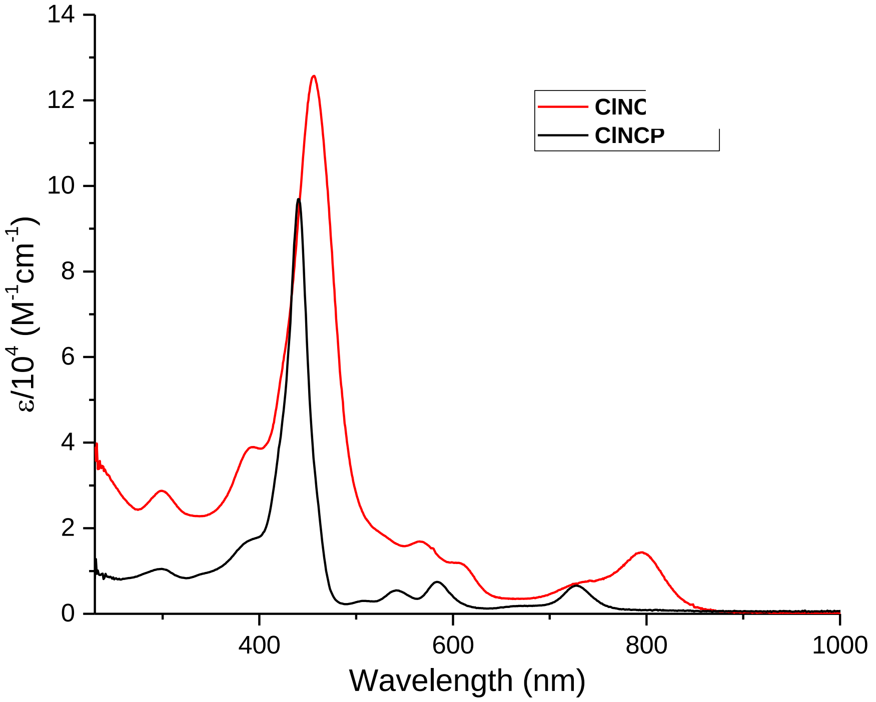

Figure S1. Optical spectra (DCM, 298 K) of **ClNCP**IrCp* (red trce) and **ClNCP** (black trace).

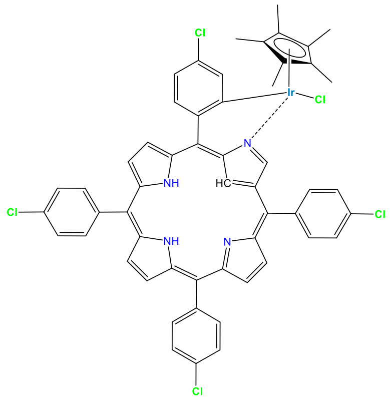

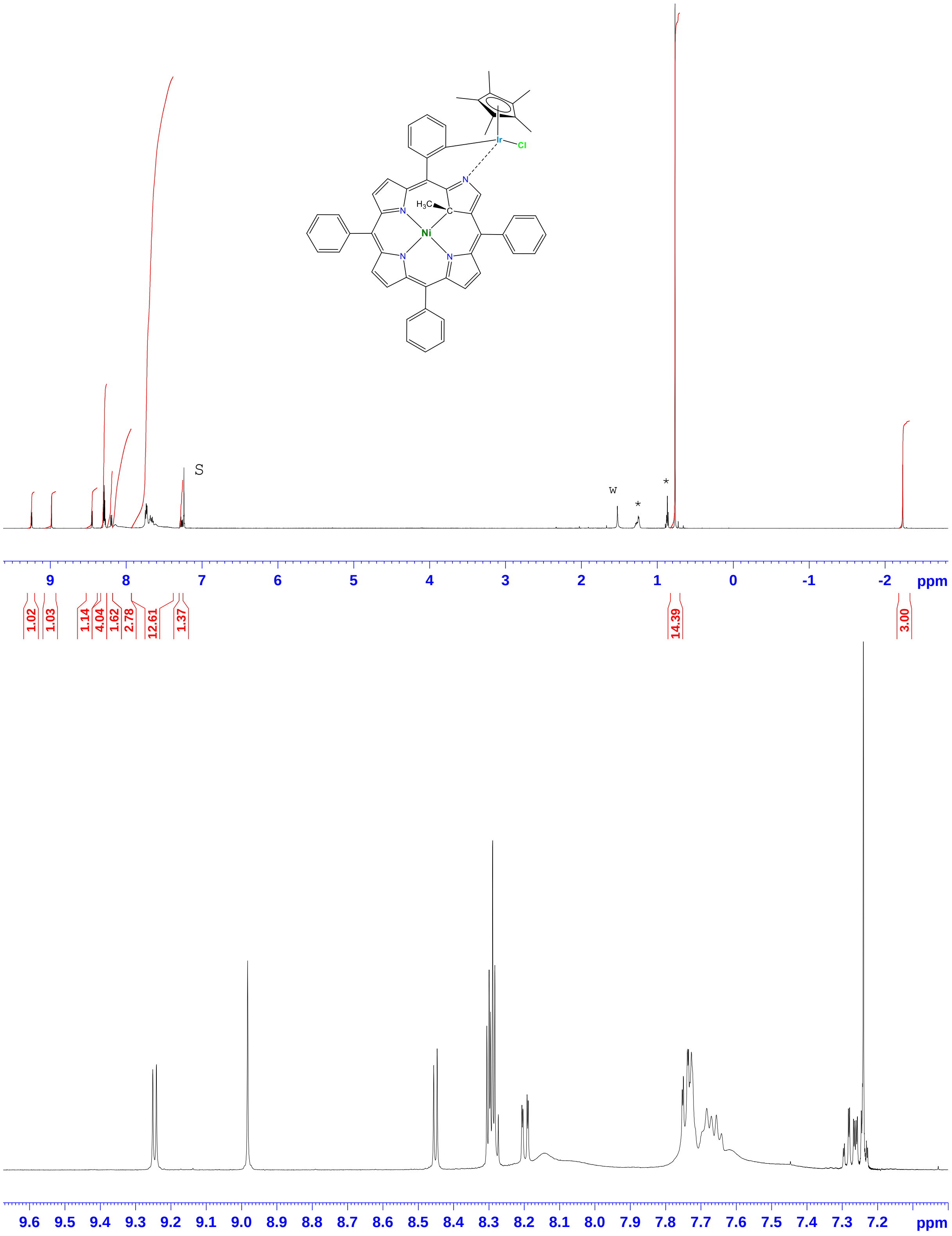

Figure S2. ^{1}H NMR spectrum (500 MHz, $CDCl_3$, 300 K) of **NiMeP**IrCp* (top) and expansion of the low-field region of this spectrum (bottom). s, residual $CHCl_3$ signal; w, dissolved water signal. The signal of impurities are marked with asterisks.

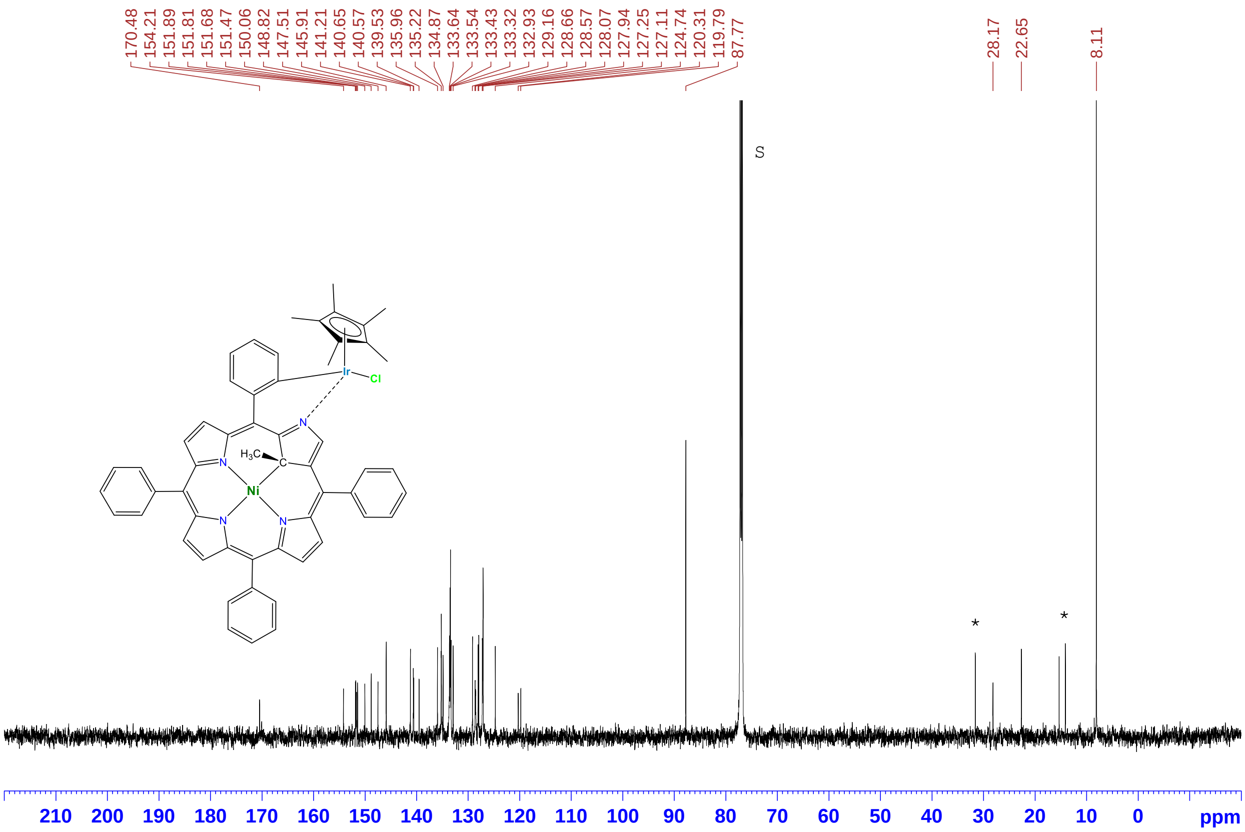

Figure S3. ^{13}C NMR spectrum (150 MHz, $CDCl_3$, 300 K) of **NiMeP**IrCp* s, $CDCl_3$ signal. The signal of impurities are marked with asterisks.

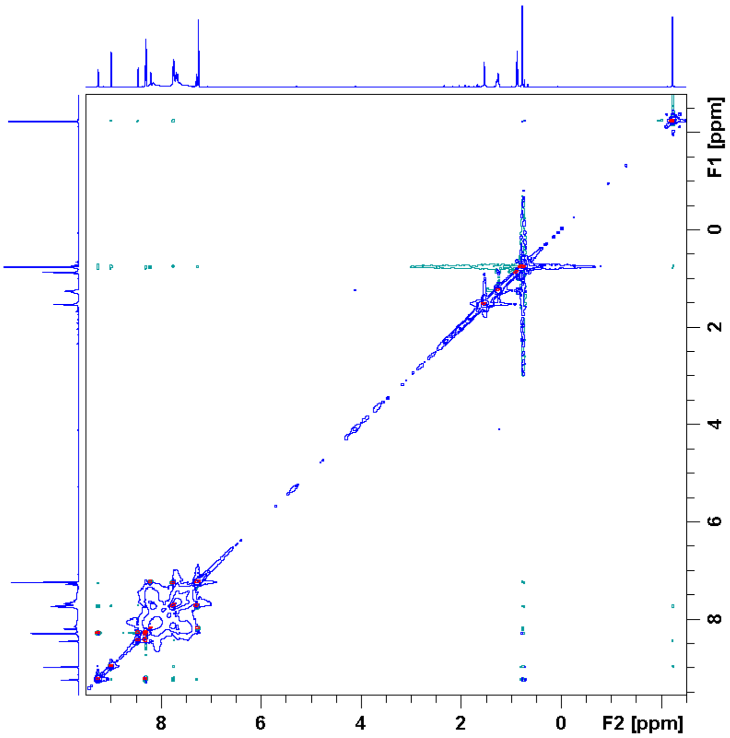

Figure S3A. Superimposed ^{1}H,^{1}H NOESY (blue and green crosspeaks) and ^{1}H,^{1}H COSY (red crosspeaks) spectra (600 MHz, $CDCl_3$, 300 K) of **NiMeP**IrCp*.

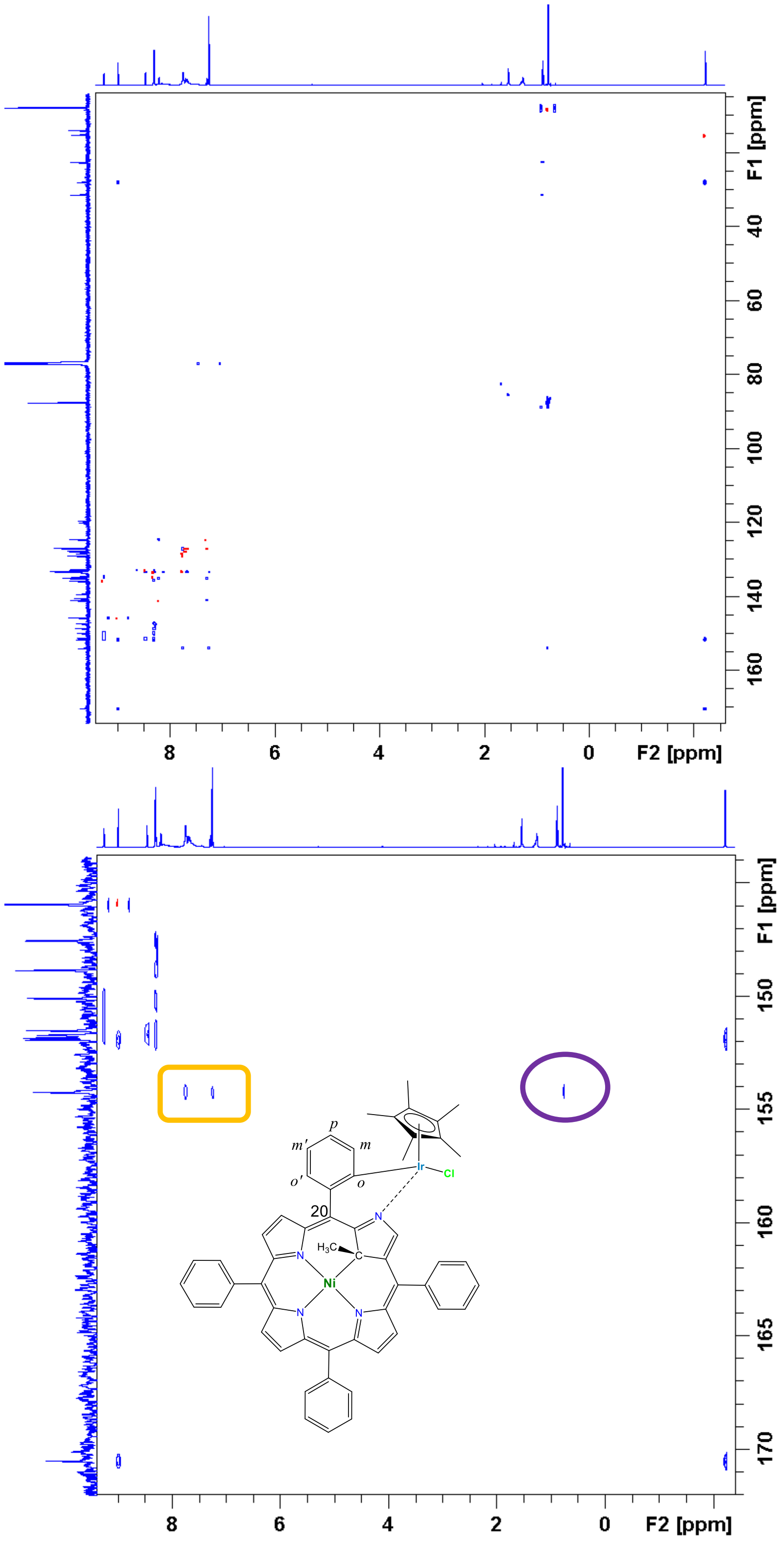

Figure S3B. Superimposed $^{13}C,^{1}H$ HMBC (blue crosspeaks) and $^{13}C,^{1}H$ HSQC (red crosspeaks) spectra (600/150 MHz, $CDCl_3$, 300 K) of **NiMeP**IrCp* (top) and expansion of a selected region of the HMBC map (bottom) showing correlations of the iridium(III)-coordinated *ortho*-C with *o*'- and *p*-20-Ph protons (golden frame) and with Cp* methyl protons (purple oval).

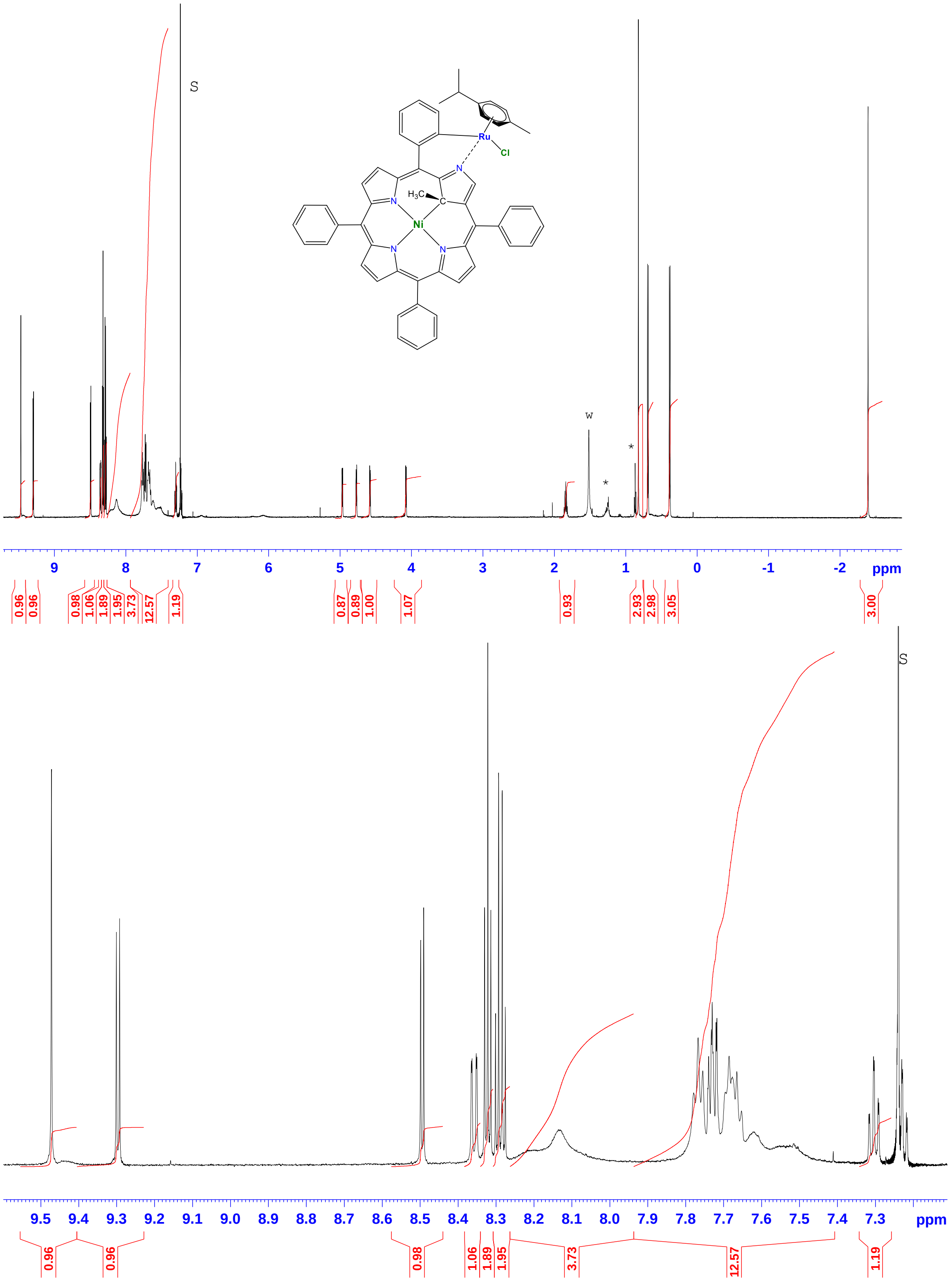

Figure S4. ^{1}H NMR spectrum (500 MHz, $CDCl_3$, 300 K) of **NiMeP**RuCym (top) and expansion of the low-field region of this spectrum (bottom). s, residual $CHCl_3$ signal; w, dissolved water signal. The signal of impurities are marked with asterisks.

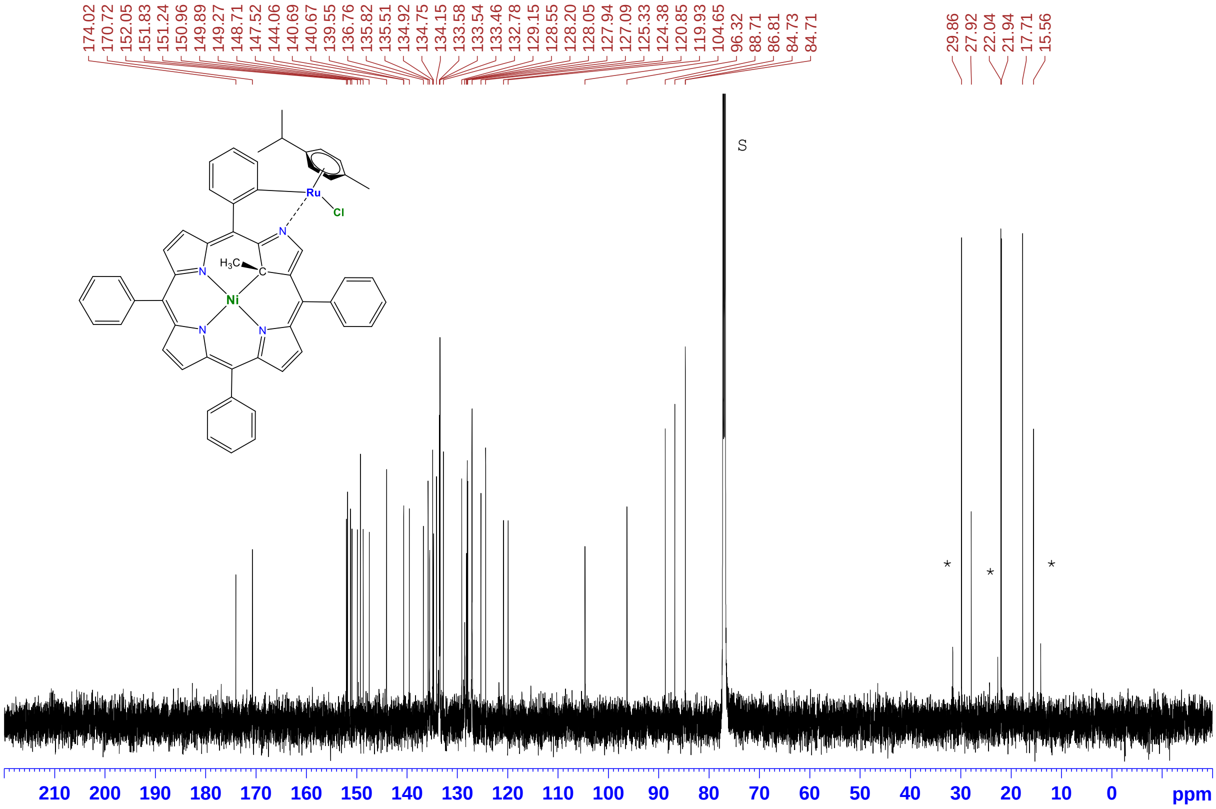

Figure S5. ^{13}C NMR spectrum (150 MHz, $CDCl_3$, 300 K) of **NiMeP**RuCym. s, $CDCl_3$ signal. The signal of impurities are marked with asterisks.

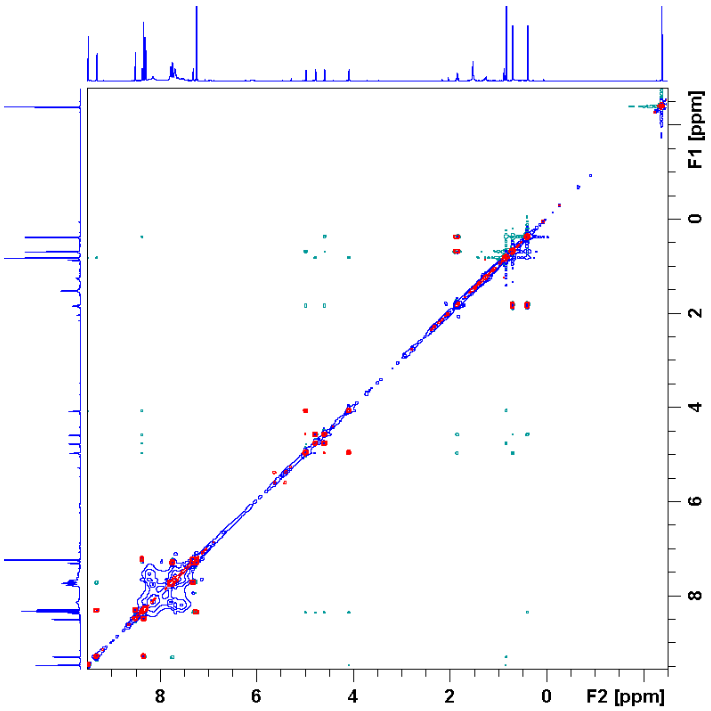

Figure S5A. Superimposed ^{1}H,^{1}H NOESY (blue and green crosspeaks) and ^{1}H,^{1}H COSY (red crosspeaks) spectra (600 MHz, $CDCl_3$, 300 K) of **NiMeP**RuCym.

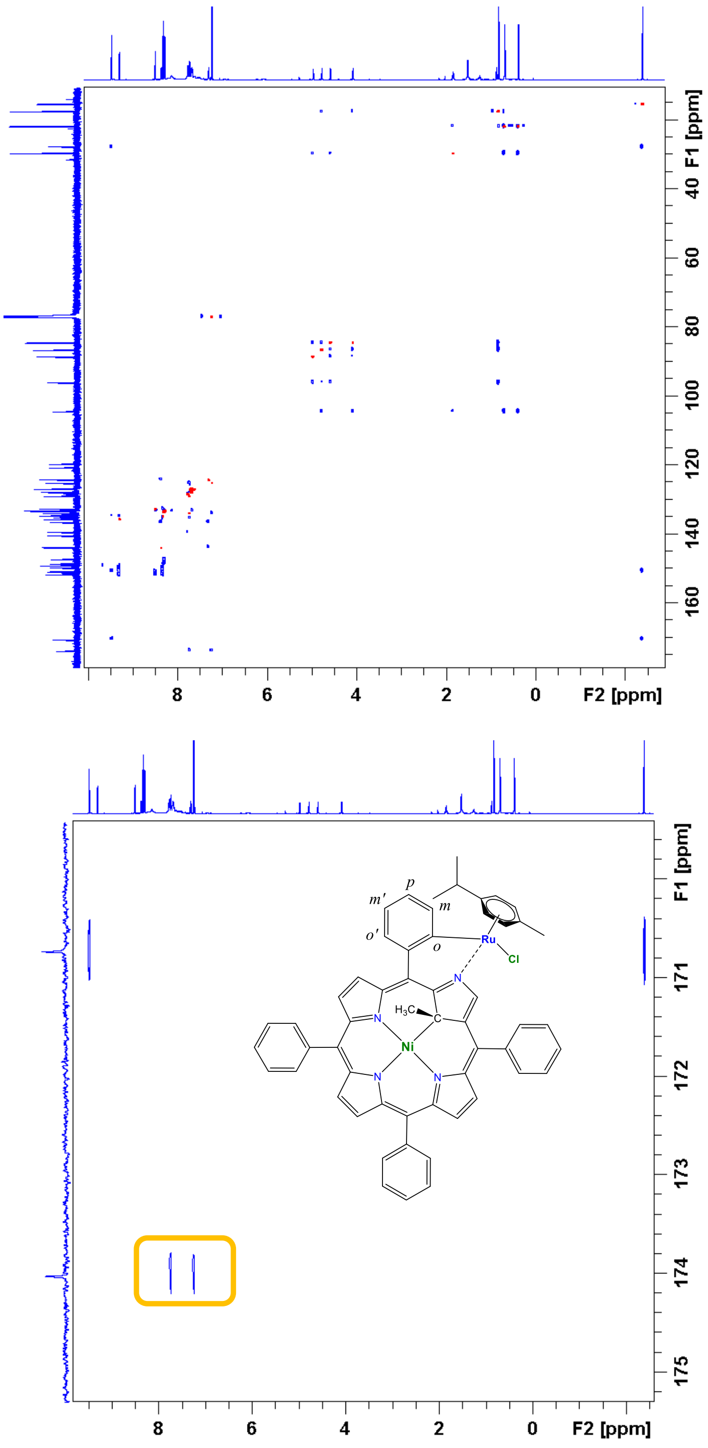

Figure S5B. Superimposed $^{13}C,^{1}H$ HMBC (blue crosspeaks) and $^{13}C,^{1}H$ HSQC (red crosspeaks) spectra (600/150 MHz, $CDCl_3$, 300 K) of **NiMeP**RuCym (top) and expansion of the low-field region of the HMBC map (bottom) showing correlations of the ruthenium(II)-coordinated *ortho*-C with *o'*- and *p*-20-Ph protons (golden frame).

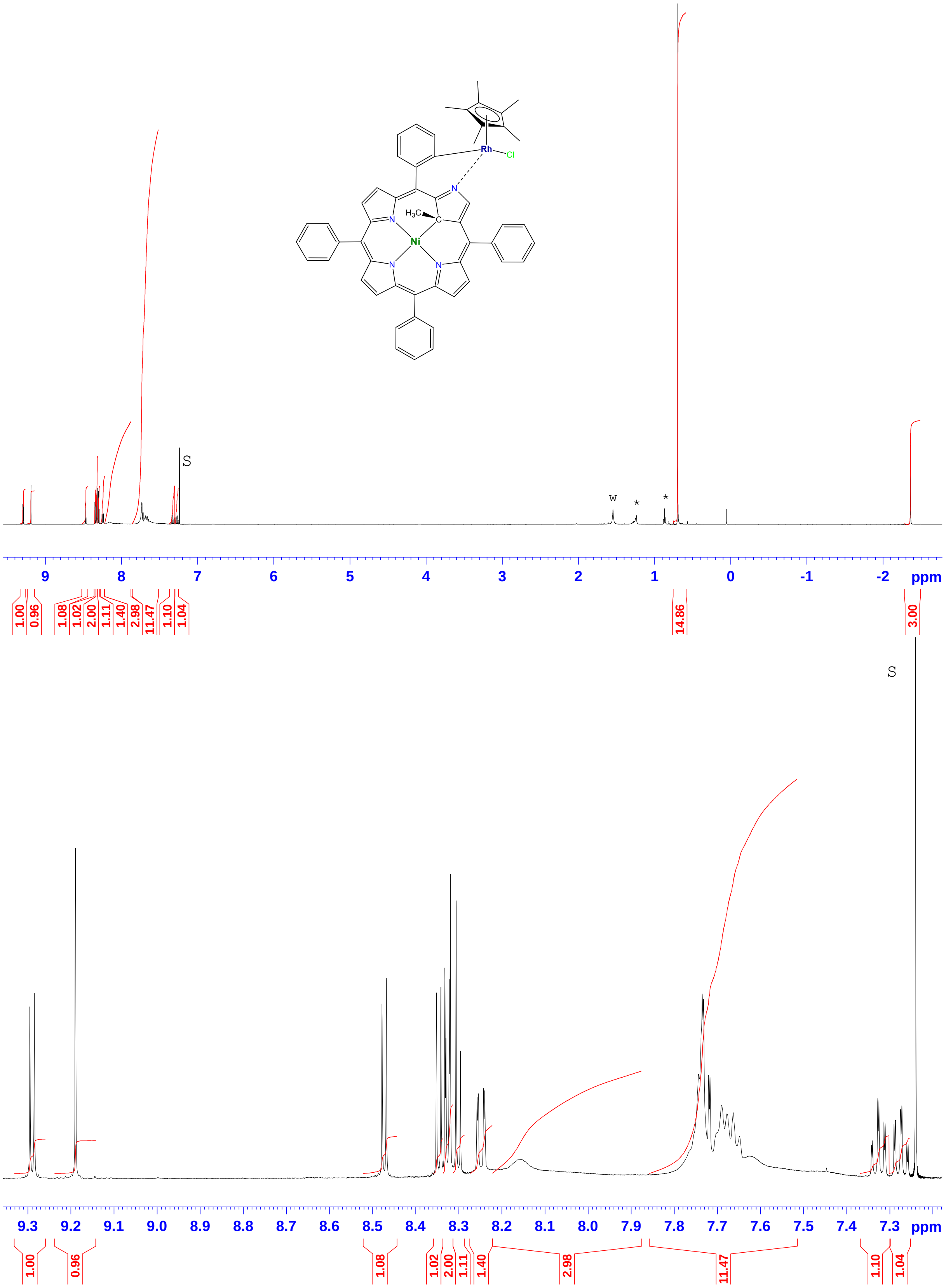

Figure S6. ^{1}H NMR spectrum (500 MHz, $CDCl_3$, 300 K) of **NiMeP**RhCp* (top) and expansion of the low-field region of this spectrum (bottom). s, residual $CHCl_3$ signal; w, dissolved water signal. The signal of impurities are marked with asterisks.

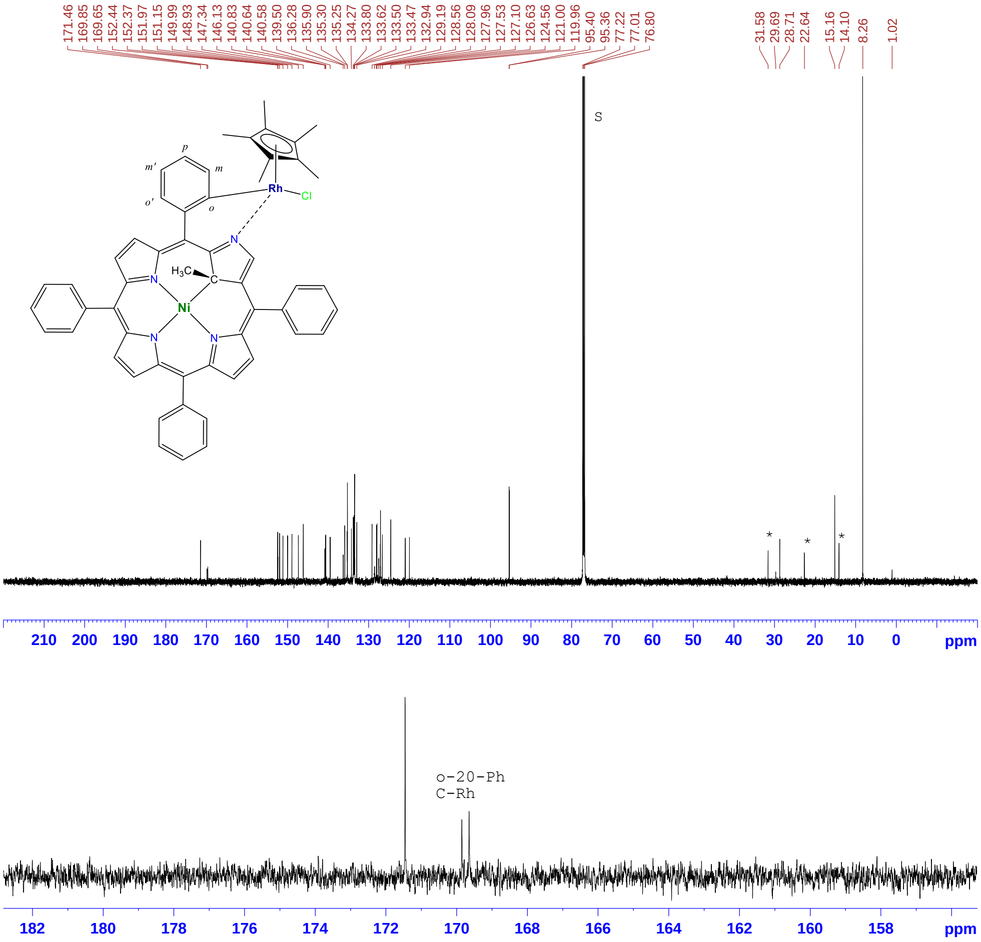

Figure S7. ^{13}C NMR spectrum (150 MHz, $CDCl_3$, 300 K) of **NiMeP**RhCp* (top) and expansion of the low-field region of this spectrum (bottom). s, $CDCl_3$ signal. The signal of impurities are marked with asterisks.

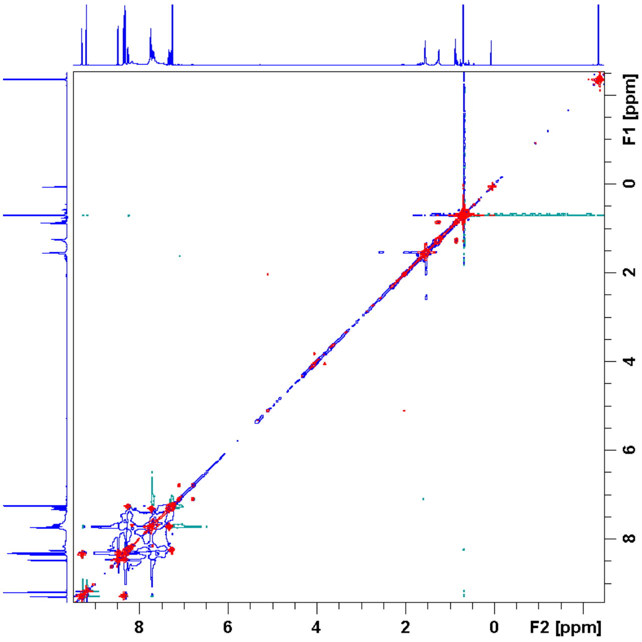

Figure S7A. Superimposed ^{1}H,^{1}H NOESY (blue and green crosspeaks) and ^{1}H,^{1}H COSY (red crosspeaks) spectra (600 MHz, $CDCl_3$, 300 K) of **NiMeP**RhCp*.

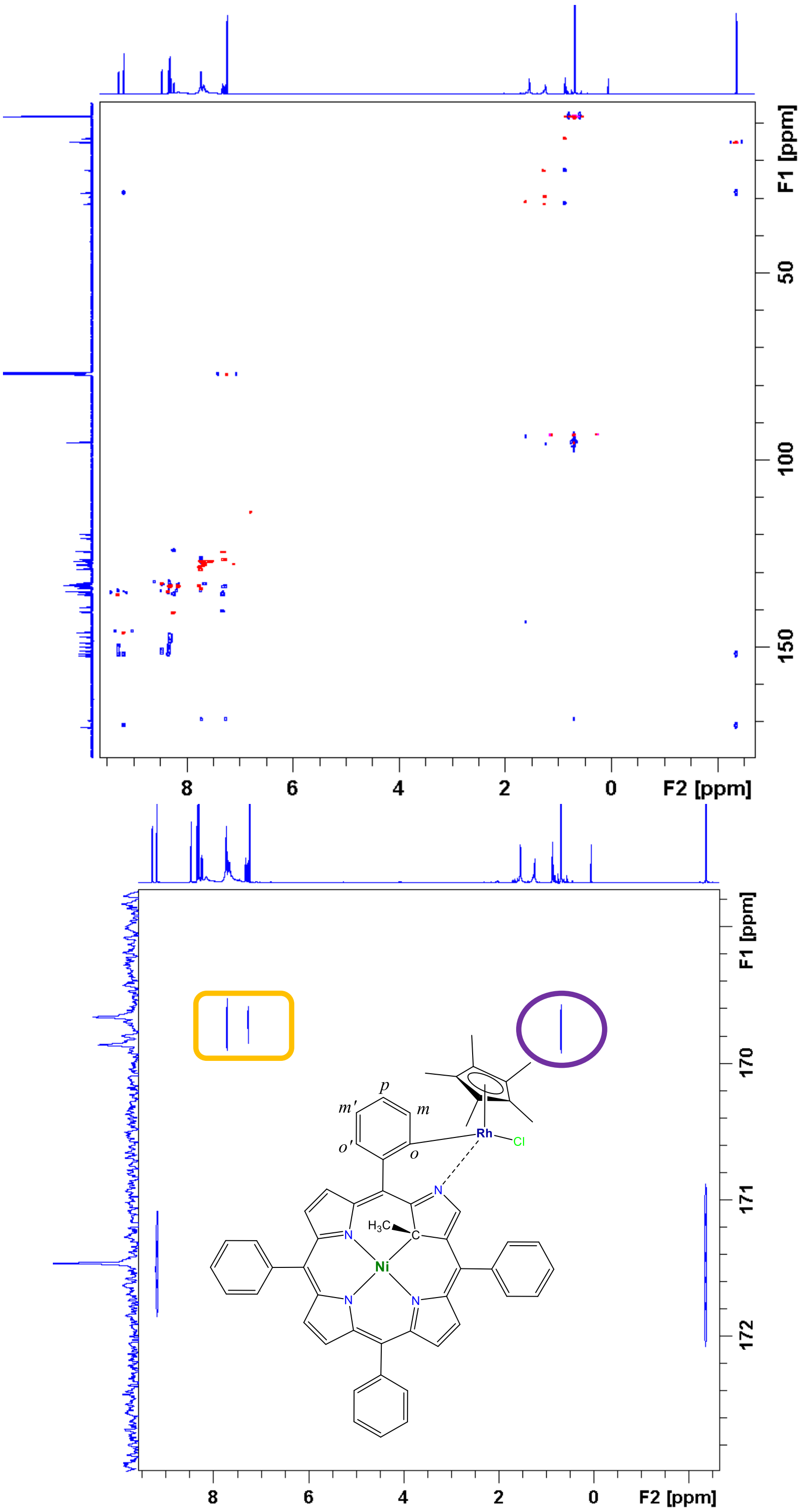

Figure S7B. Superimposed $^{13}C,^{1}H$ HMBC (blue crosspeaks) and $^{13}C,^{1}H$ HSQC (red crosspeaks) spectra (600/150 MHz, $CDCl_3$, 300 K) of **NiMePRhCp*** (top) and expansion of the selected region of the HMBC map (bottom) showing correlations of the rhodium(III)-coordinated *ortho*-C with *o'*- and *p*-20-Ph protons (golden frame) and with Cp* methyl protons (purple oval).

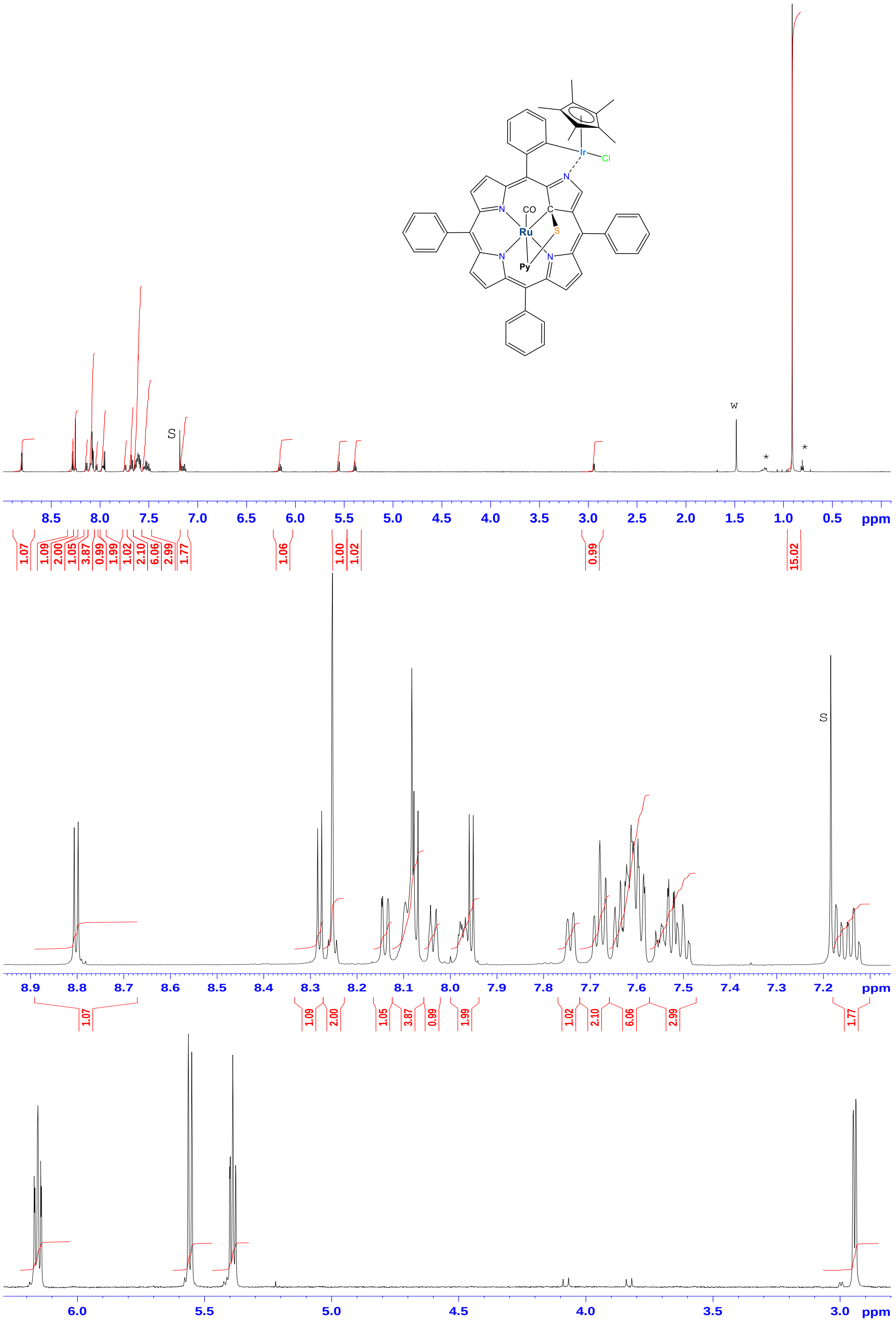

Figure S8. ^{1}H NMR spectrum (500 MHz, $CDCl_3$, 300 K) of **RuSPy**IrCp* (top), expansion of the low-field (middle), and mid-field (bottom) regions of this spectrum. s, residual $CHCl_3$ signal; w, dissolved water signal. The signal of impurities are marked with asterisks.

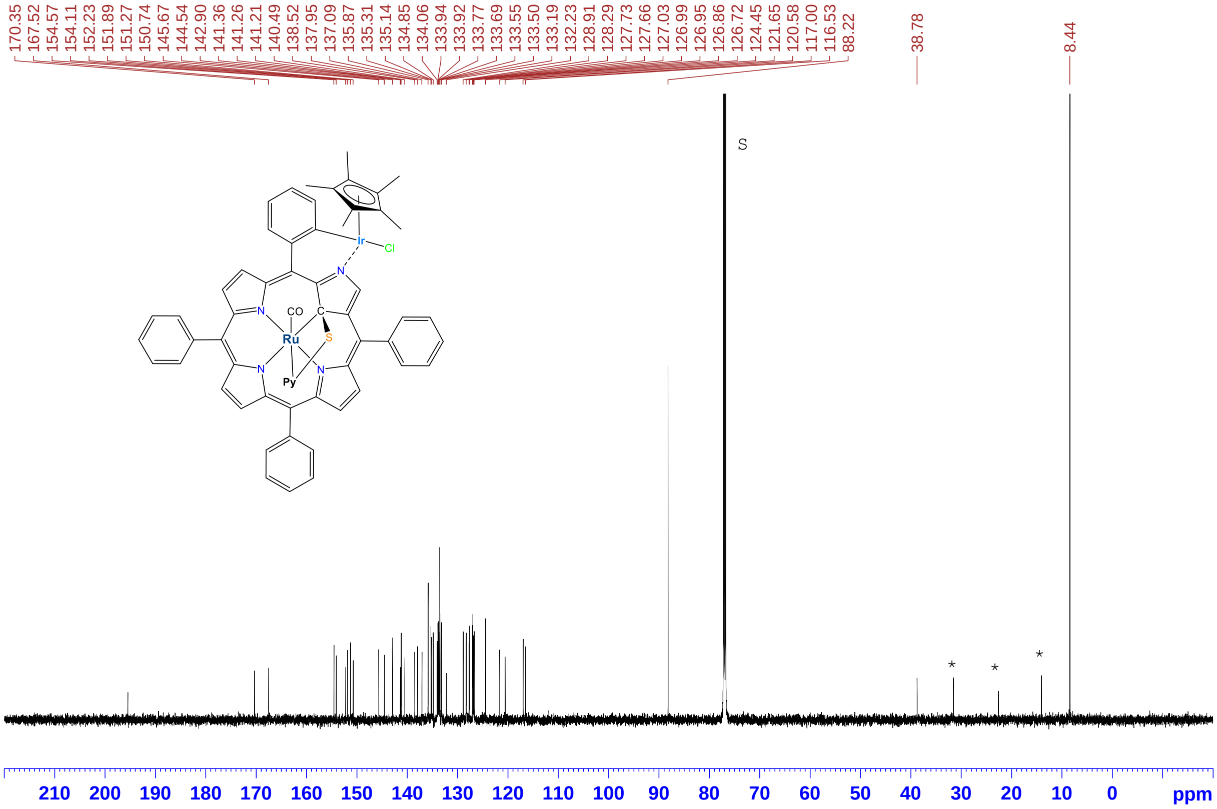

Figure S9. ^{13}C NMR spectrum (150 MHz, $CDCl_3$, 300 K) of **RuSPy**IrCp*. s, $CDCl_3$ signal. The signal of impurities are marked with asterisks.

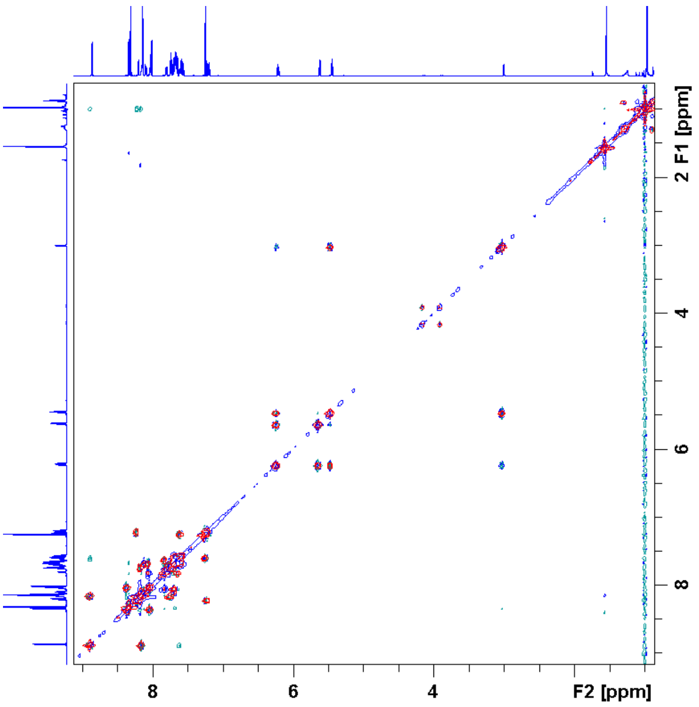

Figure S9A. Superimposed $^{1}H,^{1}H$ NOESY (blue and green crosspeaks) and $^{1}H,^{1}H$ COSY (red crosspeaks) spectra (600 MHz, $CDCl_3$, 300 K) of **RuSPy**IrCp*.

.

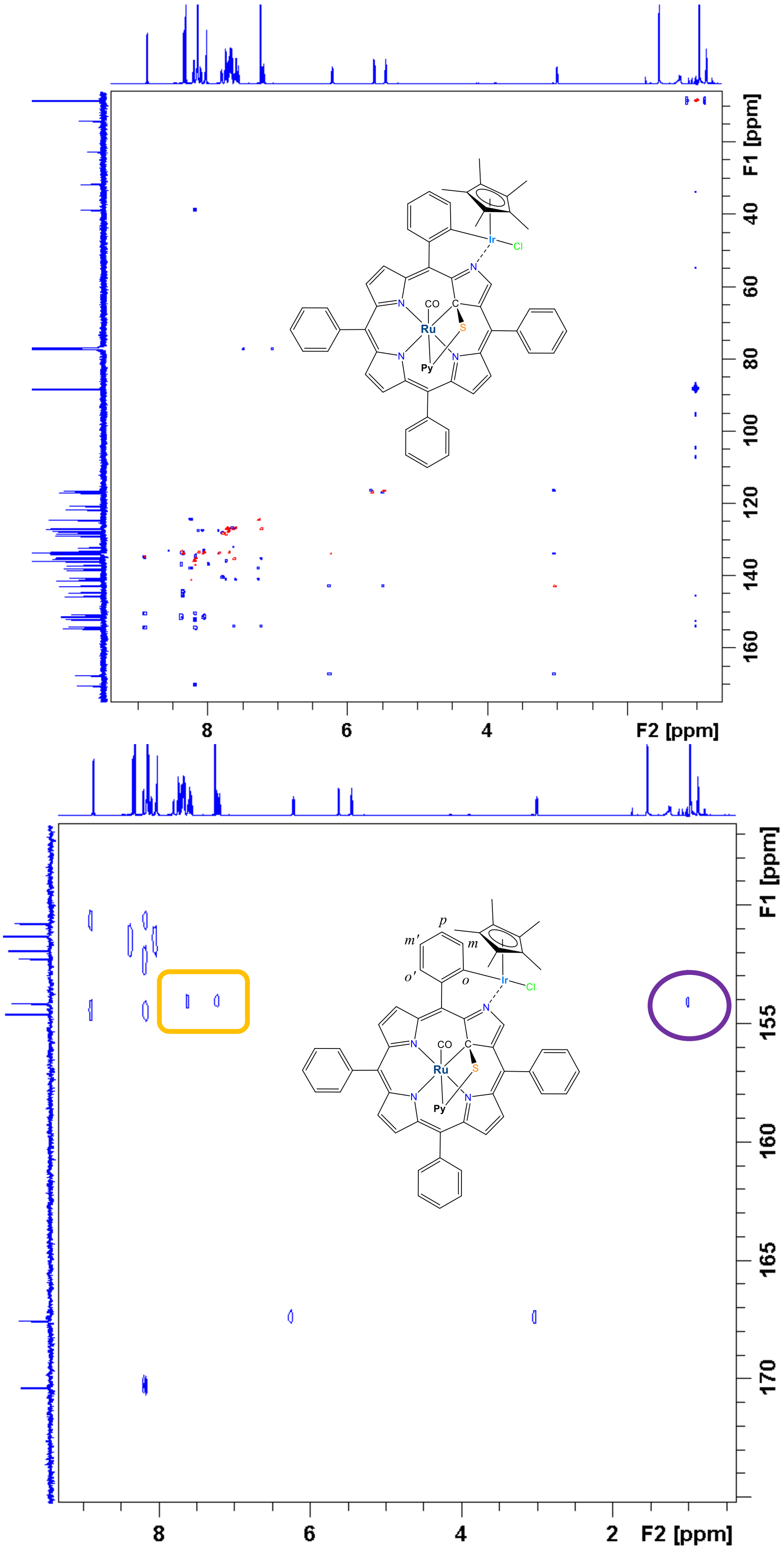

Figure S9B. Superimposed $^{13}C,^{1}H$ HMBC (blue crosspeaks) and $^{13}C,^{1}H$ HSQC (red crosspeaks) spectra (600/150 MHz, $CDCl_3$, 300 K) of **RuSPy**IrCp* (top) and expansion of the selected region of the HMBC map (bottom) showing correlations of the iridium(III)-coordinated *ortho*-C with *o'*- and *p*-20-Ph protons (golden frame) and with Cp* methyl protons (purple oval).

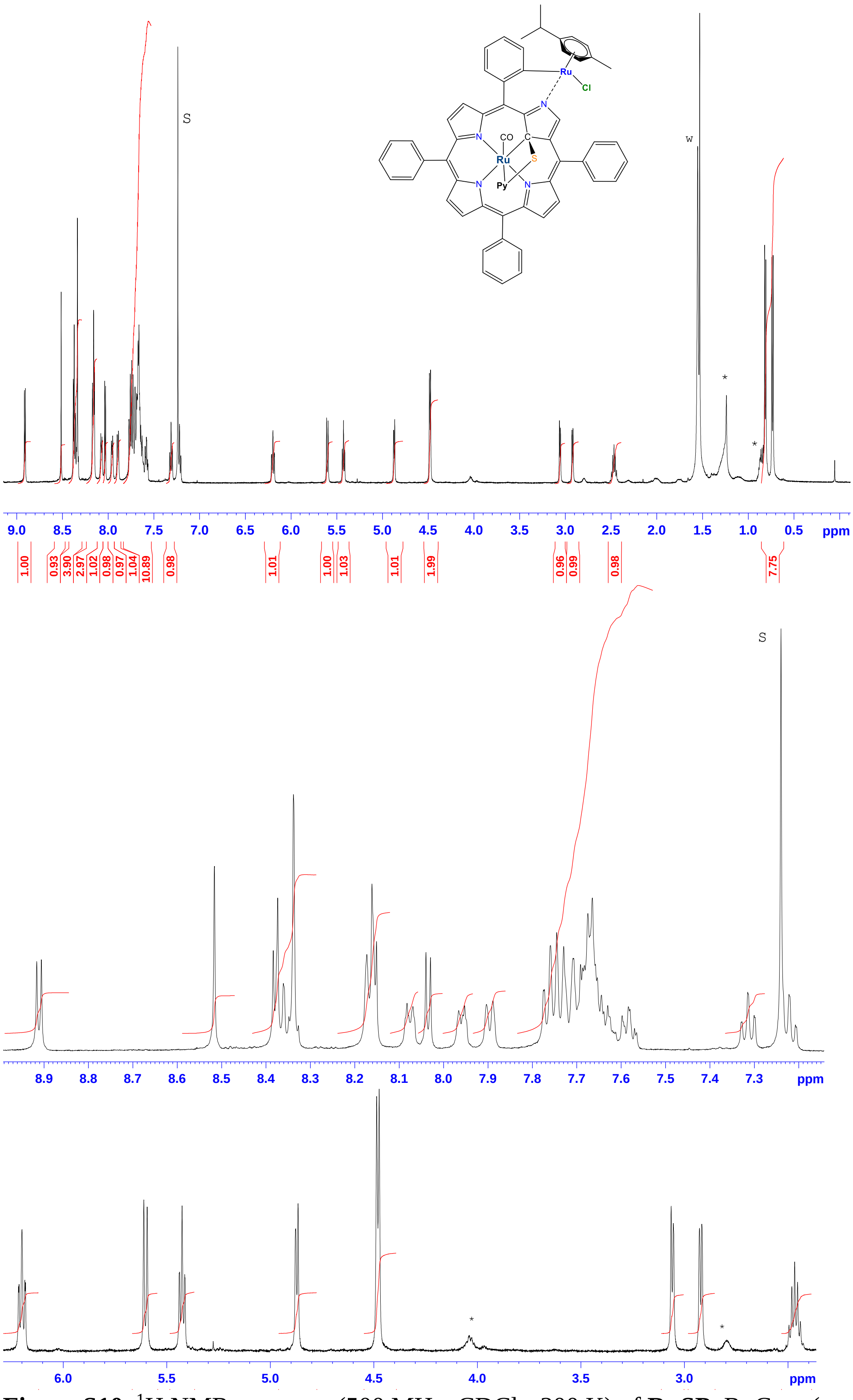

Figure S10. ^{1}H NMR spectrum (500 MHz, $CDCl_3$, 300 K) of **RuSPy**RuCym (top), expansion of the low-field (middle), and mid-field (bottom) regions of this spectrum. s, residual $CHCl_3$ signal; w, dissolved water signal. The signal of impurities are marked with asterisks.

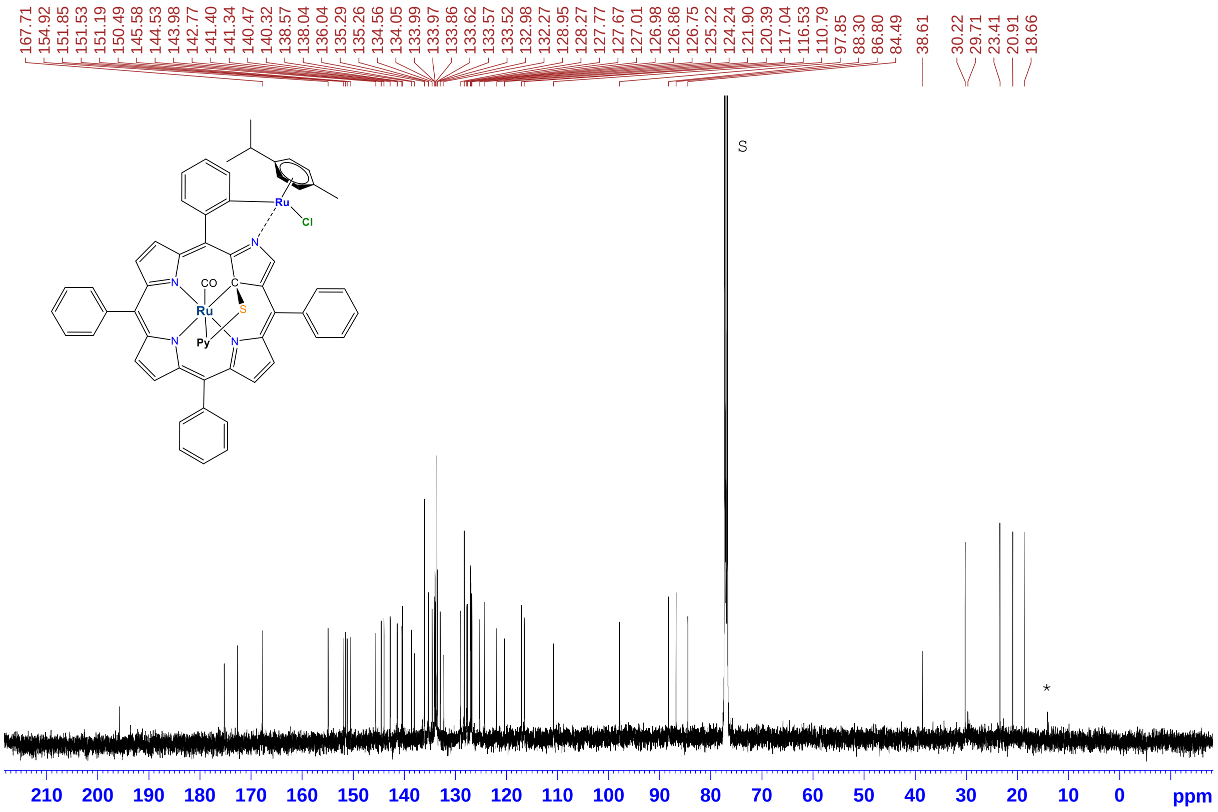

Figure S11. ^{13}C NMR spectrum (150 MHz, $CDCl_3$, 300 K) of **RuSPy**RuCym. s, $CDCl_3$ signal. The signal of impurities are marked with asterisks.

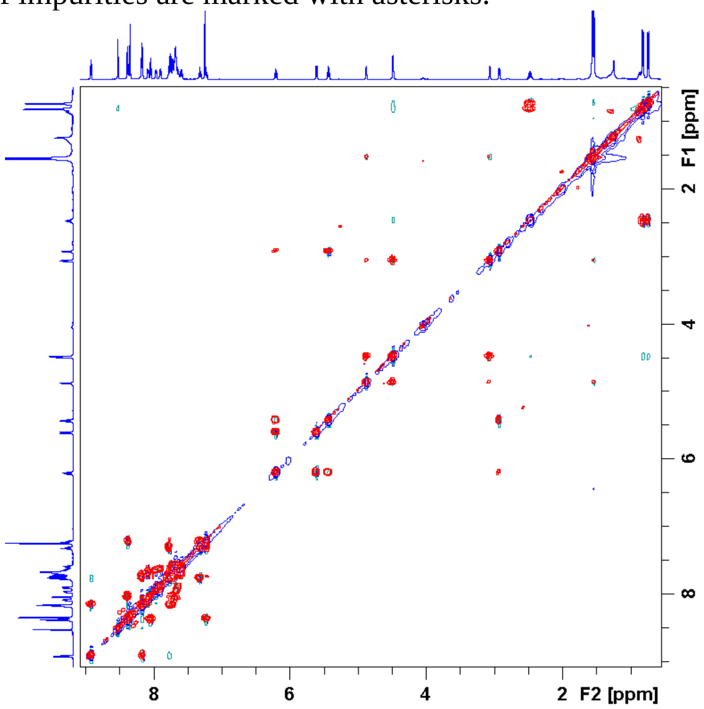

Figure S11A. Superimposed ^{1}H,^{1}H NOESY (blue and green crosspeaks) and ^{1}H,^{1}H COSY (red crosspeaks) spectra (600 MHz, $CDCl_3$, 300 K) of **RuSPy**RuCym.

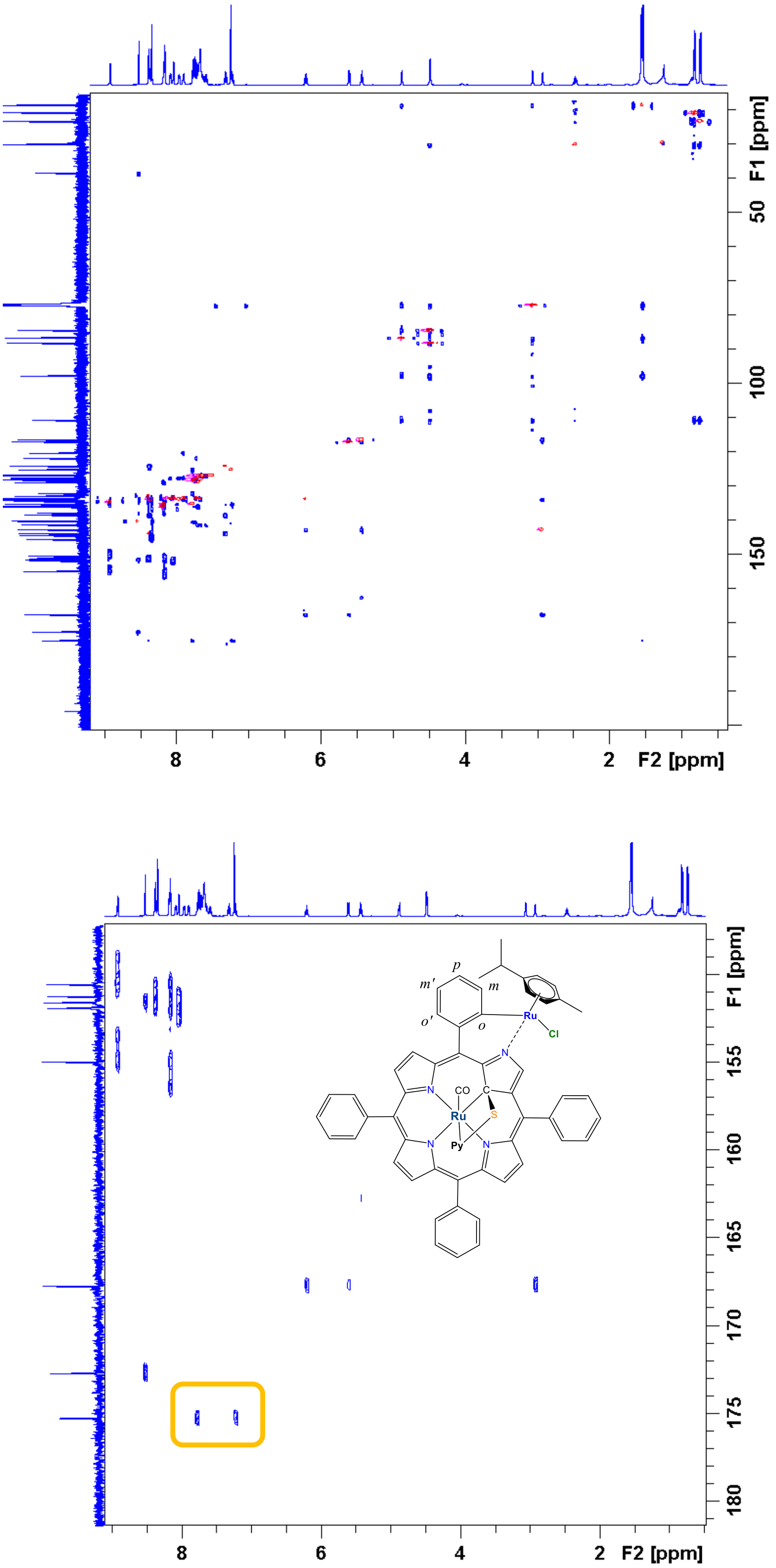

Figure S11B. Superimposed ^{13}C,^{1}H HMBC (blue crosspeaks) and ^{13}C,^{1}H HSQC (red crosspeaks) spectra (600/150 MHz, $CDCl_3$, 300 K) of **RuSPy**RuCym (top) and expansion of the selected region of the HMBC map (bottom) showing correlations of the ruthenium(II)-coordinated *ortho*-C with *o'*- and *p*-20-Ph protons (golden frame).

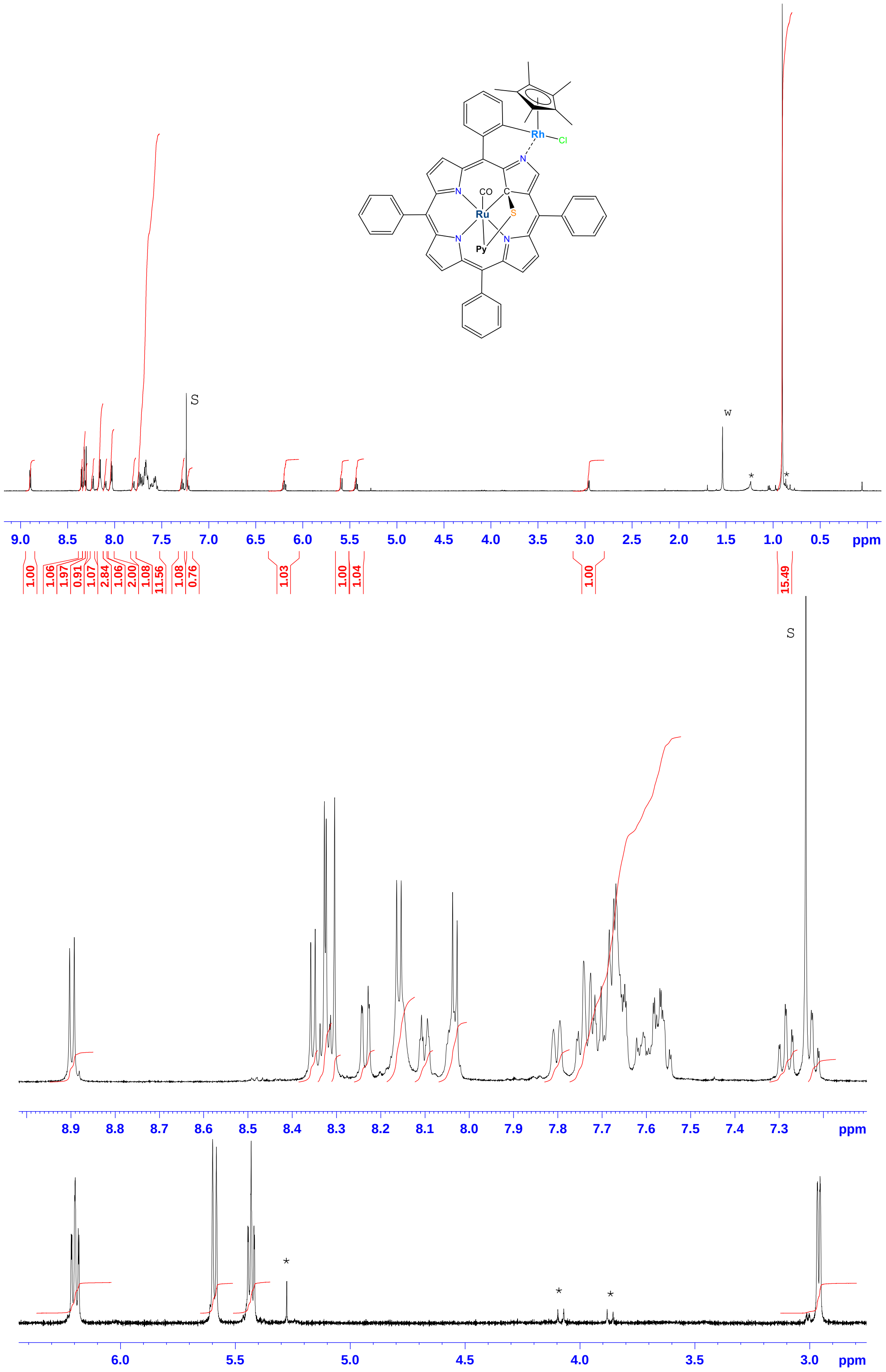

Figure S12. ^{1}H NMR spectrum (500 MHz, $CDCl_3$, 300 K) of **RuSPy**RhCp* (top), expansion of the low-field (middle), and mid-field (bottom) regions of this spectrum. s, residual $CHCl_3$ signal; w, dissolved water signal. The signal of impurities are marked with asterisks

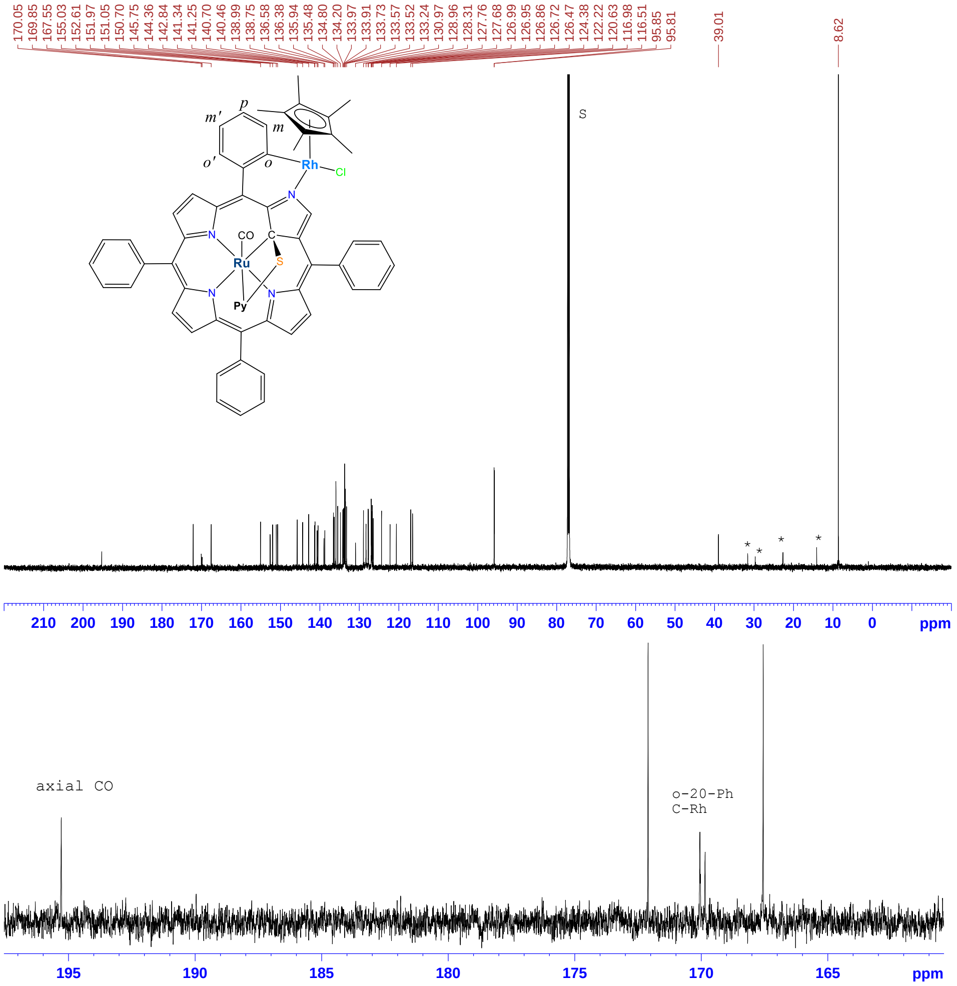

Figure S13. ^{13}C NMR spectrum (150 MHz, $CDCl_3$, 300 K) of **RuSPy**RhCp* (top) and expansion of the low-field region of this spectrum (bottom). s, $CDCl_3$ signal. The signal of impurities are marked with asterisks.

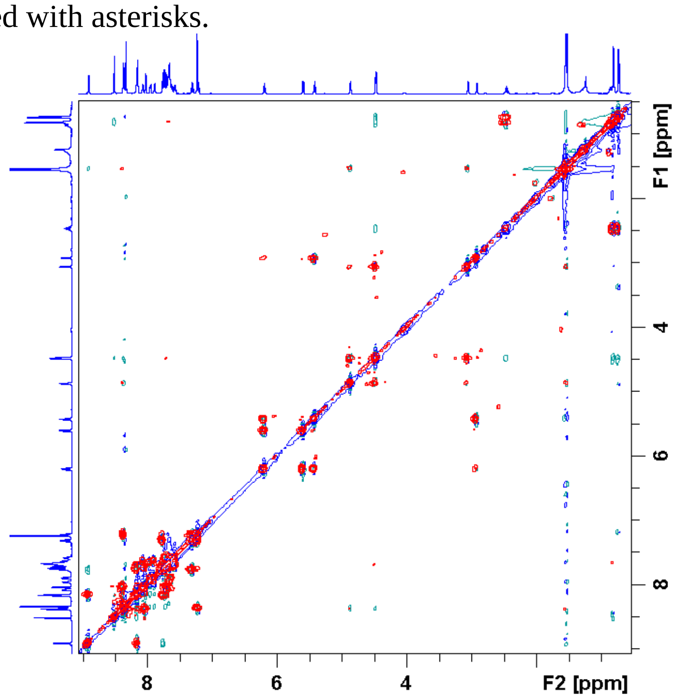

Figure S13A. Superimposed ^{1}H,^{1}H NOESY (blue and green crosspeaks) and ^{1}H,^{1}H COSY (red crosspeaks) spectra (600 MHz, $CDCl_3$, 300 K) of **RuSPy**RhCp*.

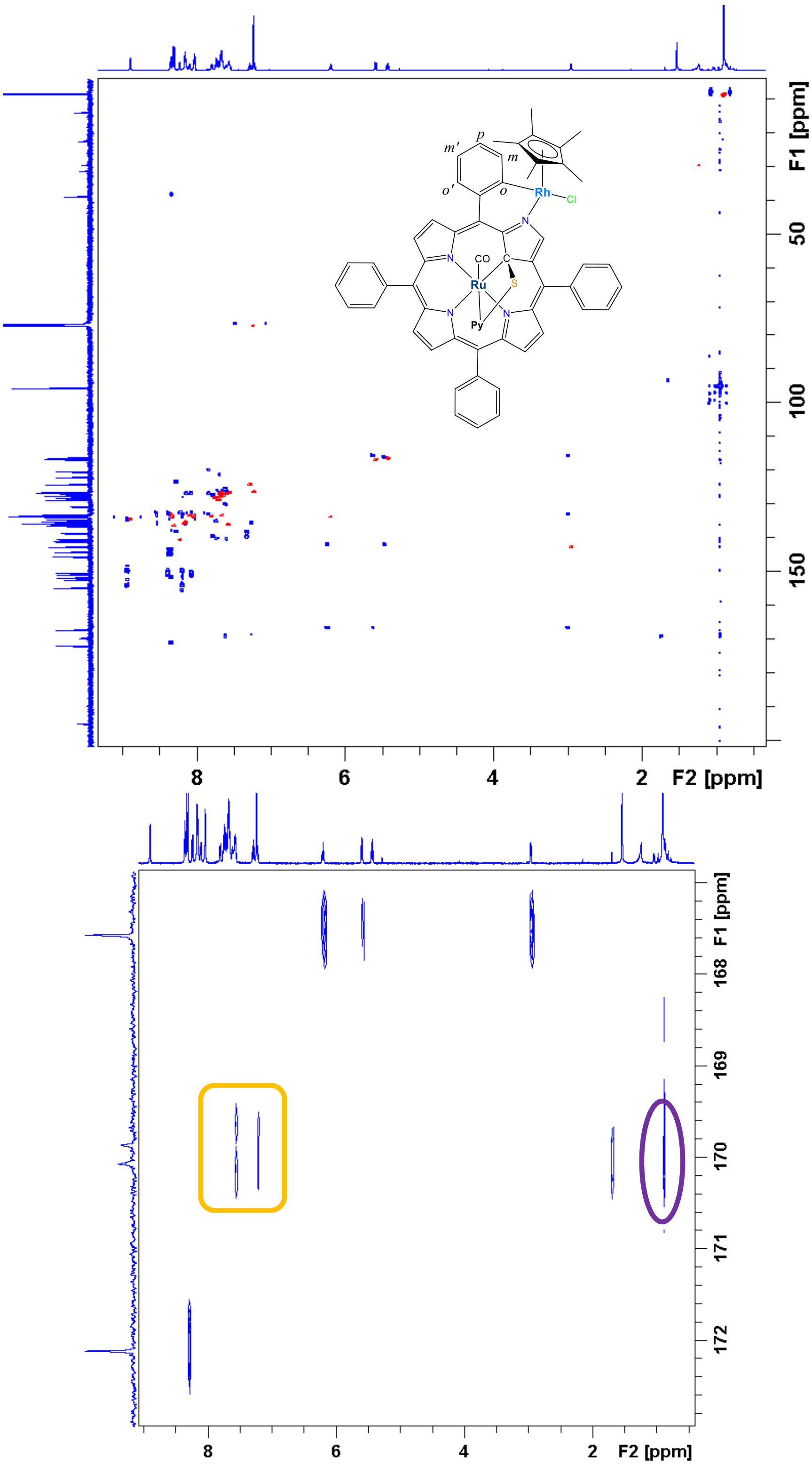

Figure S13B. Superimposed $^{13}C,^{1}H$ HMBC (blue crosspeaks) and $^{13}C,^{1}H$ HSQC (red crosspeaks) spectra (600/150 MHz, $CDCl_3$, 300 K) of **RuSPy**RhCp* (top) and expansion of the selected region of the HMBC map (bottom) showing correlations of the rhodium(III)-coordinated *ortho*-C with *o'*- and *p*-20-Ph protons (golden frame) and with Cp* methyl protons (purple oval).

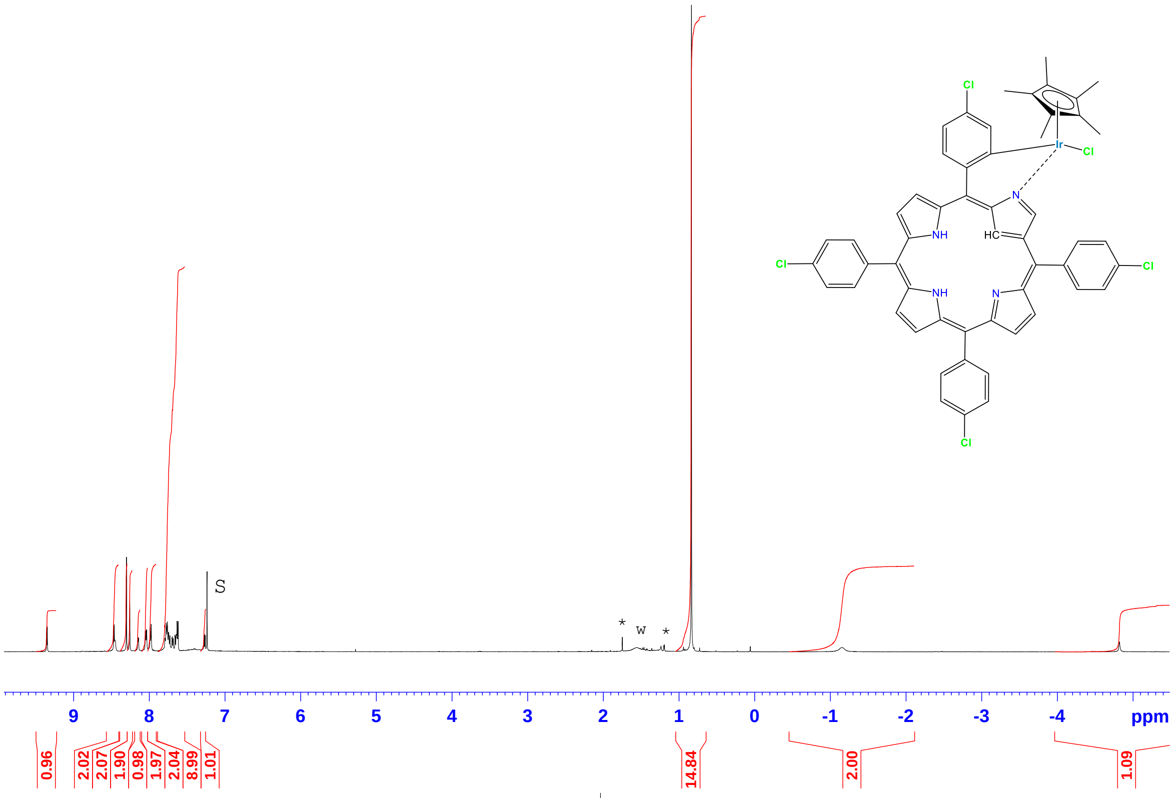

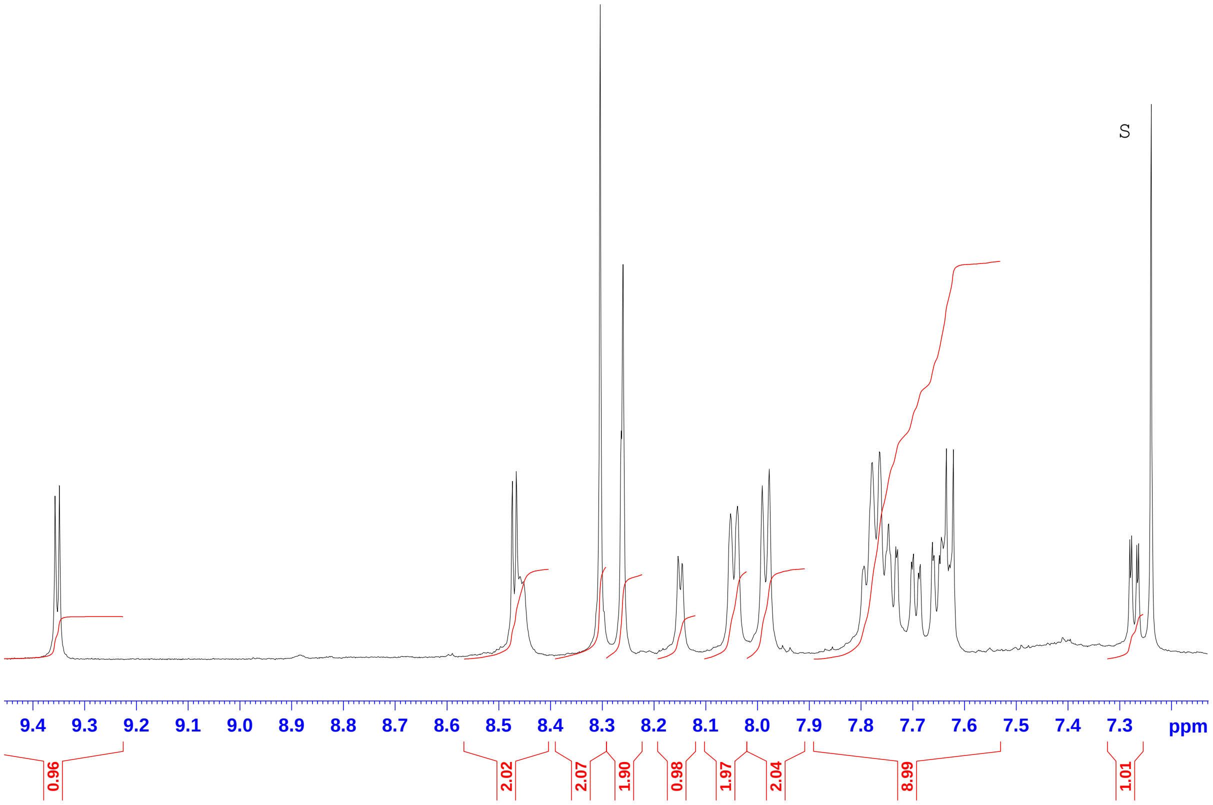

Figure S14. ^{1}H NMR spectrum (500 MHz, $CDCl_3$, 300 K) of **ClNCP**IrCp* (top) and expansion of the low-field region of this spectrum (bottom). s, residual $CHCl_3$ signal; w, dissolved water signal. The signal of impurities are marked with asterisks.

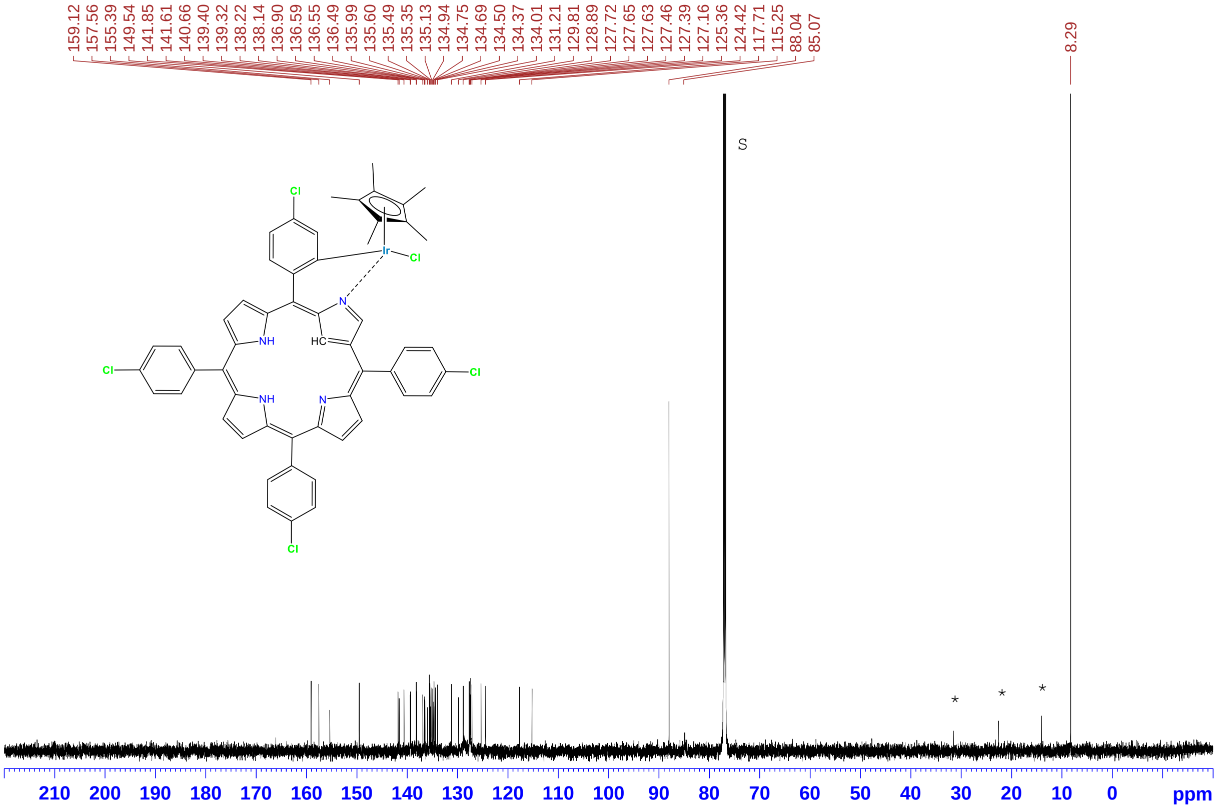

Figure S15. ^{13}C NMR spectrum (150 MHz, $CDCl_3$, 300 K) of **ClNCP**IrCp*. s, $CDCl_3$ signal. The signal of impurities are marked with asterisks.

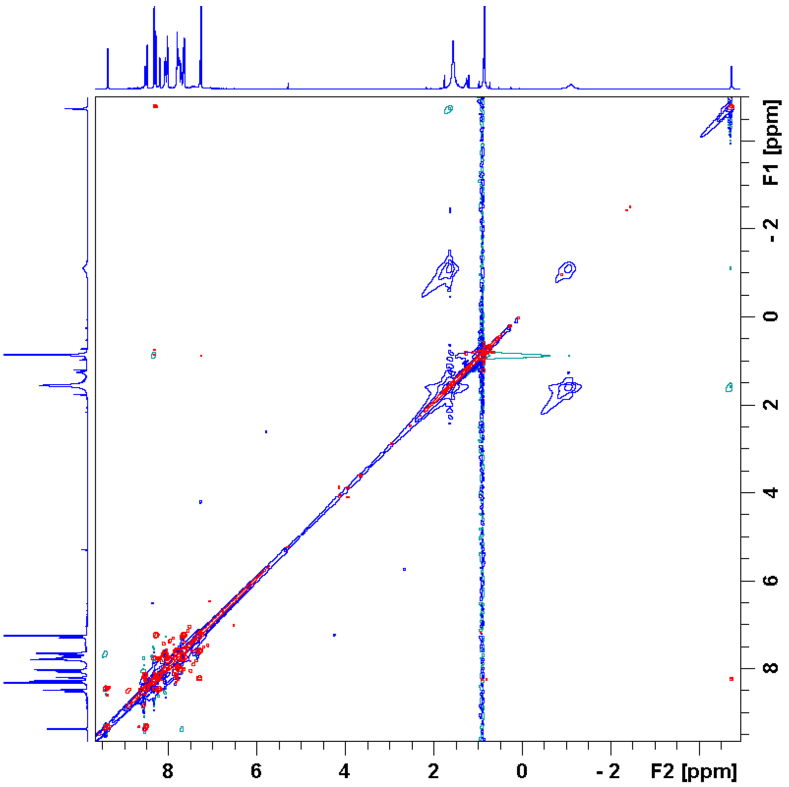

Figure S15A. Superimposed $^{1}H,^{1}H$ NOESY (blue and green crosspeaks) and $^{1}H,^{1}H$ COSY (red crosspeaks) spectra (600 MHz, $CDCl_3$, 300 K) of **ClNCP**IrCp*.

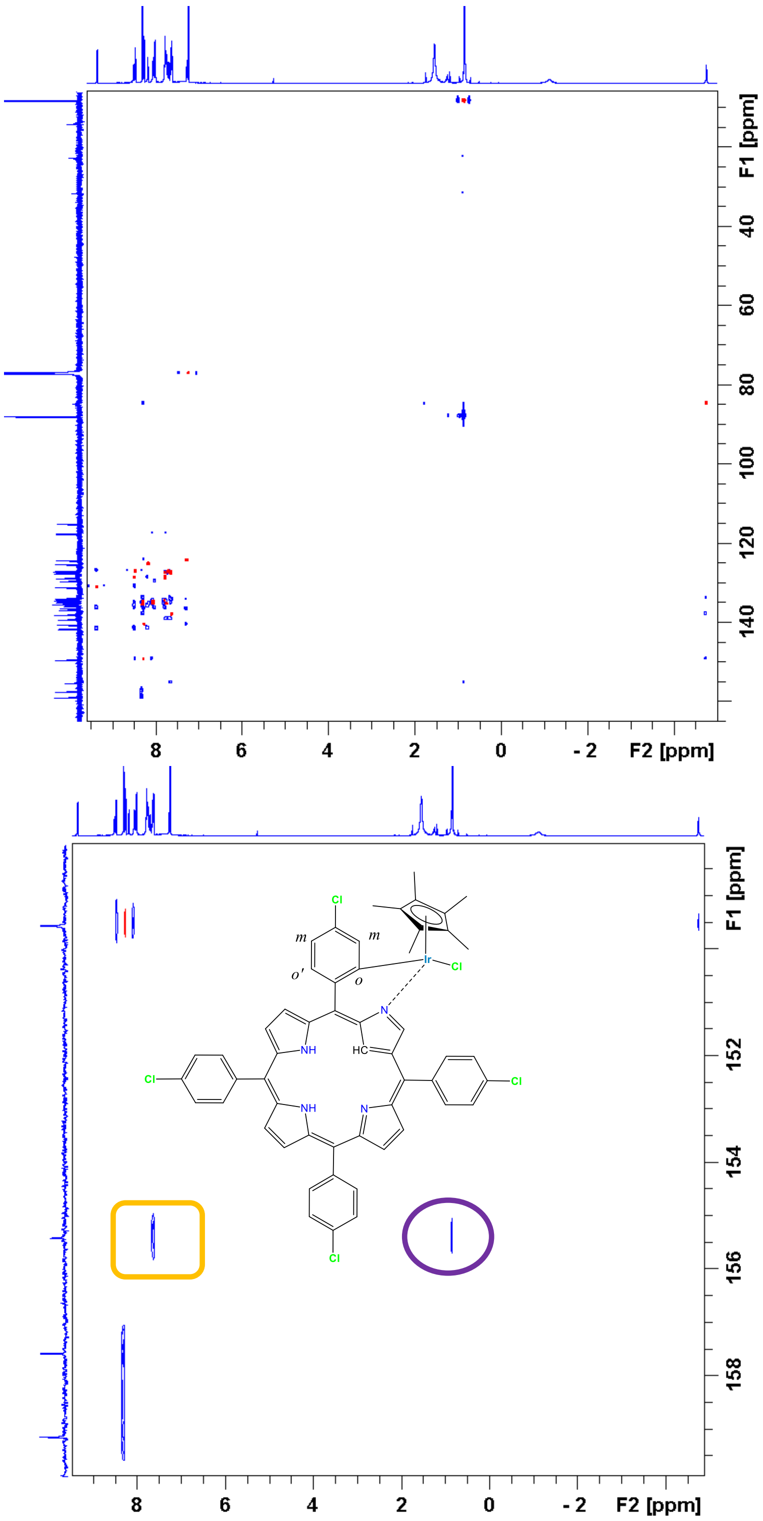

Figure S15B. Superimposed $^{13}C,^{1}H$ HMBC (blue crosspeaks) and $^{13}C,^{1}H$ HSQC (red crosspeaks) spectra (600/150 MHz, $CDCl_3$, 300 K) of **ClNCP**IrCp* (top) and expansion of the selected region of the HMBC/HSQC maps (bottom) showing correlations of the iridium(III)-coordinated *ortho*-C with *o*'-20-Ph protons (golden frame) and with Cp* methyl protons (purple oval).

High-resolution mass spectra

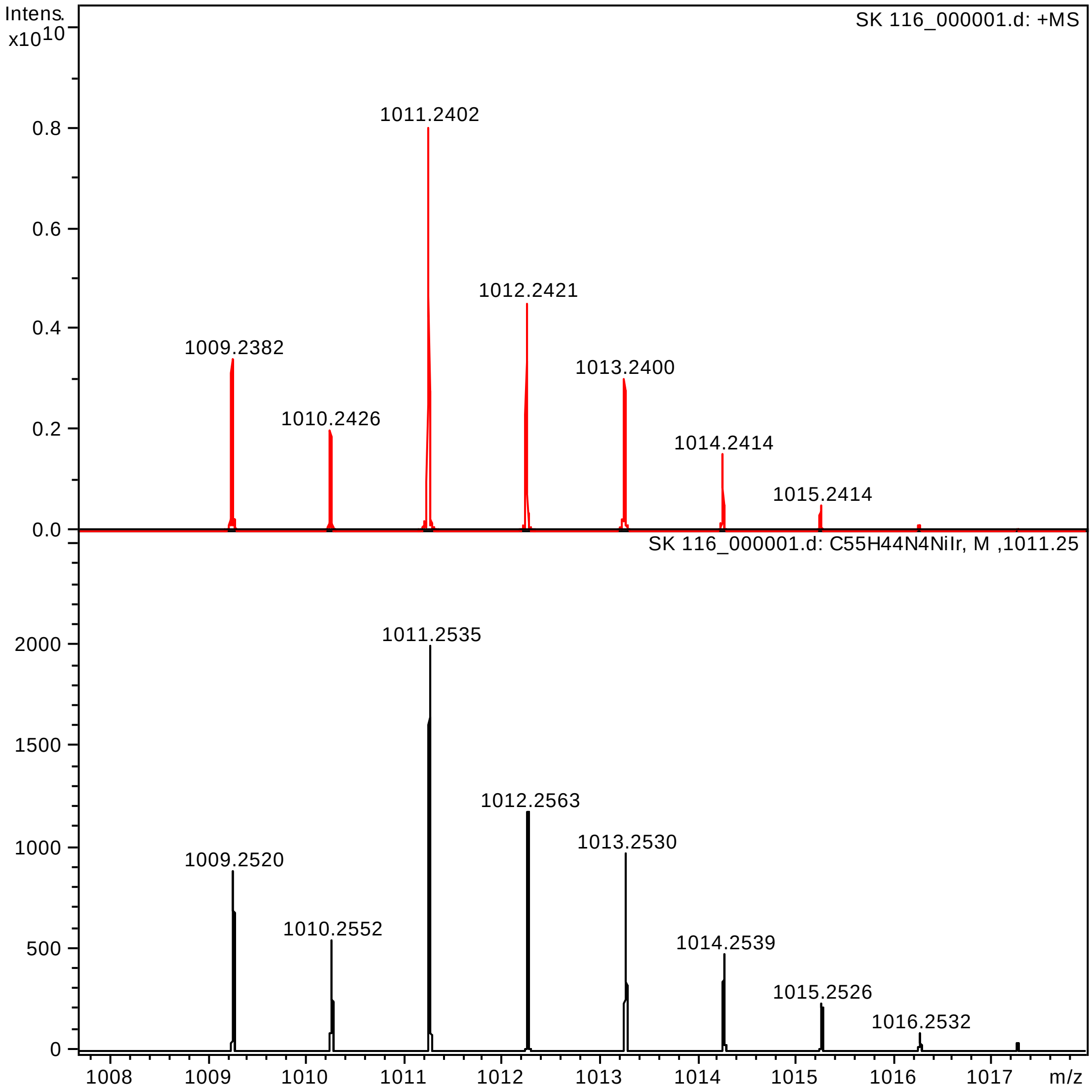

Figure S16. High-resolution mass spectra (ESI +) of **NiMeP**IrCp*: top, experimental; bottom, simulated for $[M{-}Cl]^+$.

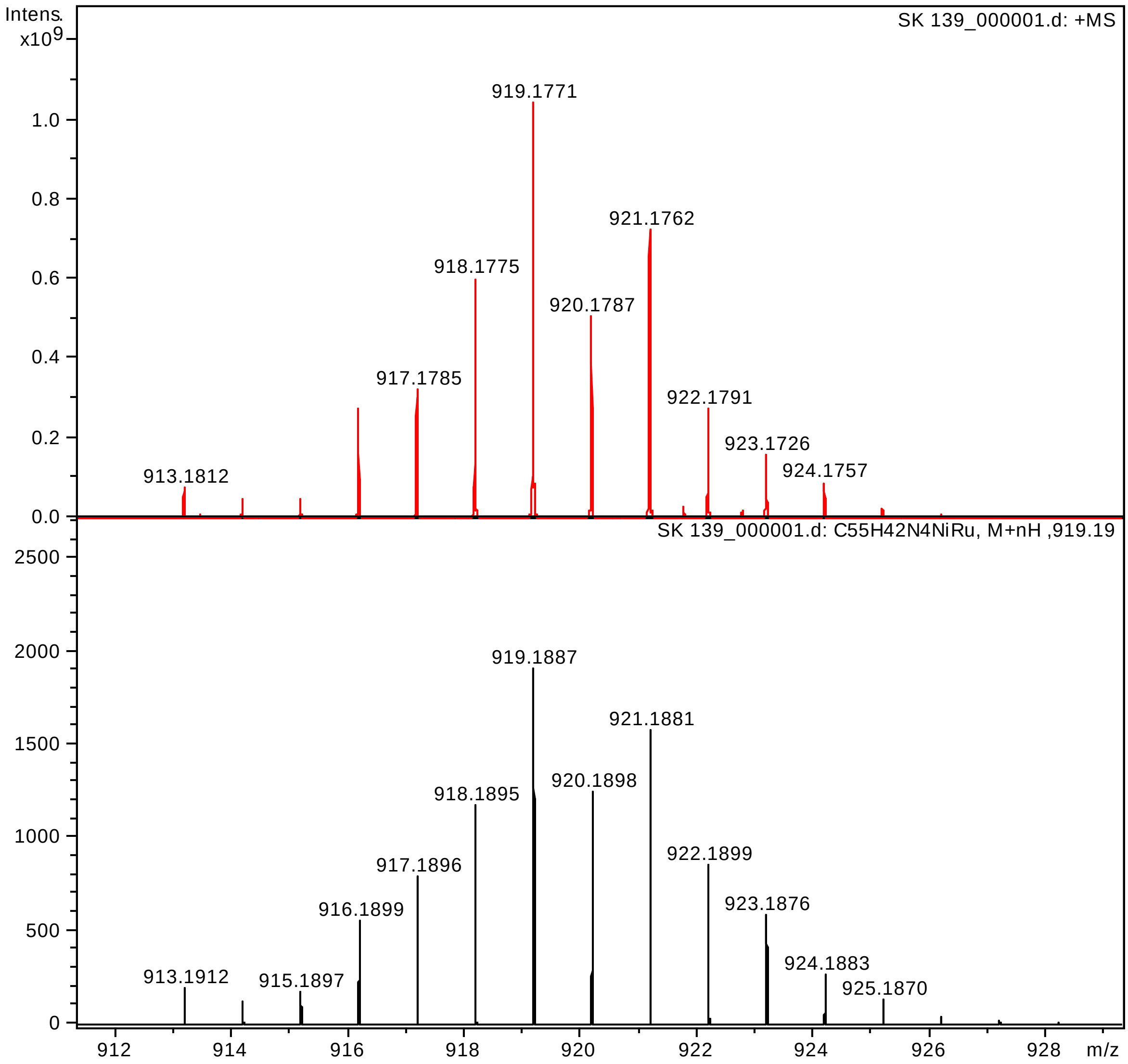

Figure S17. High-resolution mass spectra (ESI +) of **NiMeP**RuCym: top, experimental; bottom, simulated for $[M–Cl]^+$.

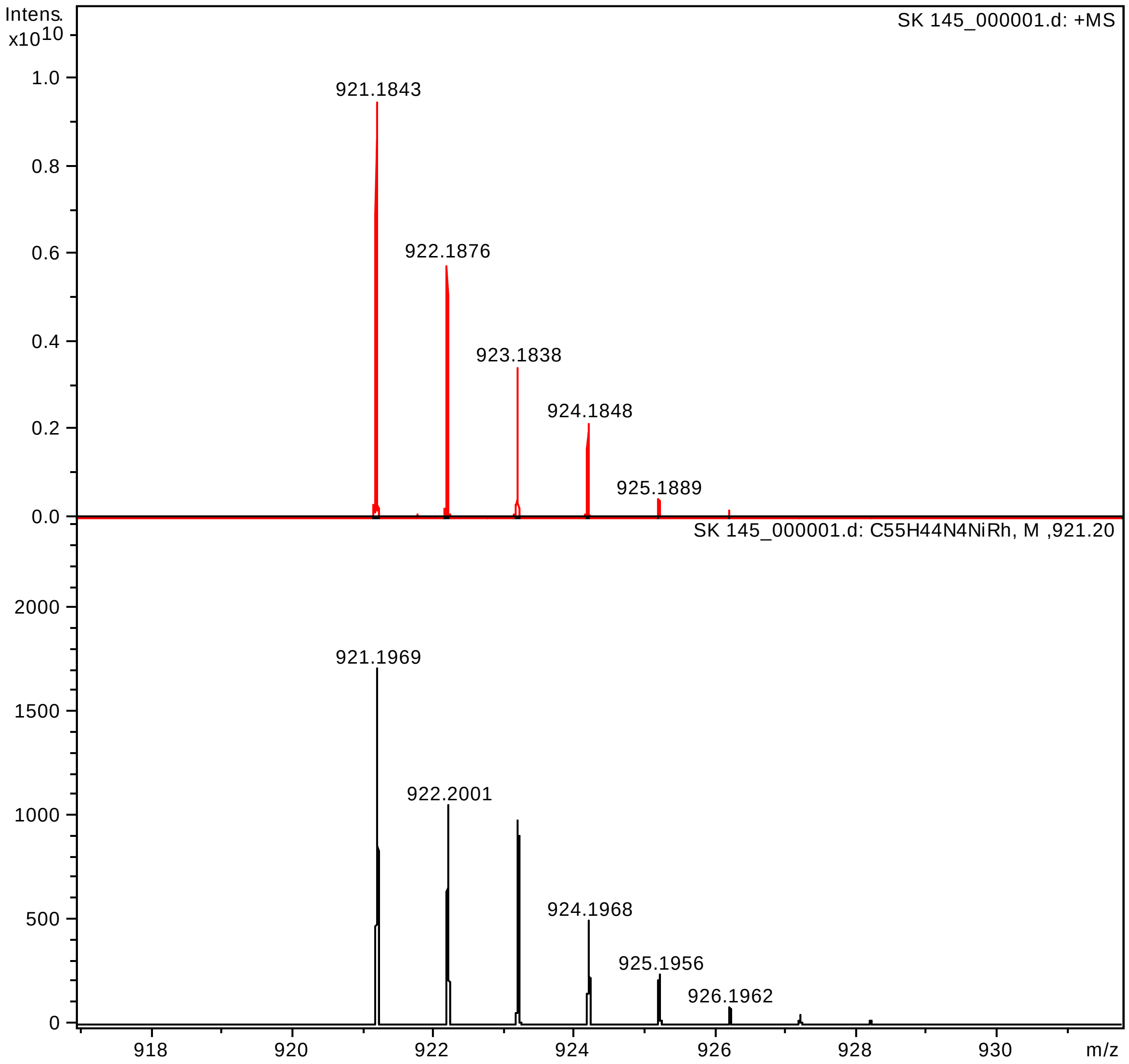

Figure S18. High-resolution mass spectra (ESI +) of **NiMeP**RhCp*: top, experimental; bottom, simulated for $[M–Cl]^+$.

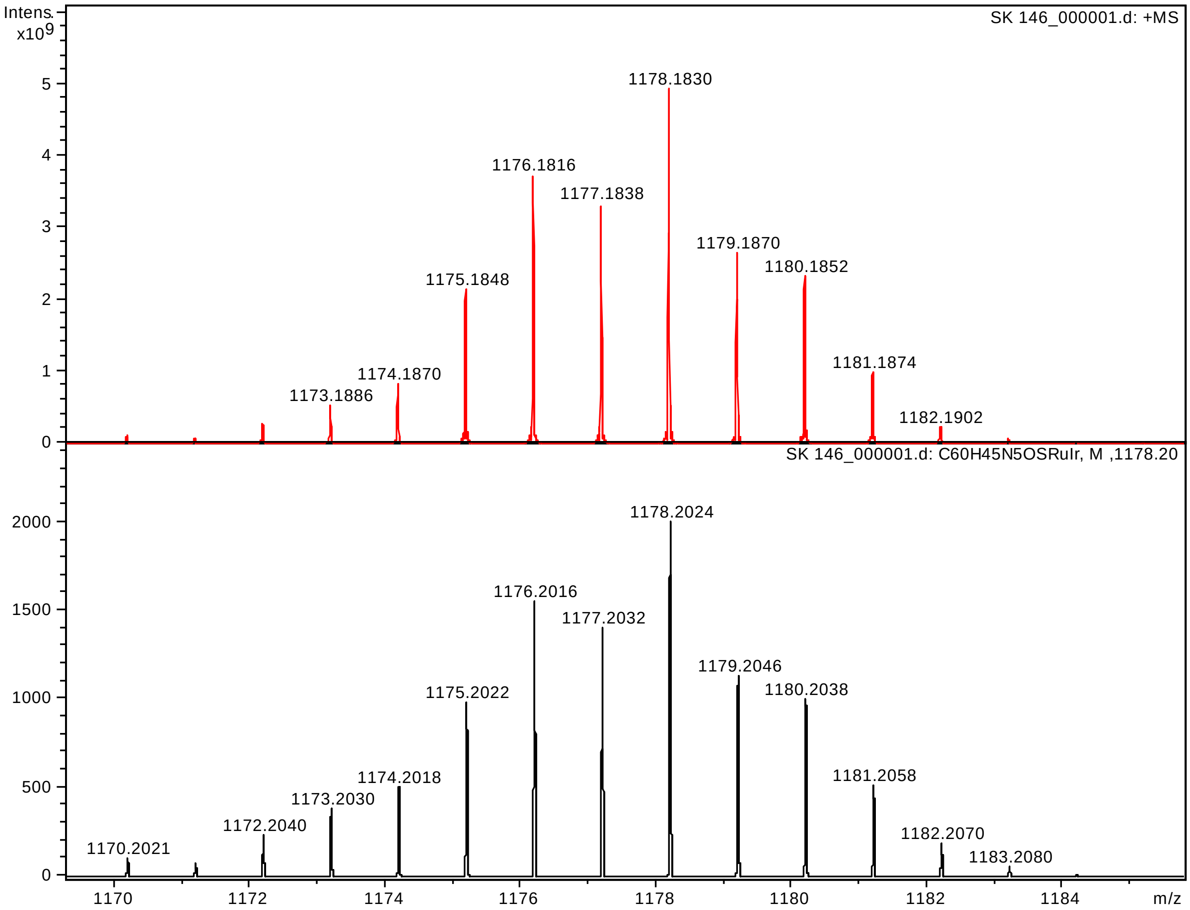

Figure S19. High-resolution mass spectra (ESI +) of **RuSPy**IrCp*: top, experimental; bottom, simulated for $[M–Cl]^+$.

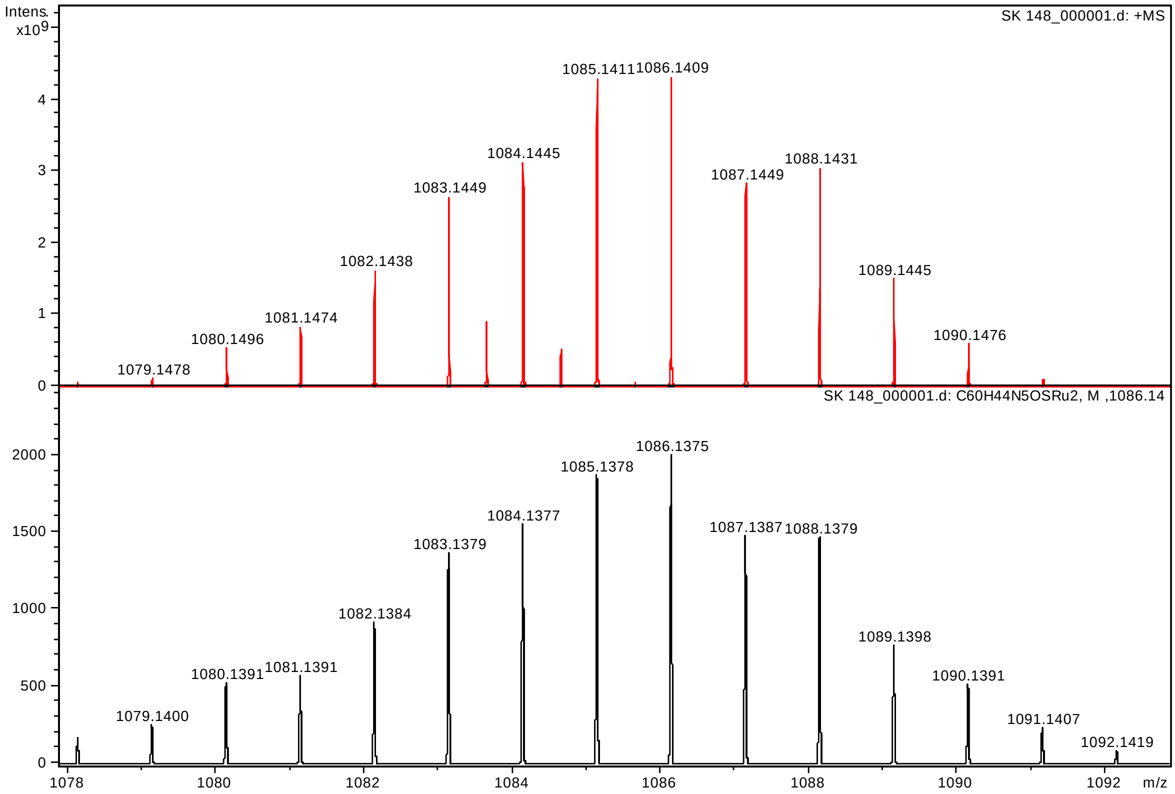

Figure S20A. High-resolution mass spectra (ESI +) of **RuSPy**RuCym: top, experimental; bottom, simulated for [M–Cl]$^+$.

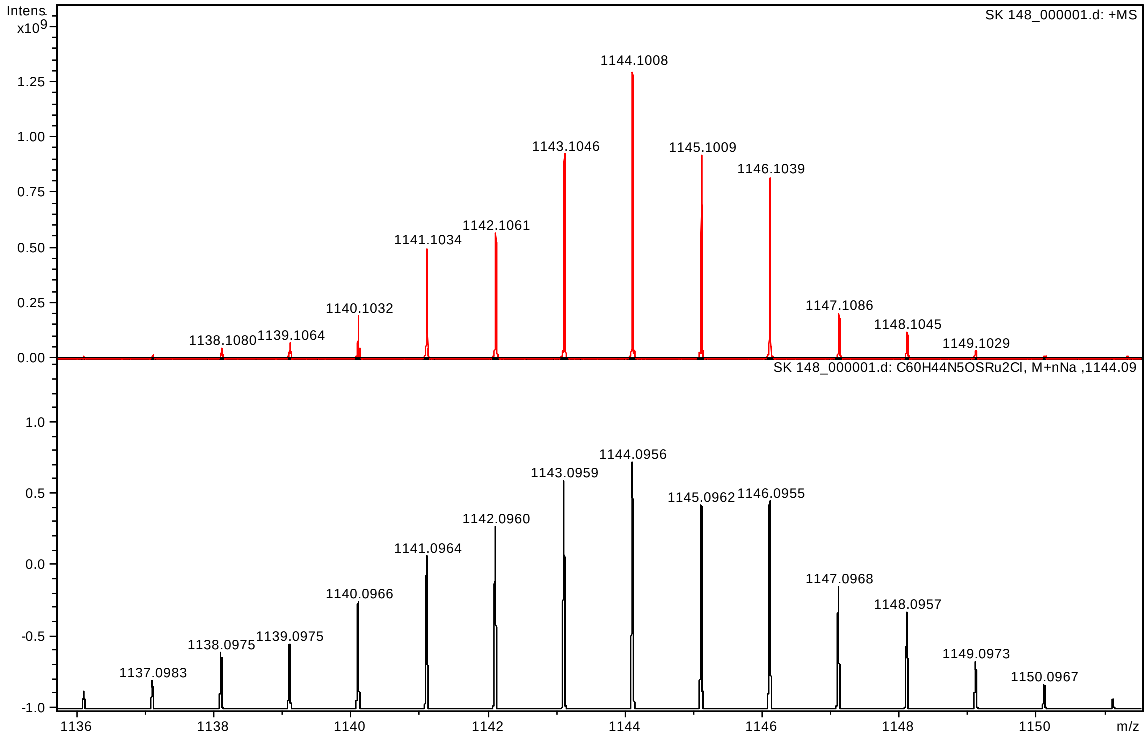

Figure S20B. High-resolution mass spectra (ESI +) of **RuSPy**RuCym: top, experimental; bottom, simulated for [M+Na]$^+$.

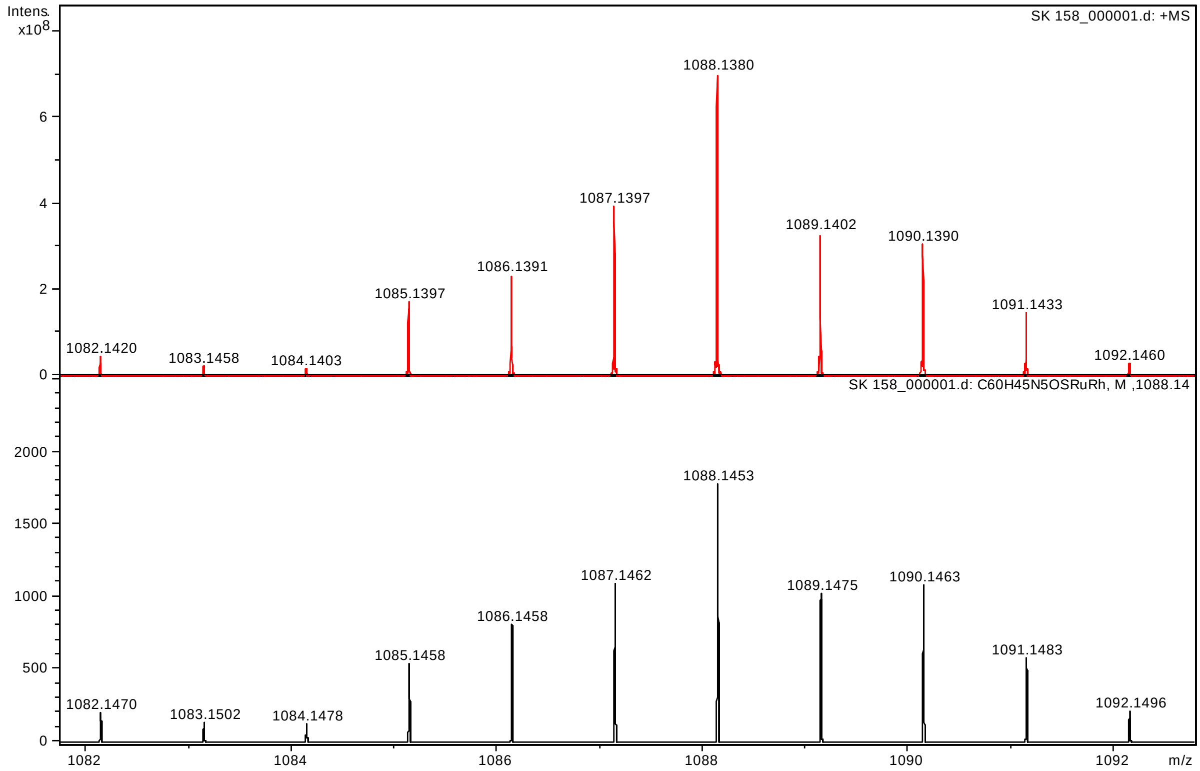

Figure S21. High-resolution mass spectra (ESI +) of **RuSPy**RhCp*: top, experimental; bottom, simulated for [M–Cl]$^+$.

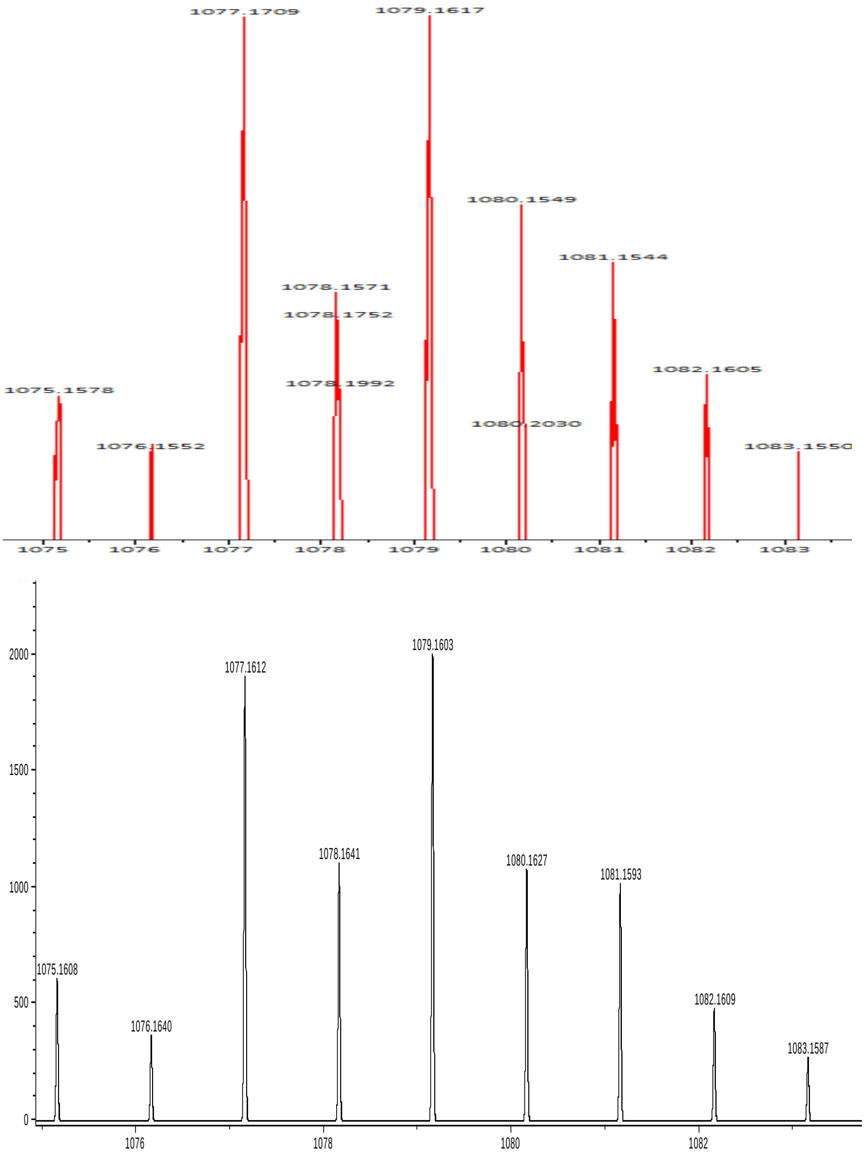

Figure S22. High-resolution mass spectra (ESI +) of **4ClNCP**IrCp*: top, experimental; bottom, simulated for $[M–Cl]^+$.

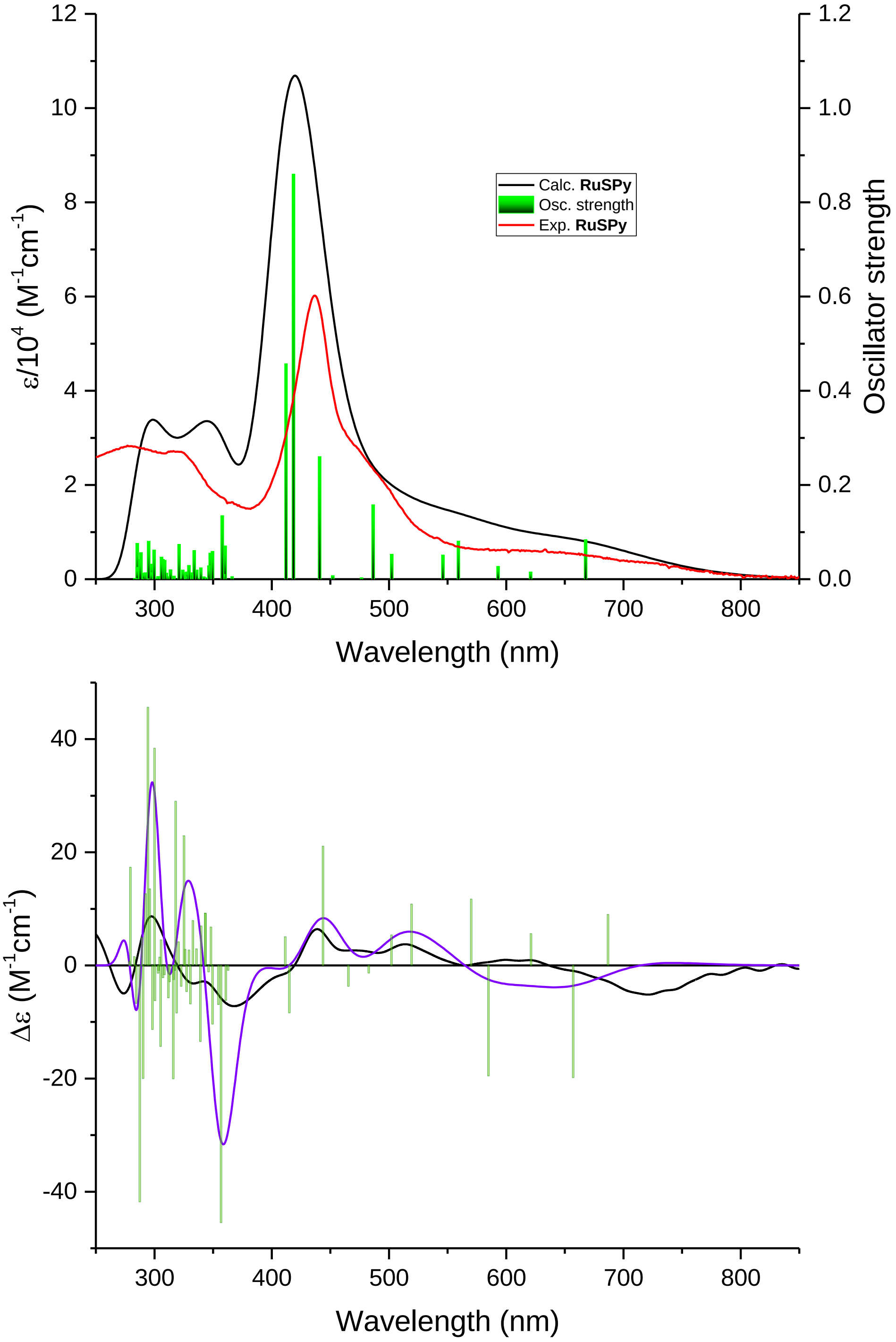

Figure S23. Top: experimental (DCM, red trace) and TD DFT-calculated (black trace) absorbance spectra of **RuSPy** along with a histogram of the calculated electronic transitions (green sticks). Bottom: experimental (DCM, black trace) and TD DFT-calculated (purple trace) CD spectra of *S*-**RuSPy** along with a histogram of the calculated electronic transitions (green sticks).

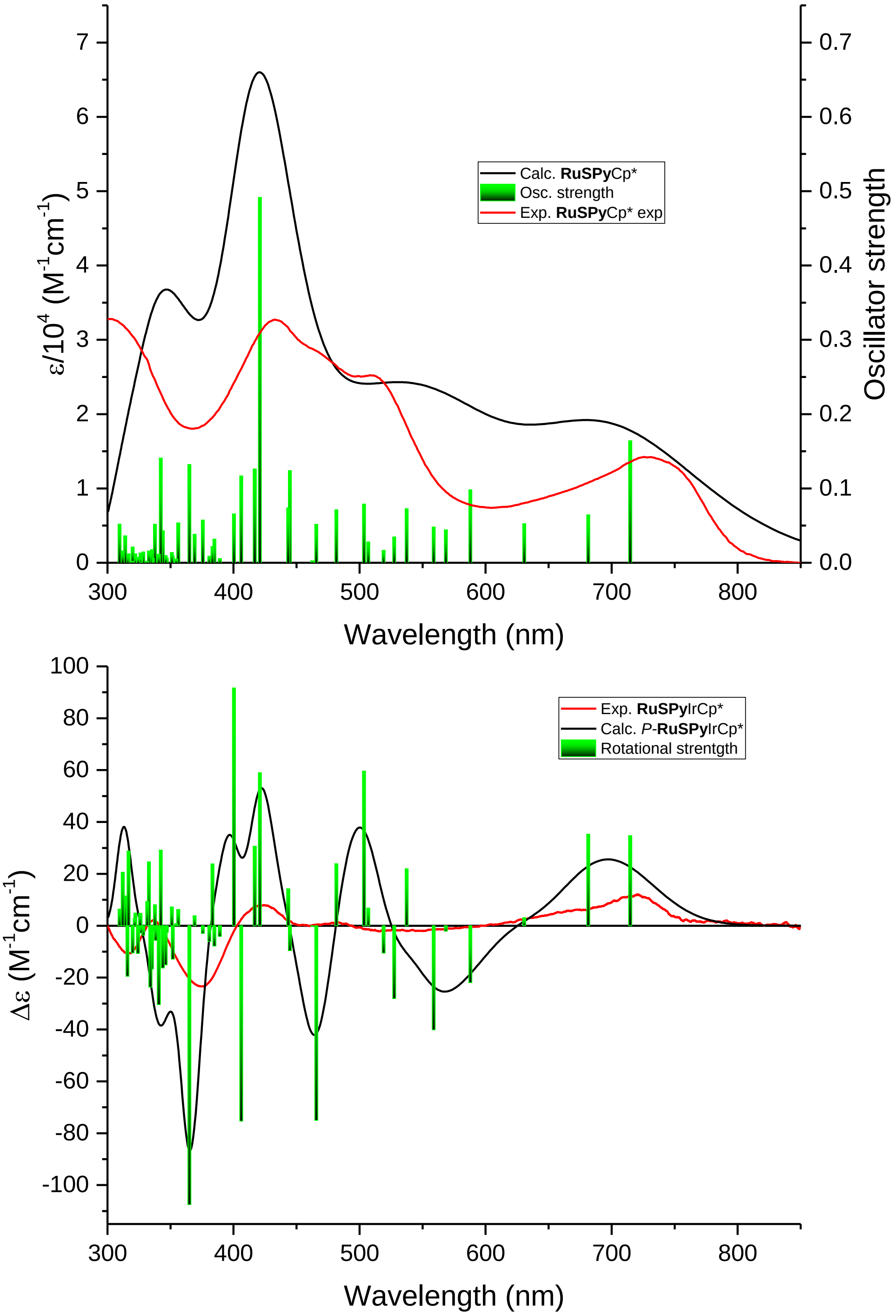

Figure S24. Top: experimental (DCM, red trace) and TD DFT-calculated (black trace) absorbance spectra of **RuSPy**IrCp* along with a histogram of the calculated electronic transitions (green sticks). Bottom: experimental (DCM, red trace) and TD DFT-calculated (black trace) CD spectra of *P*-**RuSPy**IrCp* along with a histogram of the calculated electronic transitions (green sticks).

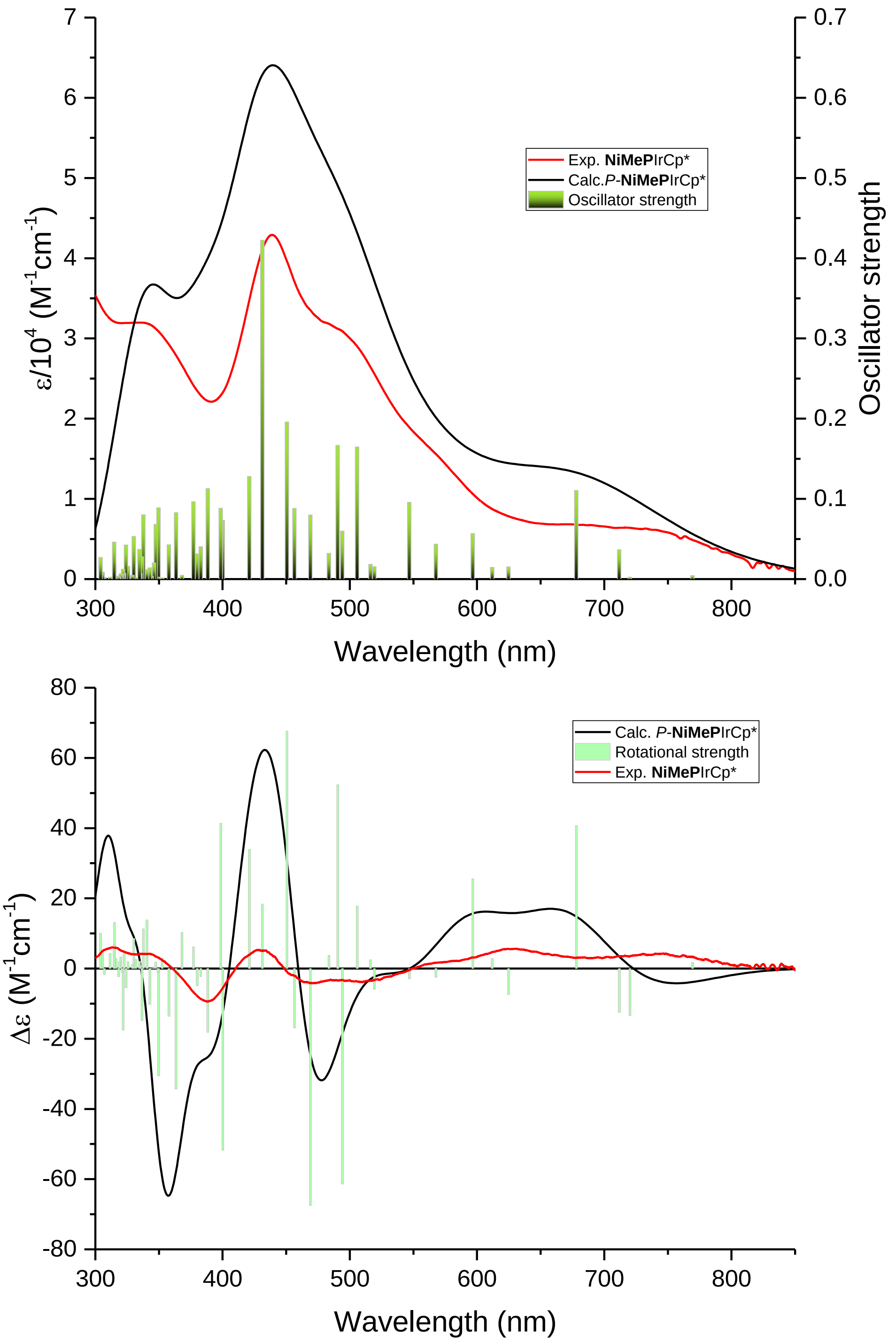

Figure S25. Top: experimental (DCM, red trace) and TD DFT-calculated (black trace) absorbance spectra of **NiMeP**IrCp* along with a histogram of the calculated electronic transitions (green sticks). Bottom: experimental (DCM, red trace) and TD DFT-calculated (black trace) CD spectra of *P*-**NiMeP**IrCp* along with a histogram of the calculated electronic transitions (green sticks).

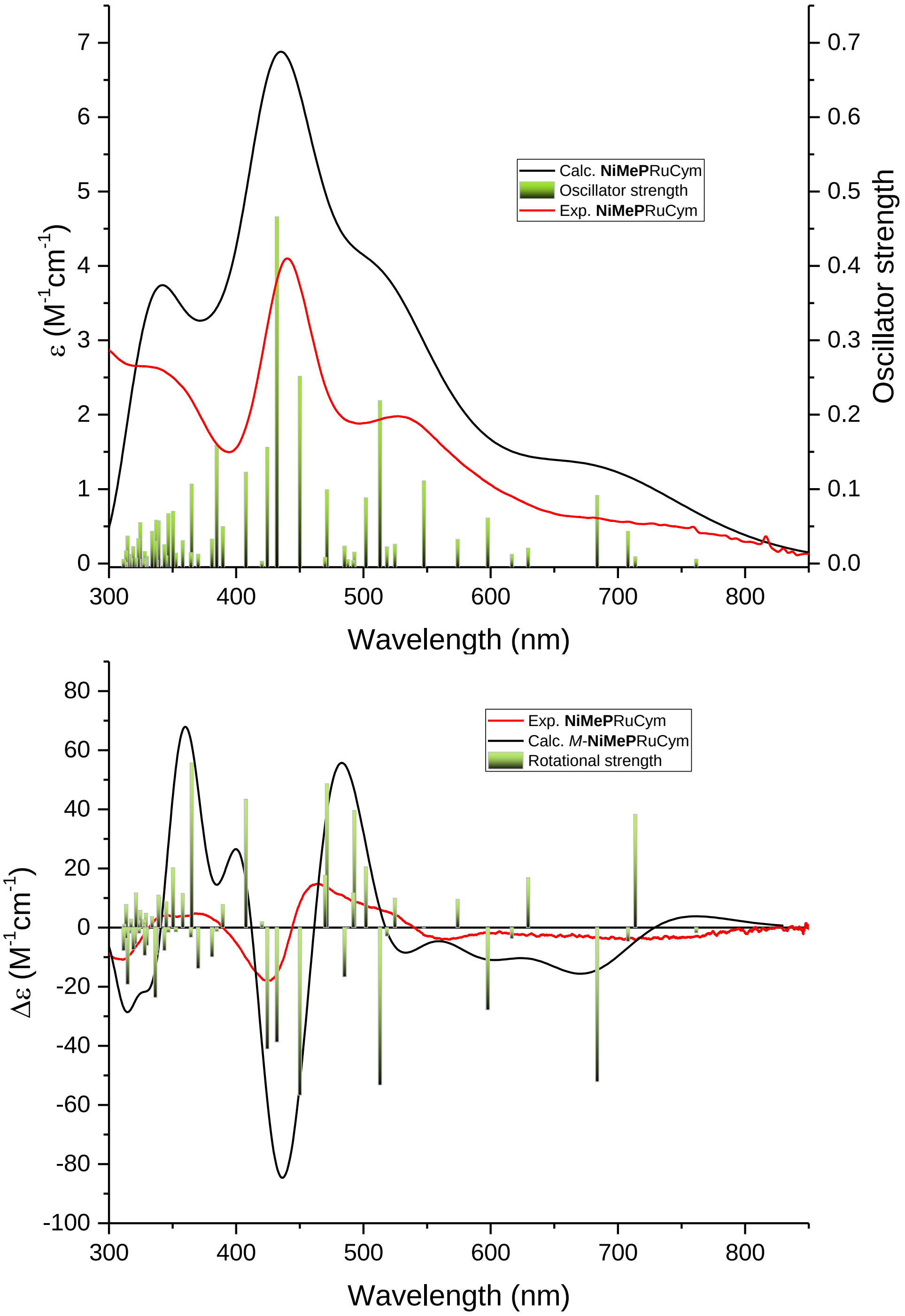

Figure S26. Top: experimental (DCM, red trace) and TD DFT-calculated (black trace) absorbance spectra of **NiMeP**RuCym along with a histogram of the calculated electronic transitions (green sticks). Bottom: experimental (DCM, red trace) and TD DFT-calculated (black trace) CD spectra of *M*- **NiMeP**RuCym along with a histogram of the calculated electronic transitions (green sticks).

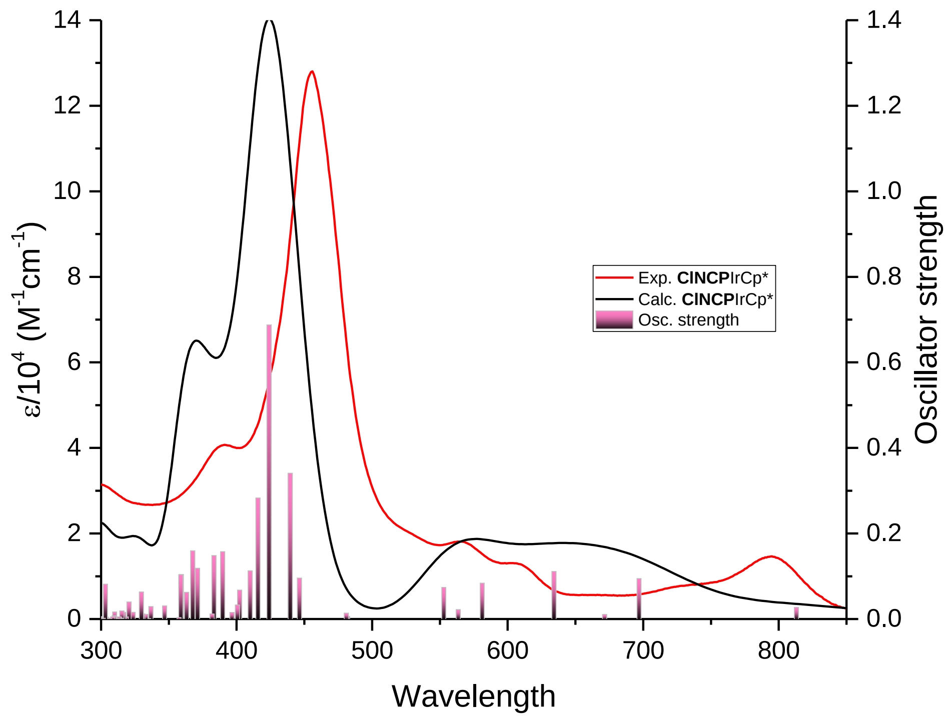

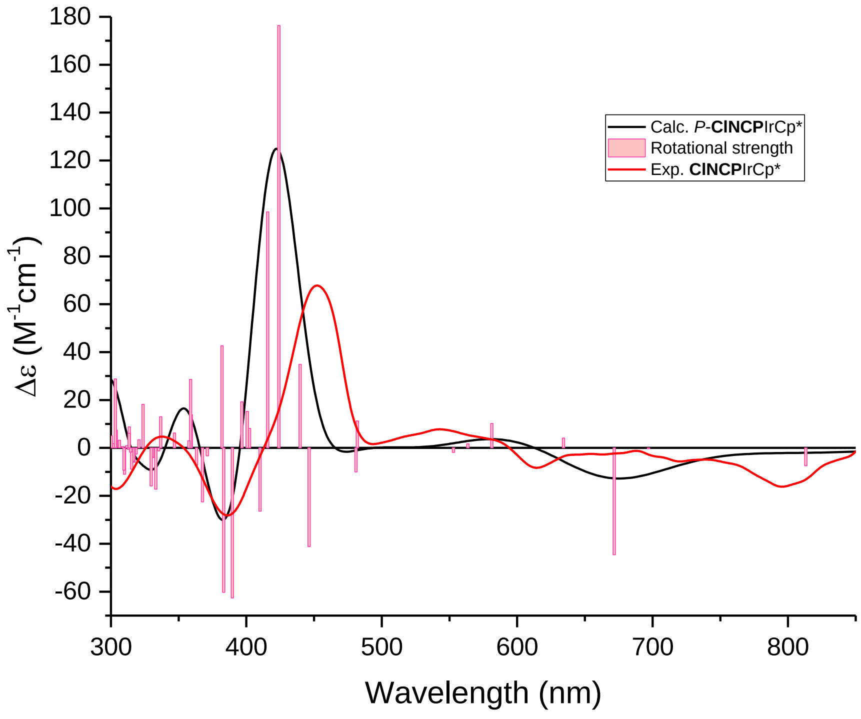

Figure S27. Top: experimental (DCM, red trace) and TD DFT-calculated (black trace) absorbance spectra of **ClNCP**IrCp* along with a histogram of the calculated electronic transitions (purple sticks). Bottom: experimental (DCM, red trace) and TD DFT-calculated (black trace) CD spectra of *P*-**ClNCP**IrCp* along with a histogram of the calculated electronic transitions (rose sticks).

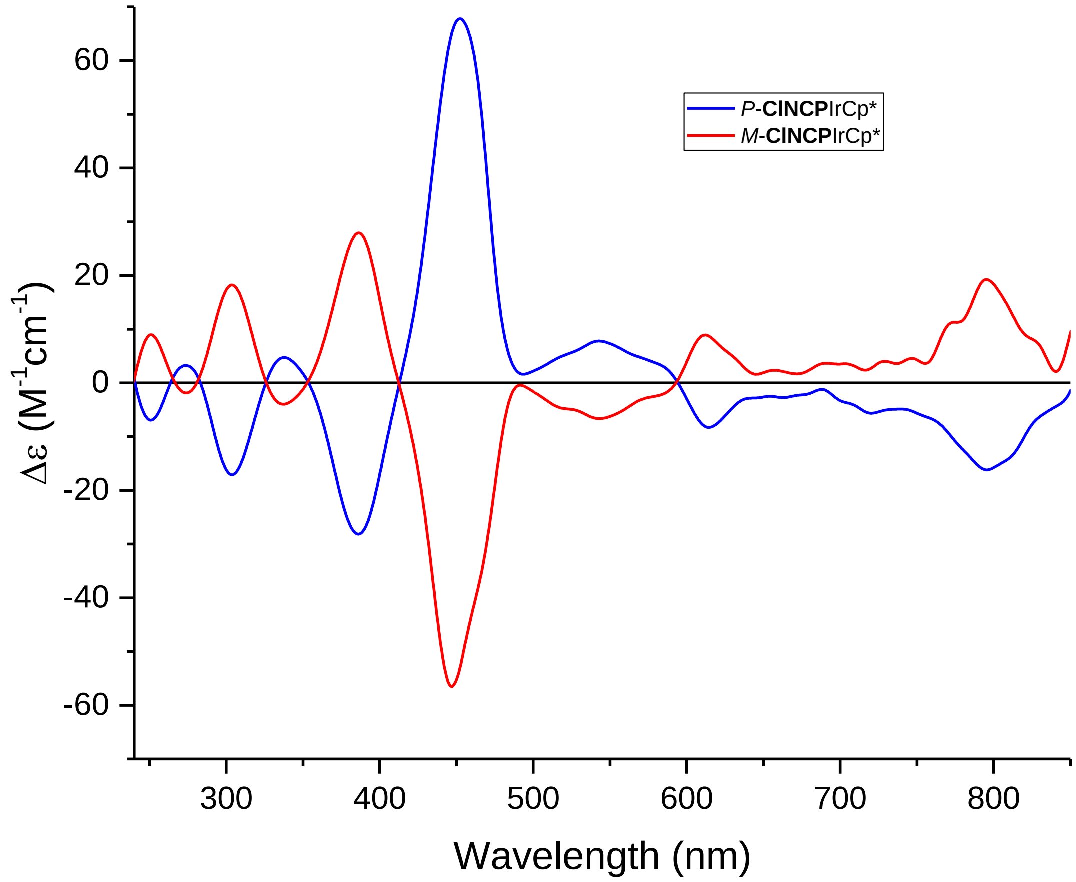

Figure S28. CD spectr[illegible] enantiomers of **ClNCP**IrCp* in DCM.

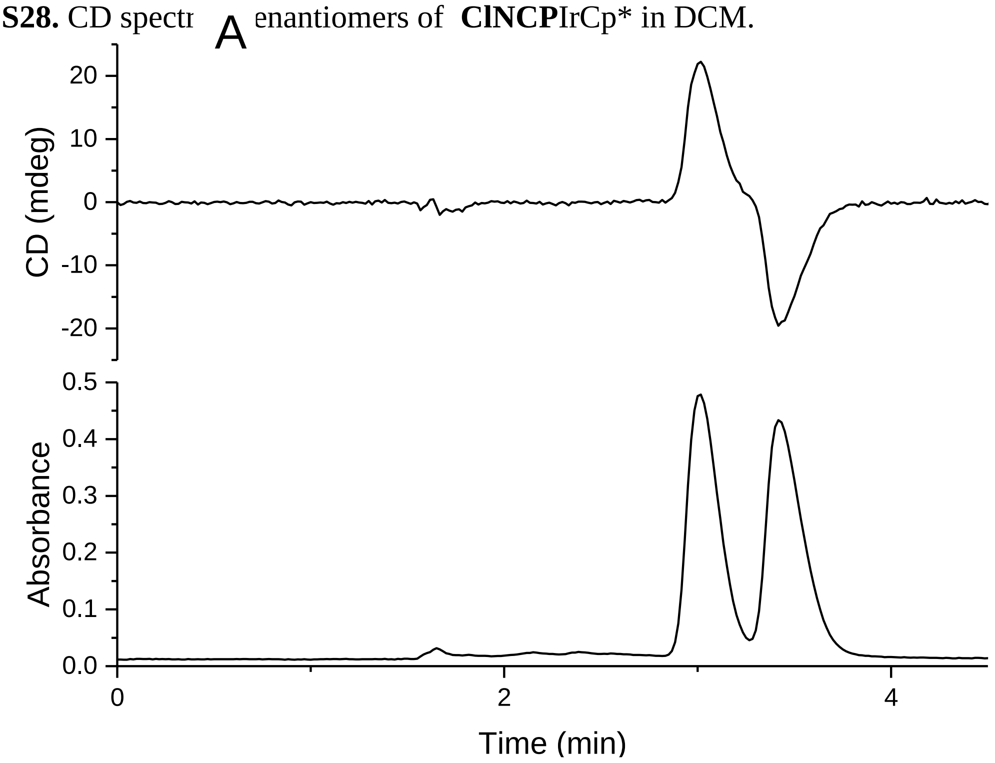

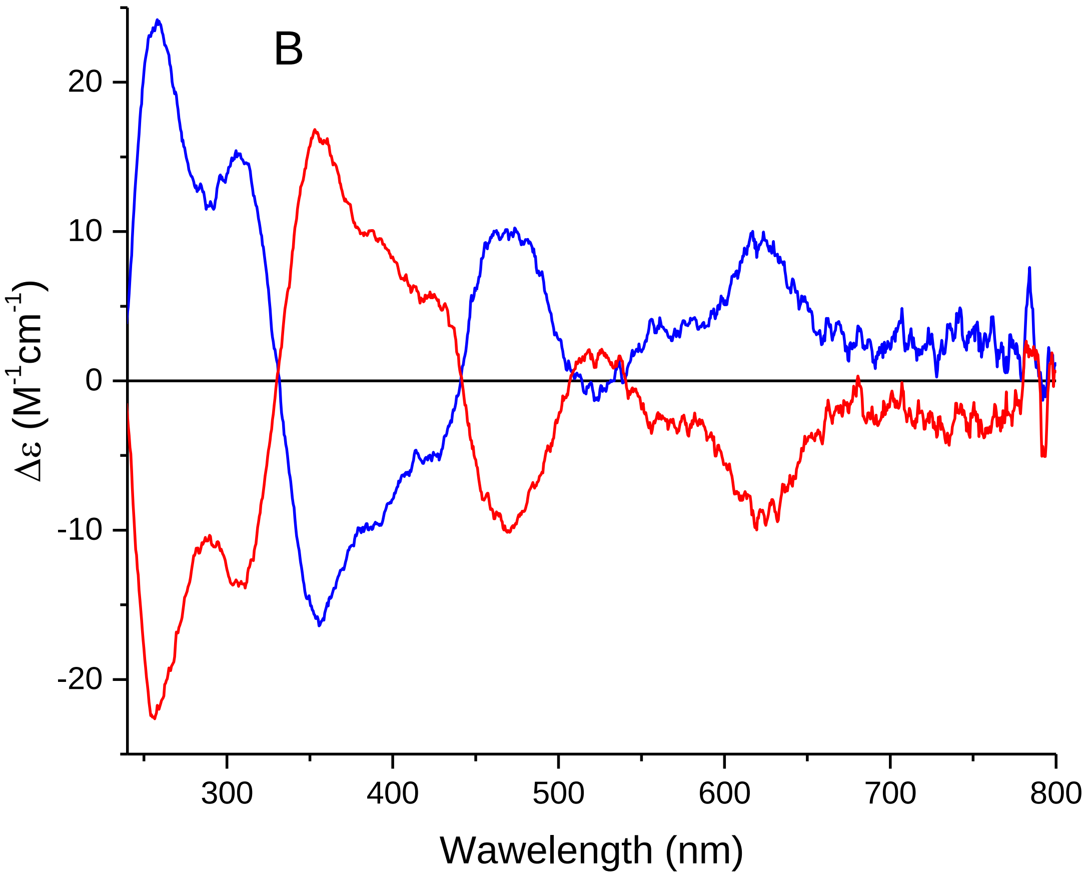

Figure S29. (A) HPLC profiles for **NiMeP**RhCp* on chiral stationary phase column (Chirex 3014, 40% hexane in DCM, 2 mL/min); detection at 635 nm; bottom, absorbance detection; top, CD detection. (B) CD spectra of enantiomers of **NiMeP**RhCp* in DCM.

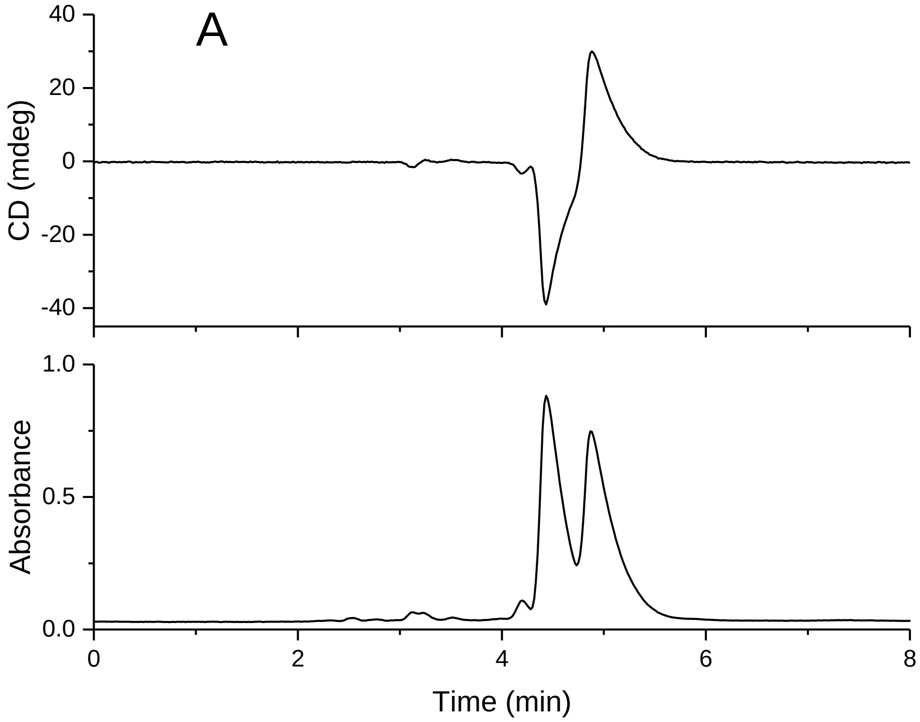

B

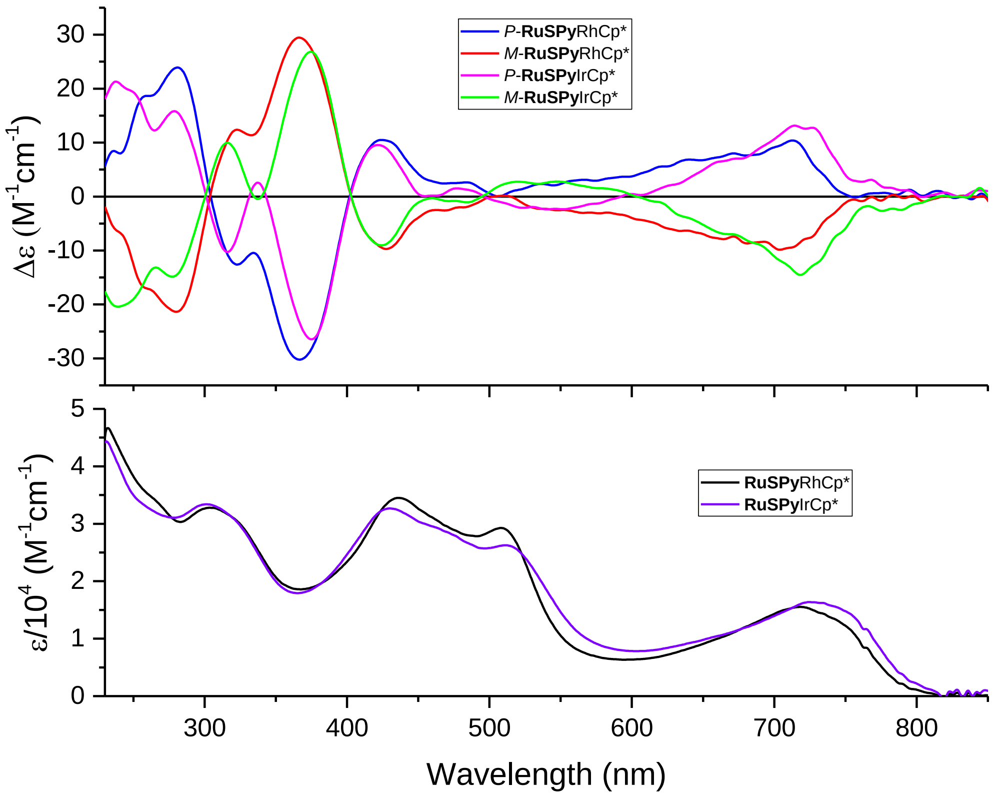

Figure S30. (A) HPLC profiles for **RuSPy**IrCp* on chiral stationary phase column (Chirex 3014, 50% hexane in DCM, 2 mL/min); detection at 370 nm; bottom, absorbance detection; top, CD detection. (B) CD spectra (top) and absorbance UV-vis spectrum (bottom) of enantiomers of **RuSPy**RhCp* and **RuSPy**IrCp* in DCM.

Redox properties

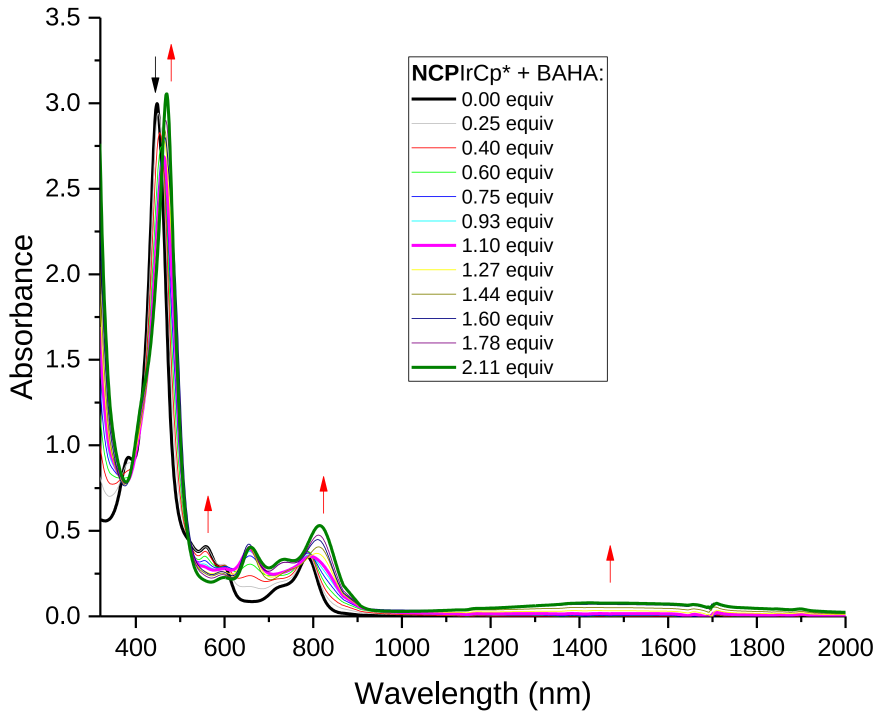

Figure 31. Spectrophotometric titration of dichloromethane solutions of **ClNCP**IrCp* with tris(4-bromophenyl)ammoniumyl hexachloroantimonate (BAHA). The black arrow indicates a direction of the absorbance changes before, while the red arrows – after addition of 1 equiv of BAHA.

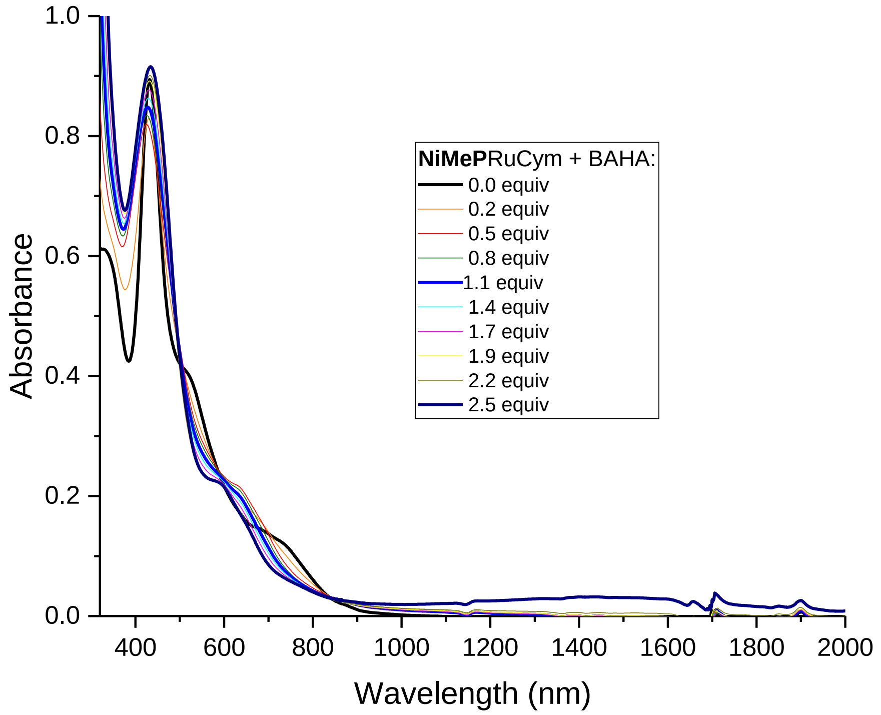

Figure S32. Spectrophotometric titration of dichloromethane solutions of **NiMeP**RuCym with tris(4-bromophenyl)ammoniumyl hexachloroantimonate (BAHA).

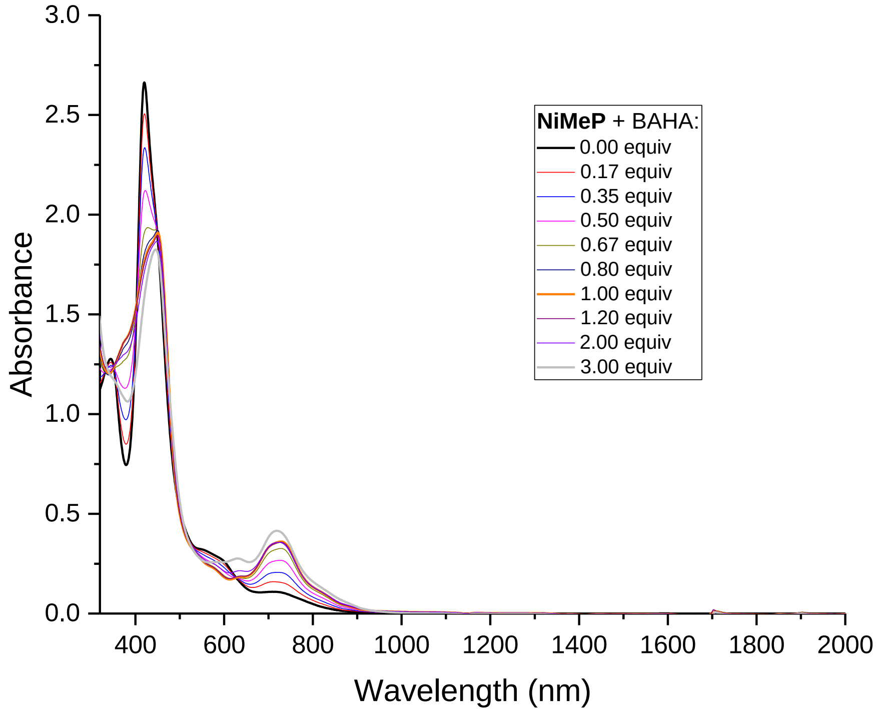

Figure S33. Spectrophotometric titration of dichloromethane solutions of **NiMeP** with tris(4-bromophenyl)ammoniumyl hexachloroantimonate (BAHA).

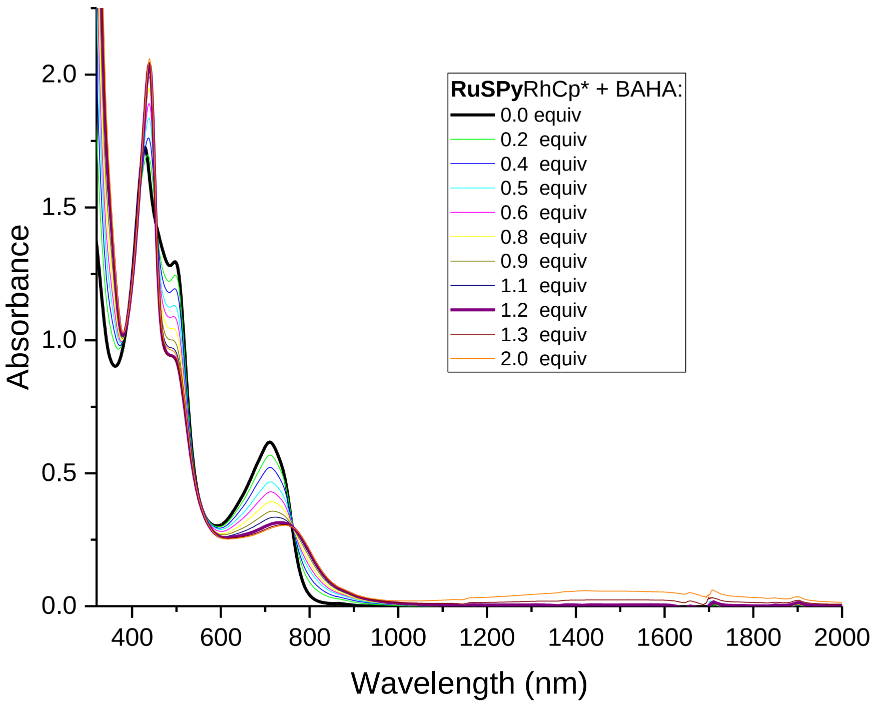

Figure S34. Spectrophotometric titration of dichloromethane solutions of **RuSPy**RhCp* with tris(4-bromophenyl)ammoniumyl hexachloroantimonate (BAHA).

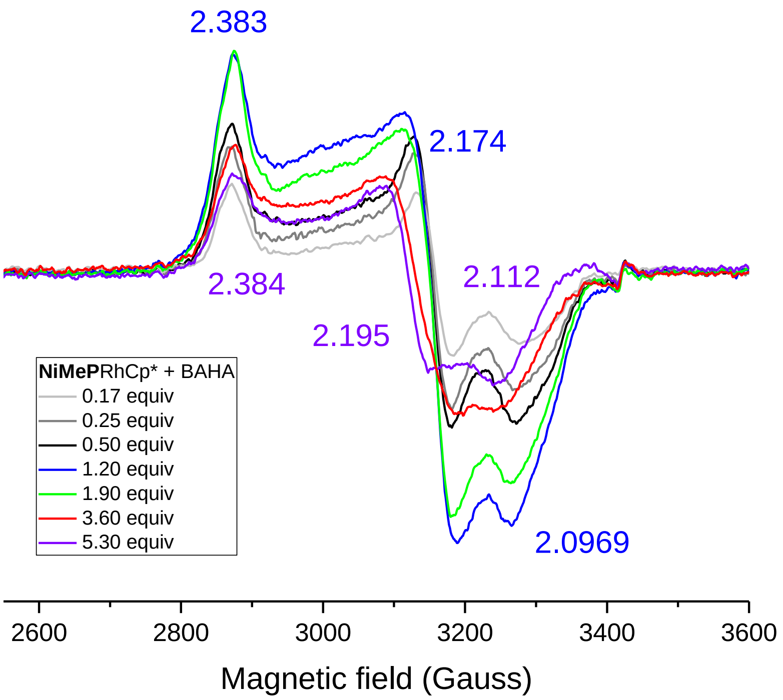

Figure S35. EPR-monitored titration of dichloromethane solutions of **NiMeP**RhCp* with tris(4-bromophenyl)ammoniumyl hexachloroantimonate (BAHA). The blue and purple numbers are Zeeman tensor components for the first and the second form observed upon oxidation. The spectra were recorded at 143 K.

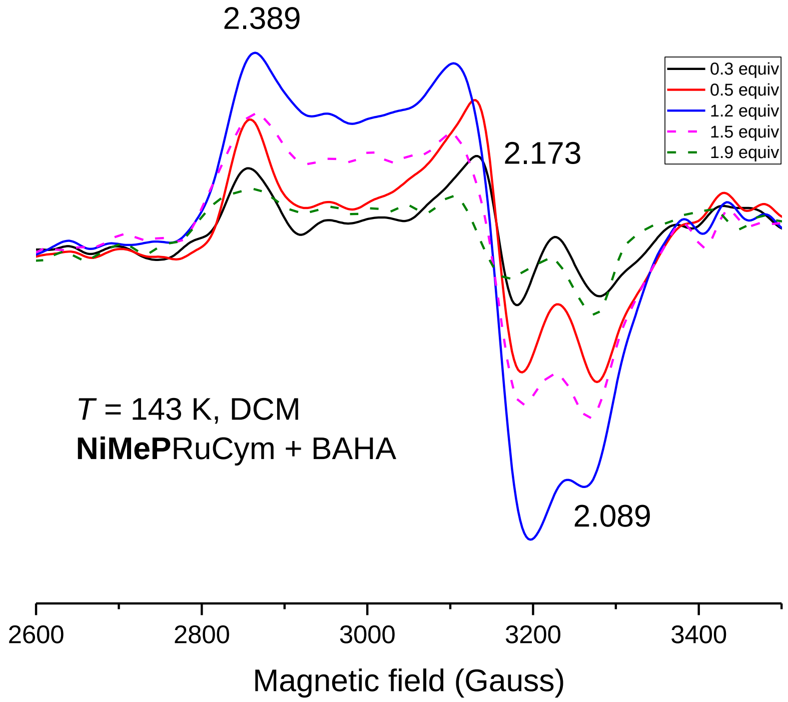

Figure S36. EPR-monitored titration of dichloromethane solutions of **NiMeP**RuCym with tris(4-bromophenyl)ammoniumyl hexachloroantimonate (BAHA). The Zeeman tensor component values are associated with each of the spectral features. The spectra were recorded at 143 K.

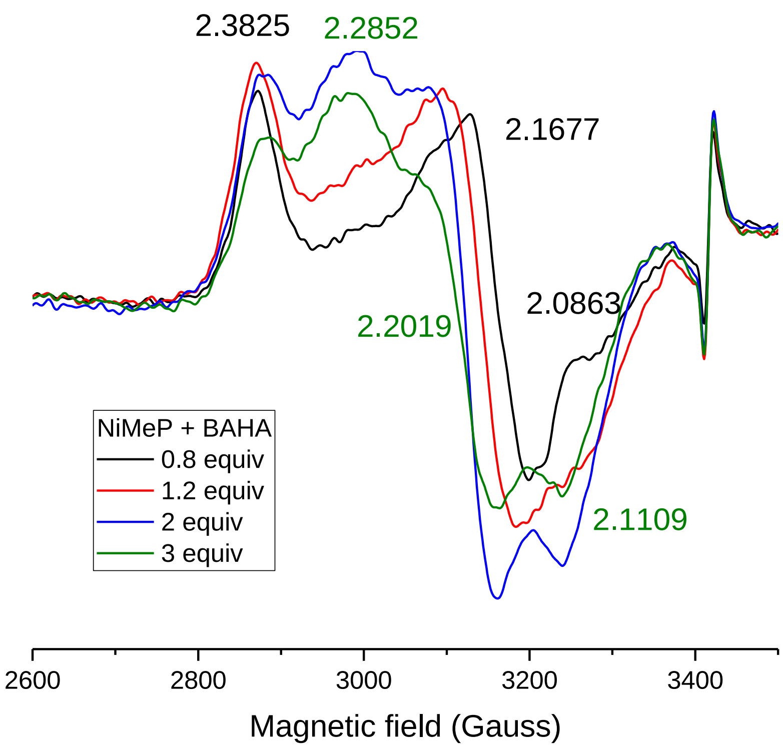

Figure S37. EPR-monitored titration of dichloromethane solutions of **NiMeP** with tris(4-bromophenyl)ammoniumyl hexachloroantimonate (BAHA). The black and green numbers are Zeeman tensor component values for the first and the second form observed upon oxidation. The spectra were recorded at 143 K.

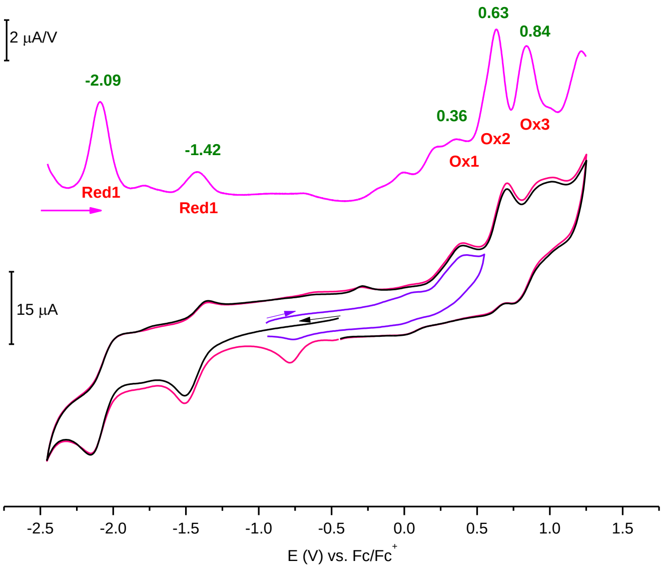

Figure S38. Cyclic (lower traces) and differential pulse (upper trace) voltammograms of **ClNCP**. The experiments were carried out in a dichloromethane solution of $[Bu_4N]PF_6$ (0.1 M) using a glassy carbon working electrode, a platinum wire as an auxiliary electrode, and Ag/AgCl as a pseudoreference electrode. The green numbers associated with DP peaks are electrode potentials in volts.

Computational methods

Density functional theory (DFT) calculations were performed by using the Gaussian 16 program[S1]. DFT calculations were carried out using B3LYP functional[S2,S3] and 6-31G(d,p) basis set with pseudopotential LANL2DZ for Ru, Rh, Ir. Optimizations were performed in vacuo. Energy minima were obtained as no imaginary frequencies were found. TD-DFT calculations were done at the same level of theory except for **RuSPy,** which performed best with a B3LYP/def2-SVP[S4] setup. All were solved for 60 states and including implicit solvent (PCM; dichloromethane). The electronic transitions and UV-vis spectra were analyzed by means of the GaussSum program[S5]. The transitions were convoluted by Gaussian curves with 2000 cm^{-1} half line width for absorption spectra and 0.15 eV for CD spectra. The computational details are collected in Table S1.

Table S1. Computational details for the optimized structures of compounds

Structure / Name[a)]	SCF E<br>a.u.	ZPV[b)]<br>a.u.	lowest freq.<br>cm^{-1}	E<br>a.u.	ΔH<br>a.u.	ΔG[c)]<br>a.u.	HOMO<br>eV	LUMO<br>eV	HLG<br>eV
**NiMeP**IrCp*/KS6-OF_a	-4414.585796	-4413.745253	9.16	-4413.691011	-4413.690067	-4413.834618	5.08	2.79	2.29
**RuSPy**RhCp*/KS5-OF_a	-3724.443542	-3723.553270	14.02	-3723.492404	-3723.491460	-3723.649352	5.05	2.86	2.19
**NiMeP**RuCym/KS4-OF_a	-4403.146337	-4402.314284	9.96	-4402.261927	-4402.260983	-4402.401432	5.03	2.76	2.29
**RuSPy**IrCp*/KS3-OF_a	-3719.642448	-3718.751846	13.99	-3718.691107	-3718.690163	-3718.847824	5.00	2.85	2.15
**RuSPy**/KS1-OF_a	-2765.144310	-2764.468467	13.45	-2764.423777	-2764.422833	-2764.547841	5.31	2.95	2.36
**ClNCP**IrCp*/ KS7-OF_a	-4706.605393	-4705.810567	8.36	-4705.752947	-4705.752003	-4705.907426	5.08	3.06	2.02

a) Data set name (Cartesian coordinated available as Name.pdb files). b) Zero-point vibrational energy. [c] Gibbs free energy.

Crystallographic data

Table S2. Crystal data for **NiMeP**IrCp*

Crystal data	
**Chemical formula**	$C_{55}H_{44}N4ClIrNi$
***M*r**	1047.30
**Crystal system, space group**	$P2_1/c$
**Temperature (K)**	100
**a, b, c (Å)**	14.575 (1), 12.776 (1), 26.154 (2)
**α, β, γ (°)**	β = 98.12 (1)
**V (Å³)**	4821.52 (6)
Z	4
**Radiation type**	Cu Kα
**μ (mm⁻¹)**	6.56
**Crystal size (mm)**	0.17 × 0.12 × 0.05
**Data collection**	
**Diffractometer**	XtaLAB Synergy R, DW system, HyPix-Arc 150
**No. of measured, independent and observed [I > 2σ(I)] reflections**	46661, 8181, 7594
**R_{int}**	0.031
**$(\sin θ/λ)_{max}$ (Å⁻¹)**	0.588
**Refinement**	
$R[F^2 > 2σ(F^2)], wR(F^2), S$	0.021, 0.055, 1.07
**No. of reflections**	8181
**No. of parameters**	565
**H-atom treatment**	H-atom parameters constrained

$\Delta\rho_{max}$, $\Delta\rho_{min}$ (e Å^{-3})	0.90, −0.70

Table S3. Crystal data for **NiMeP**RuCym

Crystal data	
Chemical formula	$C_{55}H_{43}N_4ClNiRu·CH_3OH$
M_r	987.20
Crystal system, space group	Triclinic, *P*-1
Temperature (K)	120
a, b, c (Å)	10.162 (3), 13.803 (5), 16.258 (5)
α, β, γ (°)	103.06 (3), 99.94 (2), 102.59 (3)
V (Å^3)	2108.23 (12)
Z	2
Radiation type	Cu Kα
μ (mm^{-1})	4.42
Crystal size (mm)	0.30 × 0.16 × 0.09
**Data collection**	
Diffractometer	Xcalibur, Onyx
No. of measured, independent and observed [I > 2σ(I)] reflections	15709, 6705, 5429
R_{int}	0.092
$(\sin \theta/\lambda)_{max}$ (Å^{-1})	0.575
**Refinement**	
$R[F^2 > 2\sigma(F^2)]$, $wR(F^2)$, S	0.062, 0.164, 1.01
No. of reflections	6705
No. of parameters	582
H-atom treatment	H-atom parameters constrained
$\Delta\rho_{max}$, $\Delta\rho_{min}$ (e Å^{-3})	0.98, −0.96

Table S4. Crystal Data for **RuSPy**IrCp*

Crystal data	
Chemical formula	$C_{60}H_{45}$ $N_5SClIrORu·3(C_6H_6)$
M_r	1447.11
Crystal system, space group	Monoclinic, *P2₁/c*
Temperature (K)	100
a, b, c (Å)	16.195 (1), 18.341 (1), 21.005 (1)
α, β, γ (°)	β = 98.12 (1)
V (Å^3)	6177.28 (7)
Z	4
Radiation type	Cu Kα
μ (mm^{-1})	7.21
Crystal size (mm)	0.10 × 0.10 × 0.05
**Data collection**	
Diffractometer	XtaLAB Synergy R, HyPix-Arc 150
No. of measured, independent and observed [I > 2σ(I)] reflections	63950, 10871, 9938
R_{int}	0.035
$(\sin \theta/\lambda)_{max}$ (Å^{-1})	0.595
**Refinement**	
$R[F^2 > 2\sigma(F^2)]$, $wR(F^2)$, S	0.030, 0.071, 1.11
No. of reflections	10871
No. of parameters	798
H-atom treatment	H-atom parameters constrained
$\Delta\rho_{max}$, $\Delta\rho_{min}$ (e Å^{-3})	1.30, −0.76

Table S5. Crystal data for **RuSPy**RhCp*

Crystal data	
Chemical formula	$C_{60}H_{45}ClN_5OSRhRu·3(C6H6)$
M_r	1357.82
Crystal system, space group	Monoclinic, *P2₁/c*
Temperature (K)	100
a, b, c (Å)	16.195 (1), 18.341 (1), 21.005 (1)
α, β, γ (°)	β = 97.72 (1)
V (Å³)	6158.37 (14)
Z	4
Radiation type	Cu Kα
μ (mm⁻¹)	5.28
Crystal size (mm)	0.11 × 0.06 × 0.04
**Data collection**	
Diffractometer	XtaLAB Synergy R, HyPix-Arc 150
No. of measured, independent and observed [I > 2σ(I)] reflections	56514, 9813, 8408
R_{int}	0.051
$(\sin θ/λ)_{max}$ (Å⁻¹)	0.575
**Refinement**	
$R[F^2 > 2σ(F^2)], wR(F^2), S$	0.045, 0.112, 1.10
No. of reflections	9813
No. of parameters	798
H-atom treatment	H-atom parameters constrained
$Δ_{ρmax}, Δ_{ρmin}$ (e Å⁻³)	1.59, −0.72

Table S6. Crystal data for **ClNCP**IrCp*

Crystal data	
Chemical formula	$C_{54}H_{40}N_4Cl_5Ir·3(CH_2Cl_2)$
M_r	1369.13
Crystal system, space group	Monoclinic, *P2₁/c*
Temperature (K)	100
a, b, c (Å)	14.880 (1), 21.737 (2), 16.922 (2)
α, β, γ (°)	β = 95.28 (1)
V (Å³)	5450.23 (9)
Z	4
Radiation type	Cu Kα
μ (mm⁻¹)	10.05
Crystal size (mm)	0.18 × 0.07 × 0.05
**Data collection**	
Diffractometer	XtaLAB Synergy R, HyPix-Arc 150
No. of measured, independent and observed [I > 2σ(I)] reflections	51237, 9259, 8378
R_{int}	0.033
$(\sin θ/λ)_{max}$ (Å⁻¹)	0.588
**Refinement**	
$R[F^2 > 2σ(F^2)], wR(F^2), S$	0.046, 0.126, 1.05
No. of reflections	9259
No. of parameters	711
H-atom treatment	H-atom parameters constrained
$Δ_{ρmax}, Δ_{ρmin}$ (e Å⁻³)	2.52, −0.85

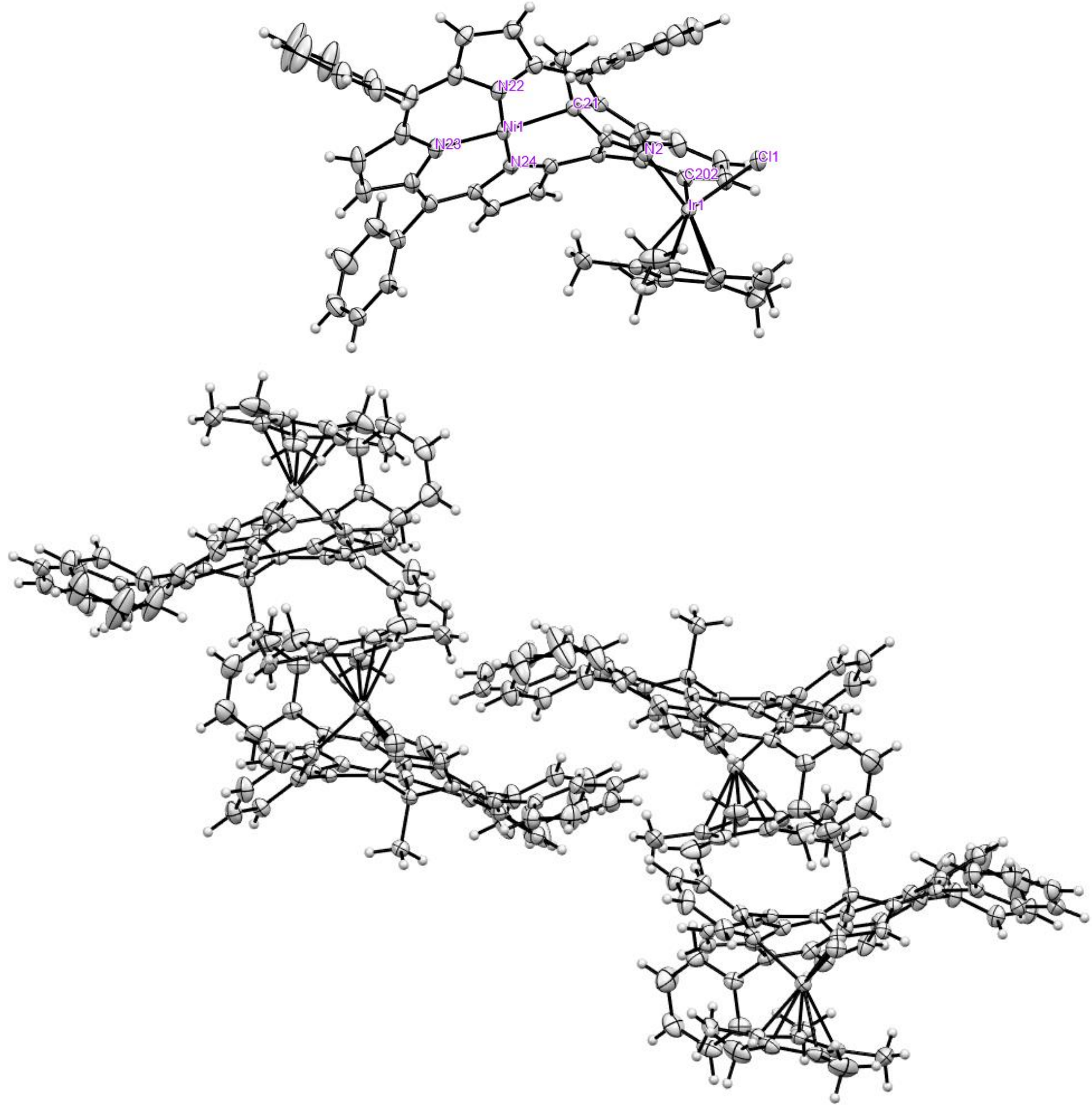

Figure S39. ORTEP drawing (with 50% thermal ellipsoids) of the asymmetric part (top) and the unit cell packing diagram (bottom, view along axis *a*) of the crystal structure of **NiMeP**IrCp*.

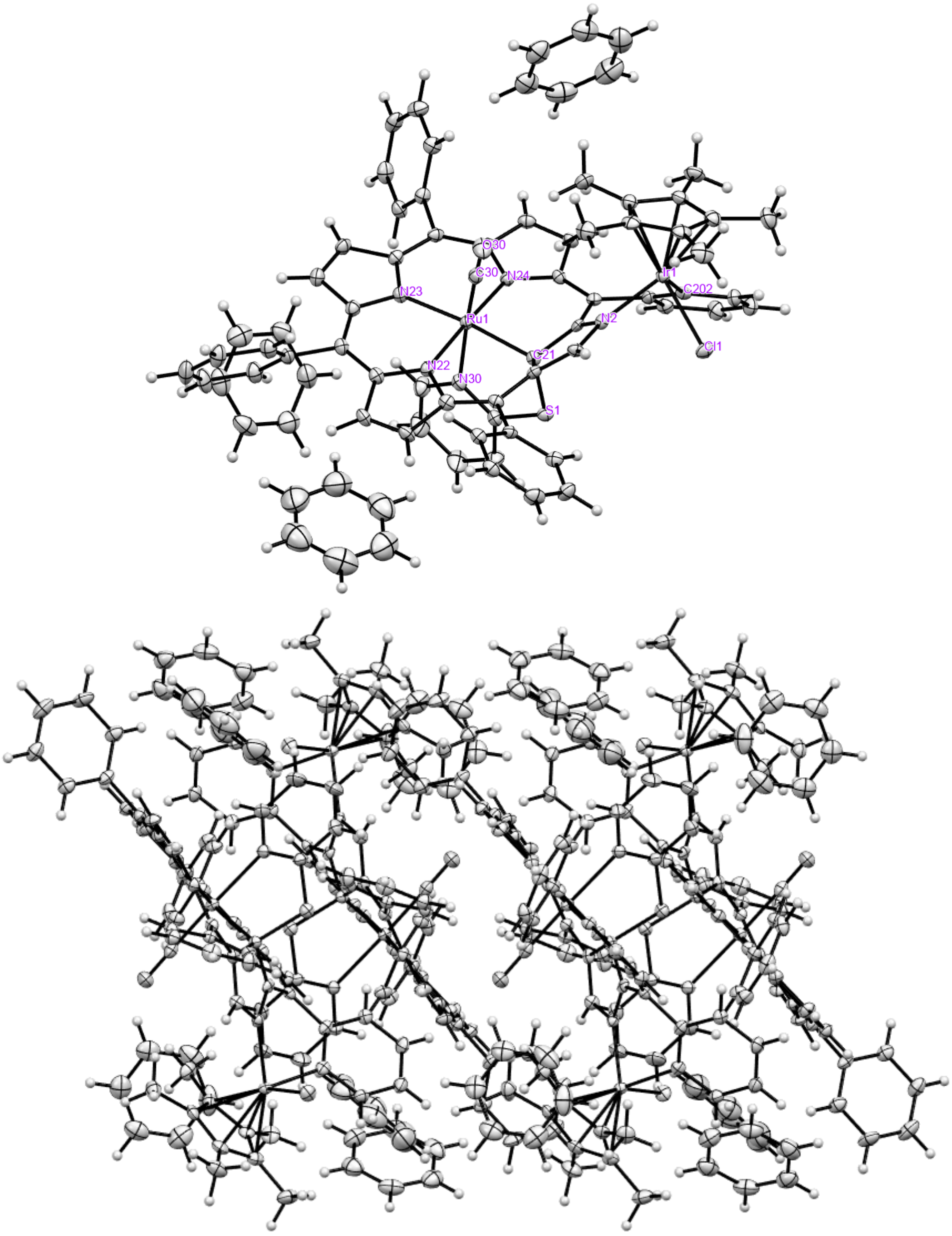

Figure S40. ORTEP drawing (with 50% thermal ellipsoids) of the asymmetric part (top) and the unit cell packing diagram (bottom, view along axis *b*) of the crystal structure of **RuSPy**IrCp*.

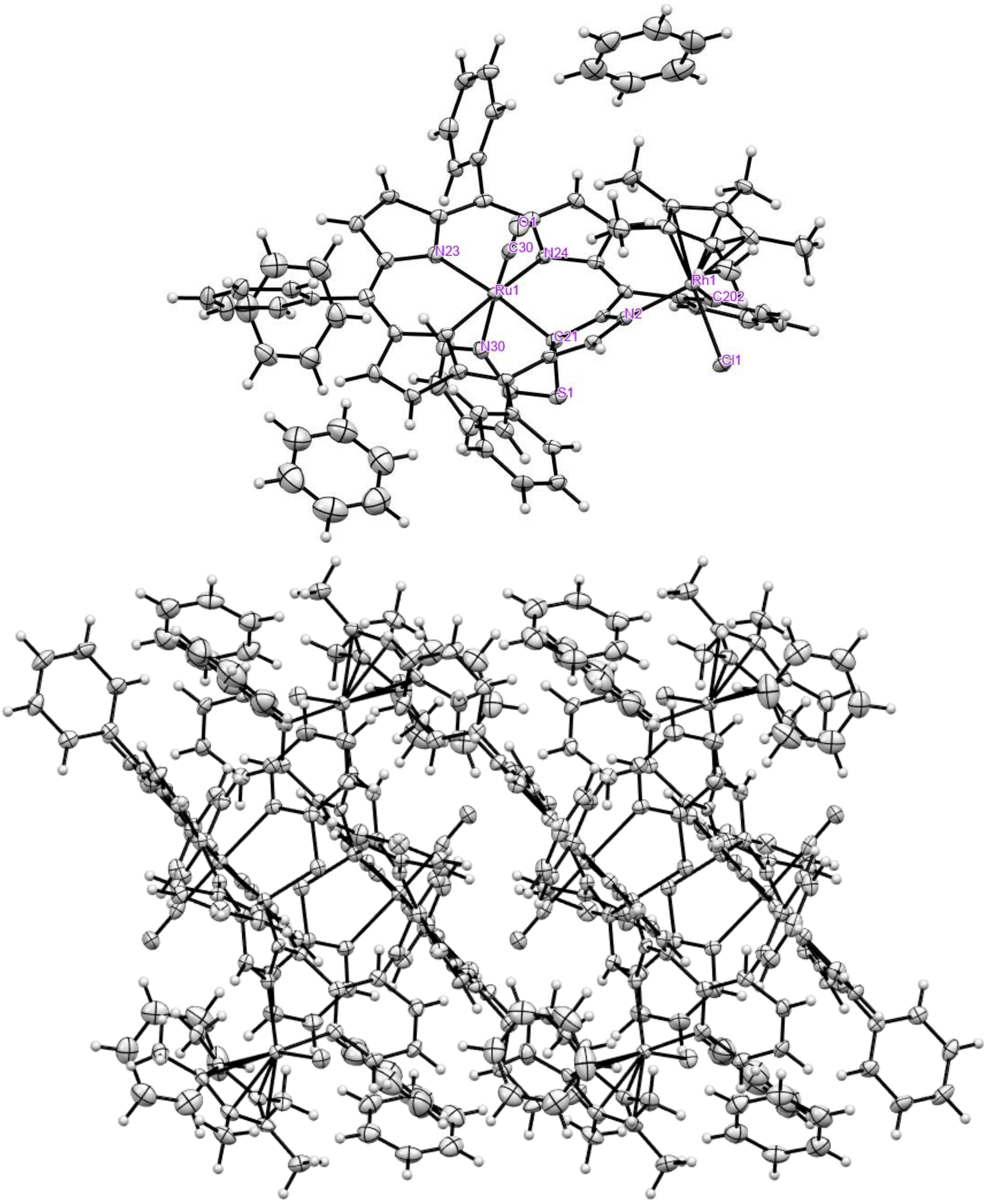

Figure S41. ORTEP drawings (with 50% thermal ellipsoids) of the asymmetric part and the unit cell packing diagram (bottom, view along axis *b*) of the crystal structure of **RuSPy**RhCp*.

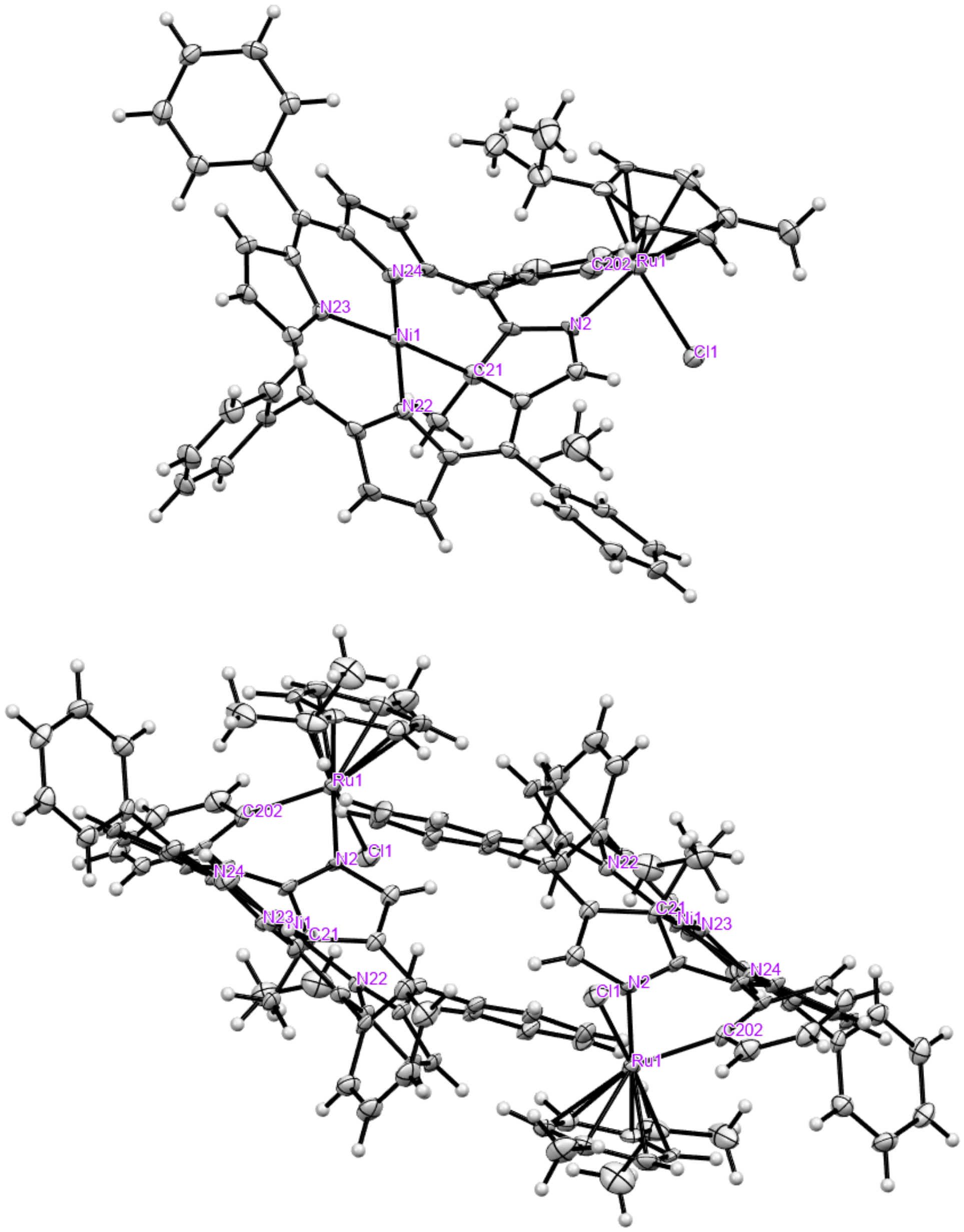

Figure S42. ORTEP drawings (with 50% thermal ellipsoids) of the asymmetric part (top) and the unit cell packing diagram (bottom, view along axis *b*) of the crystal structure of **NiMeP**RuCym.

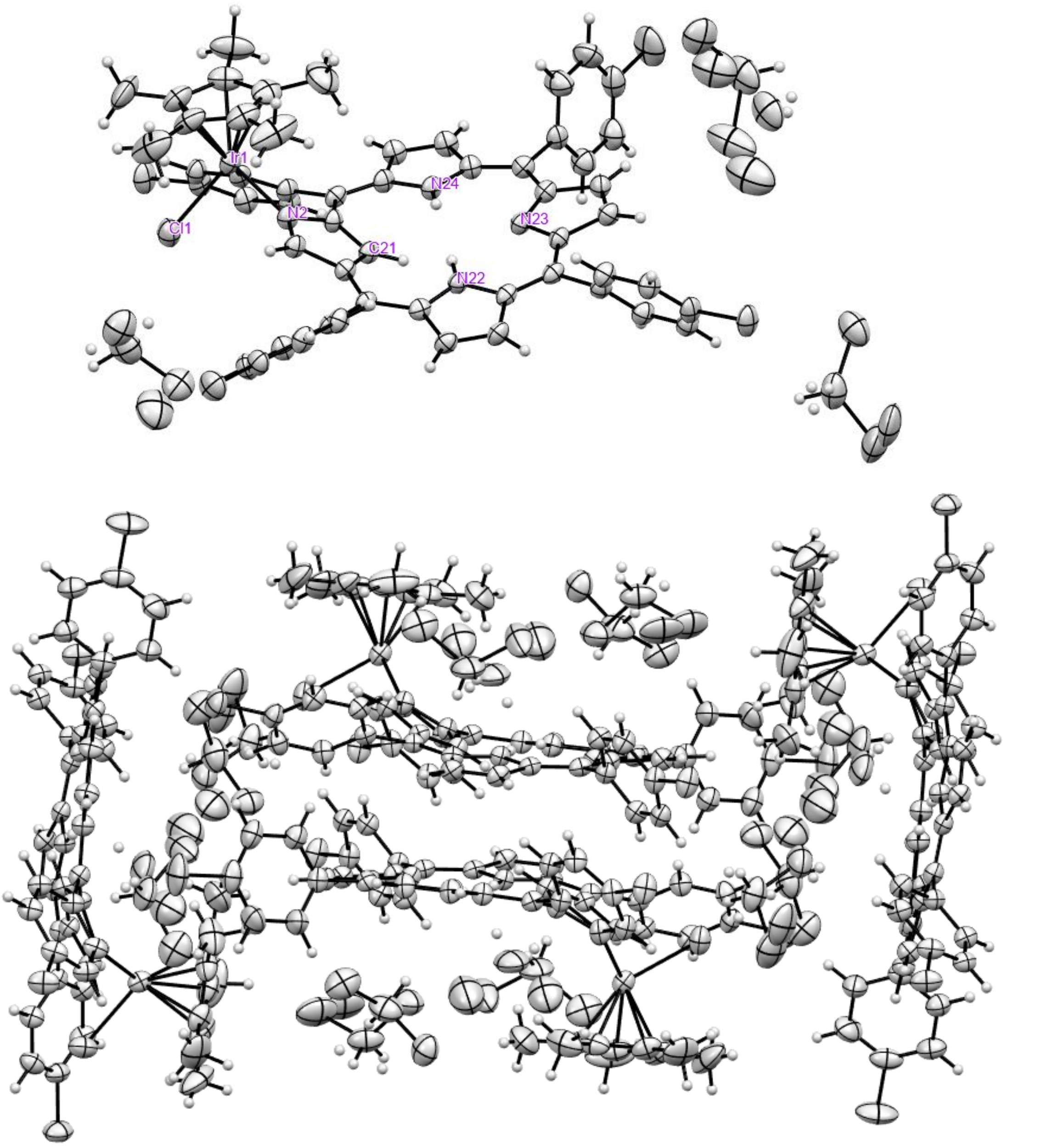

Figure S43. ORTEP drawing (with 50% thermal ellipsoids) of the asymmetric part (top) and the unit cell packing diagram (bottom, view along axis *a*) of the crystal structure of **ClNCP**IrCp*.

Table S7. Electronic transitions calculated for **RuSPy** by means of TD DFT

No.	Energy (cm^{-1})	Wavelength (nm)	Osc. Strength	Major contribs
1	14973	668	0.0836	H-1->L+1 (12%), HOMO->LUMO (83%)
2	16104	621	0.015	H-1->LUMO (51%), HOMO->L+1 (40%)
3	16859	593	0.0268	H-2->LUMO (74%), HOMO->L+1 (14%)
4	17880	559	0.0805	H-3->LUMO (36%), H-2->LUMO (18%), HOMO->L+1 (20%)
5	18311	546	0.0509	H-3->L+1 (23%), H-2->L+1 (20%), H-1->L+1 (38%)

6	19903	502	0.0525	H-4->LUMO (30%), H-2->L+1 (39%), H-1->L+1 (10%)
7	20552	487	0.1577	H-5->LUMO (14%), H-4->L+1 (10%), H-3->LUMO (32%), H-2->L+1 (15%), H-1->LUMO (14%)
8	20979	477	0.0026	H-4->LUMO (22%), H-3->L+1 (46%), H-2->L+1 (16%)
9	22118	452	0.0072	H-5->LUMO (49%), H-4->L+1 (38%)
10	22681	441	0.2598	H-5->L+1 (22%), H-4->LUMO (24%), H-3->L+1 (22%), H-1->L+1 (15%)
11	23889	419	0.8599	H-5->LUMO (19%), H-4->L+1 (33%), H-1->LUMO (12%), HOMO->L+1 (13%)
12	24259	412	0.4572	H-5->L+1 (62%), H-1->L+1 (11%)
13	27300	366	0.0052	HOMO->L+3 (91%)
14	27750	360	0.0703	H-7->LUMO (41%), H-6->LUMO (29%), HOMO->L+2 (14%)
15	27841	359	0.0099	HOMO->L+2 (68%)
16	27943	358	0.1345	H-7->LUMO (30%), H-6->LUMO (56%)
17	28613	349	0.0587	H-9->LUMO (44%), H-6->L+1 (27%)
18	28773	348	0.055	H-8->LUMO (72%)
19	28865	346	0.0283	H-9->LUMO (43%), H-8->LUMO (10%), H-6->L+1 (16%)
20	29034	344	0.0028	H-14->LUMO (13%), H-1->L+2 (12%), HOMO->L+4 (27%)
21	29223	342	0.0046	H-10->LUMO (76%)
22	29446	340	0.0236	H-14->LUMO (30%), H-11->LUMO (12%)
23	29637	337	0.0018	H-1->L+3 (74%)
24	29744	336	0.0191	H-7->L+1 (48%), H-6->L+1 (16%)
25	29946	334	0.0603	H-1->L+4 (10%), HOMO->L+4 (37%)
26	30130	332	0.0102	H-13->LUMO (41%), H-12->LUMO (26%), H-11->LUMO (12%)
27	30210	331	0.0132	H-12->LUMO (19%), H-9->L+1 (34%), H-8->L+1 (16%)
28	30306	330	0.0041	H-14->L+1 (13%), H-9->L+1 (22%), H-1->L+2 (13%)
29	30361	329	0.0287	H-12->LUMO (25%), H-11->LUMO (25%), H-8->L+1 (26%)
30	30508	328	0.015	H-14->L+1 (16%), H-9->L+1 (13%), H-8->L+1 (28%)
31	30619	327	0.0048	H-14->LUMO (16%), H-13->LUMO (26%), H-12->LUMO (18%), H-11->LUMO (15%)
32	30734	325	0.009	H-2->L+2 (19%), H-2->L+3 (18%)
33	30780	325	0.014	H-2->L+3 (60%)
34	30845	324	0.0189	H-10->L+1 (67%)
35	31152	321	0.0738	H-2->L+2 (45%), H-1->L+2 (12%)
36	31573	317	0.0061	H-14->L+1 (32%), H-11->L+1 (53%)
37	31660	316	0.0035	H-16->LUMO (20%), H-15->LUMO (39%), H-13->L+1 (18%)
38	31825	314	0.0021	HOMO->L+5 (86%)
39	31884	314	0.0198	H-16->LUMO (10%), H-15->LUMO (11%), H-13->L+1 (45%), HOMO->L+5 (12%)
40	32098	312	0.0051	H-12->L+1 (82%)
41	32242	310	0.0126	H-16->LUMO (33%), H-15->LUMO (21%), H-2->L+4 (17%)
42	32392	309	0.0398	H-16->LUMO (15%), H-2->L+4 (28%)
43	32544	307	0.0419	H-3->L+3 (82%)
44	32675	306	0.0466	H-3->L+2 (57%), H-1->L+4 (13%)
45	32988	303	0.0056	H-16->L+1 (40%), H-15->L+1 (39%)
46	33114	302	0.002	H-17->LUMO (77%)

47	33366	300	0.0614	H-18->LUMO (10%), HOMO->L+11 (12%), HOMO->L+13 (23%)
48	33621	297	0.0317	H-18->LUMO (18%), H-16->L+1 (14%), H-3->L+4 (18%), H-1->L+4 (11%)
49	33685	297	0.0137	H-16->L+1 (22%), H-15->L+1 (38%)
50	33886	295	0.0801	H-18->LUMO (42%), H-3->L+4 (12%), H-1->L+4 (12%)
51	34012	294	0.0005	H-4->L+3 (86%)
52	34235	292	0.0131	H-4->L+2 (20%), H-1->L+13 (11%)
53	34374	291	0.0122	H-1->L+5 (88%)
54	34546	289	0.0004	H-4->L+2 (41%)
55	34656	289	0.0561	H-5->L+3 (69%), H-2->L+5 (16%)
56	34726	288	0.0413	H-18->L+1 (45%)
57	35042	285	0.0758	H-17->L+1 (11%), H-2->L+13 (20%)
58	35062	285	0.0249	H-17->L+1 (43%), HOMO->L+6 (26%)
59	35167	284	0.001	H-17->L+1 (23%), H-5->L+2 (12%), HOMO->L+6 (35%)
60	35296	283	0.0013	H-5->L+2 (61%), HOMO->L+6 (10%)

Table S8. Electronic transitions calculated for **RuSPy**IrCp* by means of TD DFT

No.	Energy (cm^{-1})	Wavelength (nm)	Osc. Strength	Major contribs
1	13990	715	0.1641	HOMO->LUMO (78%)
2	14674	681	0.0644	H-2->LUMO (13%), HOMO->L+1 (74%)
3	15855	631	0.0525	H-1->LUMO (85%)
4	17007	588	0.0981	H-2->LUMO (50%), H-1->L+1 (33%)
5	17590	569	0.0441	H-3->LUMO (29%), H-2->LUMO (24%), H-1->L+1 (40%)
6	17893	559	0.048	H-6->LUMO (20%), H-4->LUMO (20%), H-3->LUMO (30%), H-1->L+1 (17%)
7	18607	537	0.0726	H-6->L+1 (11%), H-4->L+1 (17%), H-2->L+1 (56%)
8	18955	528	0.0345	H-4->LUMO (33%), H-4->L+1 (38%)
9	19263	519	0.0165	H-4->L+1 (18%), H-3->L+1 (35%), H-2->L+1 (28%)
10	19724	507	0.0278	H-4->LUMO (32%), H-4->L+1 (16%), H-3->LUMO (10%), H-3->L+1 (27%)
11	19859	504	0.0788	H-5->LUMO (79%)
12	20763	482	0.0712	H-6->LUMO (50%), H-5->L+1 (12%)
13	21470	466	0.0515	H-8->LUMO (30%), H-5->L+1 (54%)
14	21617	463	0.0026	H-8->LUMO (30%), H-7->LUMO (26%), H-6->L+1 (17%)
15	22481	445	0.124	H-8->L+1 (12%), H-7->LUMO (27%), H-6->L+1 (34%), H-3->L+1 (10%)
16	22550	443	0.0737	H-8->LUMO (14%), H-8->L+1 (13%), H-7->LUMO (19%), H-7->L+1 (24%), H-6->L+1 (10%)
17	23759	421	0.4916	H-8->L+1 (43%), H-7->LUMO (17%)
18	23988	417	0.126	H-9->LUMO (27%), H-8->L+1 (12%), H-7->L+1 (36%)
19	24621	406	0.1168	H-9->LUMO (63%)
20	24978	400	0.0655	H-1->L+5 (56%), HOMO->L+5 (23%)
21	25697	389	0.0055	H-10->LUMO (35%), HOMO->L+2 (50%)

22	25989	385	0.0317	H-10->LUMO (24%), H-9->L+1 (25%), HOMO->L+3 (39%)
23	26086	383	0.0217	H-9->L+1 (11%), H-2->L+5 (49%), H-1->L+5 (10%)
24	26262	381	0.0088	H-9->L+1 (54%), HOMO->L+3 (20%)
25	26622	376	0.0573	H-10->LUMO (15%), H-10->L+1 (11%), HOMO->L+2 (25%), HOMO->L+3 (24%)
26	27085	369	0.0381	H-10->L+1 (32%), HOMO->L+4 (59%)
27	27396	365	0.1319	H-10->L+1 (47%), HOMO->L+4 (25%)
28	28076	356	0.0534	H-14->LUMO (53%), H-11->LUMO (17%)
29	28201	355	0.0048	H-13->LUMO (13%), H-12->LUMO (20%), H-11->LUMO (60%)
30	28425	352	0.0089	H-13->LUMO (62%), H-12->LUMO (15%)
31	28480	351	0.0135	H-14->LUMO (18%), H-12->LUMO (57%), H-11->LUMO (17%)
32	28813	347	0.0061	H-15->LUMO (13%), H-1->L+2 (34%)
33	28880	346	0.0095	H-5->L+5 (16%), H-3->L+5 (23%), HOMO->L+5 (30%)
34	29076	344	0.0428	H-15->LUMO (56%)
35	29209	342	0.1407	H-14->L+1 (35%), H-13->L+1 (32%)
36	29347	341	0.0112	H-1->L+2 (42%)
37	29555	338	0.0103	H-16->LUMO (39%), H-14->L+1 (14%), H-12->L+1 (14%)
38	29613	338	0.0518	H-12->L+1 (20%), H-11->L+1 (52%)
39	29757	336	0.0058	H-12->L+1 (54%)
40	29820	335	0.0175	H-18->LUMO (16%), H-17->LUMO (60%)
41	29895	335	0.0067	H-16->LUMO (16%), H-14->L+1 (14%), H-13->L+1 (17%), H-2->L+2 (16%)
42	29931	334	0.014	H-18->LUMO (17%), H-16->LUMO (20%), H-11->L+1 (12%), H-2->L+2 (14%), H-1->L+4 (14%)
43	30034	333	0.0158	H-18->LUMO (30%), H-13->L+1 (18%), H-11->L+1 (14%)
44	30164	332	0.0017	H-3->L+3 (10%), H-2->L+3 (21%), H-1->L+3 (45%)
45	30448	328	0.0145	H-2->L+2 (27%), H-1->L+4 (31%)
46	30606	327	0.0058	H-15->L+1 (18%), H-2->L+3 (13%), HOMO->L+5 (14%), HOMO->L+6 (14%)
47	30646	326	0.013	H-15->L+1 (48%), H-2->L+3 (10%), H-1->L+3 (12%)
48	30678	326	0.0073	HOMO->L+6 (80%)
49	30739	325	0.0002	H-2->L+3 (19%), H-1->L+3 (15%), HOMO->L+5 (16%)
50	30845	324	0.0071	H-16->L+1 (76%)
51	31041	322	0.0119	H-18->L+1 (20%), H-16->L+1 (12%), H-4->L+2 (24%), H-2->L+4 (14%)
52	31069	322	0.0107	H-20->LUMO (19%), H-19->LUMO (44%)
53	31096	322	0.0079	H-18->L+1 (11%), H-3->L+2 (19%), H-2->L+4 (33%)
54	31252	320	0.021	H-18->L+1 (26%), H-4->L+2 (28%), H-3->L+2 (13%)
55	31260	320	0.0018	H-20->LUMO (12%), H-17->L+1 (63%)
56	31570	317	0.0119	H-20->LUMO (40%), H-19->LUMO (36%)
57	31655	316	0.0099	H-3->L+2 (34%), H-2->L+4 (13%)

58	31839	314	0.036	H-4->L+3 (71%)
59	32041	312	0.0156	H-4->L+4 (31%), H-3->L+4 (11%)
60	32306	310	0.0518	H-19->L+1 (15%), H-4->L+4 (14%), HOMO->L+13 (11%)

Table S9. Electronic transitions calculated for **NiMeP**RuCym by means of TD DFT

No.	Energy (cm^{-1})	Wavelength (nm)	Osc. Strength	Major contribs
1	13130	762	0.0056	H-3->L+1 (27%), H-3->L+2 (15%)
2	14011	714	0.0088	H-5->L+1 (24%), H-5->L+2 (13%), H-4->L+1 (12%)
3	14125	708	0.043	HOMO->LUMO (13%), HOMO->L+1 (23%)
4	14624	684	0.0914	HOMO->LUMO (64%)
5	15885	630	0.0205	H-3->LUMO (18%), H-1->LUMO (44%), HOMO->L+1 (15%)
6	16216	617	0.012	H-2->LUMO (13%), H-1->LUMO (17%), HOMO->L+1 (23%)
7	16727	598	0.061	H-3->LUMO (41%), H-1->LUMO (21%)
8	17418	574	0.0321	H-2->LUMO (29%), H-1->L+1 (60%)
9	18263	548	0.1109	H-4->LUMO (28%), H-2->LUMO (25%), H-1->L+1 (21%)
				H-7->LUMO (16%), H-6->LUMO (13%), H-5->LUMO (10%),
10	19056	525	0.0257	H-4->LUMO (15%), H-2->L+1 (26%)
11	19285	519	0.0221	H-5->LUMO (62%)
12	19491	513	0.2189	H-6->LUMO (16%), H-4->LUMO (18%), H-2->L+1 (32%)
13	19919	502	0.0882	H-7->LUMO (51%)
14	20286	493	0.015	H-2->L+3 (26%), HOMO->L+3 (19%)
15	20322	492	0.0041	H-3->L+1 (18%)
16	20509	488	0.0051	H-2->L+3 (14%), H-1->L+3 (29%)
17	20610	485	0.0232	H-3->L+1 (23%), H-1->L+3 (11%)
18	21216	471	0.099	H-6->L+1 (12%), H-4->L+1 (46%)
19	21281	470	0.008	H-5->L+1 (57%), H-5->L+2 (13%)
20	22220	450	0.2515	H-6->LUMO (38%)
21	23146	432	0.4661	H-6->L+1 (46%)
22	23562	424	0.156	H-7->L+1 (57%)
23	23794	420	0.0028	H-5->L+3 (16%), H-4->L+3 (22%), H-3->L+3 (21%)
24	24529	408	0.1225	H-8->LUMO (83%)
25	25671	390	0.0494	H-8->L+1 (49%), HOMO->L+2 (24%)
26	25996	385	0.159	H-9->LUMO (15%), H-8->L+1 (35%), HOMO->L+2 (31%)
27	26247	381	0.0326	H-9->LUMO (69%), H-8->L+1 (13%)
28	27020	370	0.0123	H-9->L+1 (47%), HOMO->L+4 (28%)
29	27397	365	0.1067	H-9->L+1 (36%), HOMO->L+3 (12%), HOMO->L+4 (23%)
30	27445	364	0.0145	H-1->L+3 (11%), HOMO->L+3 (40%), HOMO->L+4 (18%)
31	27932	358	0.0306	H-10->LUMO (61%)
32	28359	353	0.0139	H-1->L+2 (48%), H-1->L+3 (26%)
33	28538	350	0.0699	H-2->L+2 (48%), H-2->L+3 (16%)
34	28843	347	0.0667	H-16->LUMO (11%), H-12->LUMO (65%)
35	28984	345	0.0101	H-12->L+1 (14%), H-11->LUMO (13%), H-10->L+1 (53%)
36	29107	344	0.0251	H-11->LUMO (77%)
37	29456	339	0.0058	H-19->LUMO (19%), H-1->L+4 (33%)
38	29497	339	0.0572	H-13->LUMO (58%), H-10->LUMO (11%)

39	29622	338	0.0042	H-1->L+5 (17%), HOMO->L+5 (28%)
40	29660	337	0.058	H-12->L+1 (41%), H-10->L+1 (19%), H-1->L+4 (12%)
41	29721	336	0.0299	H-19->LUMO (10%), H-1->L+4 (31%)
42	29943	334	0.043	H-11->L+1 (90%)
43	30326	330	0.0096	H-16->LUMO (12%), H-14->LUMO (62%)
44	30389	329	0.004	H-1->L+8 (11%)
45	30471	328	0.016	H-15->LUMO (14%), H-3->L+4 (16%), H-2->L+4 (18%)
46	30504	328	0.0063	H-17->LUMO (29%), H-15->LUMO (18%), H-2->L+4 (15%)
47	30569	327	0.0025	H-17->LUMO (11%), H-15->LUMO (31%), H-14->LUMO (15%), H-13->LUMO (17%)
48	30801	325	0.0549	H-16->LUMO (18%), H-13->L+1 (29%)
49	30888	324	0.0055	H-16->LUMO (18%), H-3->L+4 (18%), H-2->L+4 (13%)
50	30943	323	0.0333	H-18->LUMO (16%), H-13->L+1 (26%)
51	31126	321	0.0071	H-16->L+1 (10%), H-14->L+1 (38%)
52	31204	320	0.0074	H-14->L+1 (21%), H-4->L+2 (28%)
53	31336	319	0.0226	H-16->L+1 (15%), H-14->L+1 (21%)
54	31508	317	0.0068	H-15->L+1 (60%)
55	31634	316	0.0124	H-16->L+1 (31%), H-5->L+4 (10%), H-4->L+4 (12%)
56	31777	315	0.0366	H-18->LUMO (14%)
57	31893	314	0.0168	H-4->L+3 (10%), H-3->L+3 (16%)
58	31930	313	0.0009	H-4->L+3 (13%), H-4->L+5 (11%)
59	32041	312	0.0035	H-19->L+1 (13%), H-18->L+1 (15%), H-17->L+1 (33%)
60	32102	312	0.0052	H-18->L+1 (13%), H-17->L+1 (11%), H-6->L+2 (10%)

Table S10. Electronic transitions calculated for **RuSPy**RhCp* by means of TD DFT

No.	Energy (cm^{-1})	Wavelength (nm)	Osc. Strength	Major contribs
1	14437	693	0.1802	HOMO->LUMO (72%), HOMO->L+1 (15%)
2	15012	666	0.0912	H-2->LUMO (13%), HOMO->LUMO (16%), HOMO->L+1 (66%)
3	16433	609	0.0231	H-1->LUMO (91%)
4	17722	564	0.0616	H-6->LUMO (13%), H-4->LUMO (30%), H-2->LUMO (20%), H-1->L+1 (13%)
5	18110	552	0.0284	H-1->L+1 (64%)
6	18752	533	0.0715	H-3->LUMO (52%), H-2->LUMO (22%)
7	18948	528	0.0442	H-4->LUMO (14%), H-4->L+1 (51%), H-2->LUMO (13%), H-1->L+1 (12%)
8	18973	527	0.0604	H-6->L+1 (12%), H-4->LUMO (20%), H-4->L+1 (14%), H-2->LUMO (16%), H-2->L+1 (17%)
9	19729	507	0.0341	H-4->LUMO (17%), H-4->L+1 (12%), H-3->LUMO (23%), H-3->L+1 (19%), H-2->L+1 (13%)
10	20361	491	0.0066	H-5->LUMO (19%), H-3->L+1 (28%), H-2->L+1 (18%), H-1->L+2 (18%)
11	20417	490	0.0617	H-5->LUMO (32%), H-3->L+1 (16%), H-2->L+1 (25%), H-1->L+2 (11%)
12	20608	485	0.0233	H-5->LUMO (24%), H-1->L+2 (54%)

13	21269	470	0.0535	H-7->LUMO (11%), H-6->LUMO (51%), H-5->L+1 (15%)
14	21760	460	0.0666	H-8->LUMO (19%), H-7->LUMO (39%), H-6->LUMO (11%), H-5->LUMO (12%)
15	22107	452	0.0636	H-7->LUMO (14%), H-5->L+1 (41%)
16	22155	451	0.0278	H-5->L+1 (12%), H-3->L+2 (20%), H-2->L+2 (25%), HOMO->L+2 (16%)
17	22647	442	0.0288	H-8->LUMO (36%), H-7->L+1 (34%)
18	23328	429	0.2076	H-7->LUMO (12%), H-6->L+1 (54%)
19	23843	419	0.4795	H-8->LUMO (14%), H-8->L+1 (13%), H-7->L+1 (33%)
20	24134	414	0.0124	H-9->LUMO (52%), H-8->L+1 (35%)
21	24652	406	0.2028	H-9->LUMO (39%), H-8->L+1 (28%), H-7->L+1 (11%)
22	24940	401	0.0098	H-3->L+2 (11%), HOMO->L+2 (59%)
23	25907	386	0.023	H-10->LUMO (52%), HOMO->L+3 (33%)
24	25994	385	0.0146	H-9->L+1 (77%), HOMO->L+3 (11%)
25	26221	381	0.0311	H-10->LUMO (25%), HOMO->L+4 (42%)
26	26378	379	0.0219	H-6->L+2 (14%), H-5->L+2 (14%), HOMO->L+2 (11%)
27	26762	374	0.0455	HOMO->L+3 (27%), HOMO->L+4 (39%)
28	27233	367	0.0328	H-11->LUMO (12%), H-10->L+1 (37%), HOMO->L+5 (40%)
29	27268	367	0.0168	H-11->LUMO (61%), H-10->L+1 (28%)
30	27571	363	0.078	H-11->LUMO (11%), H-10->L+1 (19%), HOMO->L+5 (40%)
31	27987	357	0.058	H-15->LUMO (13%), H-14->LUMO (38%), H-13->LUMO (13%), H-12->LUMO (18%)
32	28334	353	0.0209	H-14->LUMO (12%), H-13->LUMO (39%), H-1->L+7 (10%)
33	28437	352	0.0131	H-14->LUMO (20%), H-12->LUMO (71%)
34	28500	351	0.0176	H-13->LUMO (25%), H-1->L+5 (10%), H-1->L+7 (18%)
35	28762	348	0.0312	H-15->LUMO (49%), H-11->L+1 (17%)
36	28942	346	0.0169	H-15->LUMO (11%), H-11->L+1 (76%)
37	29142	343	0.0165	H-4->L+5 (14%), H-2->L+5 (13%)
38	29175	343	0.1647	H-14->L+1 (55%), H-13->L+1 (16%)
39	29506	339	0.0145	H-17->LUMO (10%), H-16->LUMO (36%), H-14->L+1 (13%), H-13->L+1 (20%)
40	29618	338	0.0429	H-12->L+1 (85%)
41	29762	336	0.0126	H-18->LUMO (10%), H-17->LUMO (75%)
42	29780	336	0.006	H-16->LUMO (20%), H-13->L+1 (47%)
43	29905	334	0.0178	H-18->LUMO (49%), H-16->LUMO (29%)
44	29921	334	0.0068	H-1->L+3 (60%)
45	30078	332	0.0023	H-5->L+2 (21%), H-3->L+2 (24%), H-2->L+2 (23%)
46	30292	330	0.0217	H-15->L+1 (67%)
47	30526	328	0.0046	H-3->L+7 (11%), H-1->L+5 (13%)
48	30543	327	0.0025	H-2->L+4 (34%), H-1->L+4 (24%)
49	30792	325	0.0065	H-16->L+1 (80%)
50	30884	324	0.0117	HOMO->L+6 (47%)
51	30935	323	0.002	H-2->L+3 (18%), HOMO->L+6 (44%)
52	30999	323	0.0162	H-20->LUMO (11%), H-19->LUMO (23%), H-18->L+1 (27%)
53	31023	322	0.0096	H-20->LUMO (15%), H-19->LUMO (14%), H-18->L+1 (15%), H-17->L+1 (14%), H-14->L+1 (10%), H-4->L+3 (14%)
54	31106	321	0.0039	H-9->L+2 (46%), H-2->L+2 (17%)

55	31115	321	0.0101	H-17->L+1 (10%), H-2->L+3 (15%)
56	31223	320	0.0072	H-17->L+1 (53%), H-4->L+3 (13%)
57	31267	320	0.0115	H-18->L+1 (17%), H-4->L+3 (26%)
58	31472	318	0.0023	H-20->LUMO (34%), H-19->LUMO (25%), H-4->L+2 (17%)
59	31479	318	0.0163	H-4->L+2 (55%)
60	31641	316	0.0109	H-2->L+4 (28%), H-1->L+4 (39%)

Table S11. Electronic transitions calculated for **NiMeP**IrCp* by means of TD DFT

No.	Energy (cm^{-1})	Wavelength (nm)	Osc. Strength	Major contribs
1	12997	769	0.0042	H-3->L+1 (36%), H-3->L+2 (27%)
2	13885	720	0.0022	H-5->L+1 (23%), H-5->L+2 (18%), H-4->L+1 (19%), H-4->L+2 (14%)
3	14048	712	0.0365	H-6->L+1 (11%), HOMO->L+1 (26%)
4	14747	678	0.1104	HOMO->LUMO (71%)
5	16005	625	0.0151	H-3->LUMO (22%), H-1->LUMO (42%), HOMO->L+1 (16%)
6	16342	612	0.0145	H-2->LUMO (17%), H-1->LUMO (19%), HOMO->L+1 (22%)
7	16759	597	0.0567	H-3->LUMO (50%), H-1->LUMO (26%)
8	17615	568	0.0434	H-2->LUMO (28%), H-1->L+1 (62%)
9	18288	547	0.0954	H-4->LUMO (31%), H-2->LUMO (27%), H-1->L+1 (25%)
10	19256	519	0.0153	H-7->LUMO (15%), H-6->LUMO (23%), H-4->LUMO (16%), H-2->L+1 (29%)
11	19370	516	0.0182	H-5->LUMO (52%)
12	19769	506	0.1646	H-7->LUMO (10%), H-6->LUMO (24%), H-2->L+1 (38%)
13	20236	494	0.0597	H-22->L+2 (13%), H-19->L+2 (10%)
14	20386	491	0.1666	H-7->LUMO (16%), H-5->LUMO (26%), H-4->LUMO (10%)
15	20679	484	0.0319	H-3->L+1 (38%), H-3->L+2 (22%)
16	21321	469	0.0796	H-5->L+1 (16%), H-5->L+2 (15%), H-4->L+1 (30%), H-4->L+2 (12%)
17	21907	456	0.088	H-6->L+1 (17%), H-5->L+1 (44%), H-4->L+1 (16%)
18	22193	451	0.1957	H-7->LUMO (16%), H-6->LUMO (25%), H-6->L+1 (15%)
19	23186	431	0.4222	H-7->LUMO (14%), H-6->L+1 (39%)
20	23749	421	0.1277	H-7->L+1 (65%)
21	24987	400	0.073	H-8->LUMO (71%)
22	25089	399	0.0882	H-8->LUMO (15%), H-1->L+4 (48%), HOMO->L+4 (18%)
23	25749	388	0.1127	H-8->L+1 (15%), HOMO->L+2 (51%)
24	26118	383	0.0403	H-9->LUMO (80%)
25	26312	380	0.0313	H-2->L+4 (52%)
26	26518	377	0.0962	H-8->L+1 (72%)
27	27166	368	0.0046	H-9->L+1 (41%), HOMO->L+3 (35%)
28	27518	363	0.0826	H-9->L+1 (50%), HOMO->L+3 (30%)
29	27943	358	0.0426	H-10->LUMO (56%)
30	28366	353	0.0014	H-1->L+2 (76%)
31	28596	350	0.0888	H-12->LUMO (15%), H-2->L+2 (50%)
32	28629	349	0.0021	H-12->LUMO (58%)
33	28779	347	0.0679	H-17->LUMO (10%), H-13->LUMO (59%)
34	28893	346	0.0201	H-11->LUMO (76%)

35	29047	344	0.0033	H-10->L+1 (41%)
36	29171	343	0.0138	H-5->L+4 (19%), H-4->L+4 (18%), HOMO->L+4 (21%)
37	29360	341	0.0117	H-14->LUMO (49%), H-10->L+1 (10%)
38	29609	338	0.08	H-13->L+1 (40%), H-12->L+1 (10%), H-10->L+1 (22%)
39	29704	337	0.0275	H-19->LUMO (24%), H-16->LUMO (14%), H-13->LUMO (13%)
40	29810	335	0.007	H-11->L+1 (52%), H-1->L+3 (30%)
41	29880	335	0.0369	H-11->L+1 (39%), H-1->L+3 (42%)
42	30141	332	0.0015	H-17->LUMO (14%), H-15->LUMO (61%)
43	30281	330	0.0531	H-16->LUMO (14%), H-13->L+1 (10%), H-12->L+1 (48%)
44	30378	329	0.0046	H-16->LUMO (40%), H-15->LUMO (11%), H-14->LUMO (18%)
45	30665	326	0.0024	H-17->LUMO (11%), H-3->L+3 (37%), H-2->L+3 (14%)
46	30707	326	0.0157	H-19->LUMO (11%), H-17->LUMO (28%), H-3->L+3 (13%)
47	30852	324	0.0423	H-14->L+1 (54%), H-12->L+1 (10%)
48	30992	323	0.0071	H-15->L+1 (30%), H-3->L+3 (10%), H-2->L+3 (25%)
49	31073	322	0.0121	H-15->L+1 (34%), H-3->L+3 (10%), H-2->L+3 (19%)
50	31157	321	0.0062	H-5->L+2 (18%), H-4->L+2 (33%), HOMO->L+4 (12%)
51	31250	320	0.0063	H-17->L+1 (17%), H-13->L+1 (11%)
52	31364	319	0.0016	H-17->L+1 (10%), HOMO->L+4 (19%)
53	31419	318	0.0029	H-17->L+1 (10%), H-16->L+1 (54%)
54	31616	316	0.0036	H-18->LUMO (24%), H-16->L+1 (15%)
55	31741	315	0.0193	H-20->LUMO (20%), H-19->LUMO (11%), H-18->LUMO (21%), H-17->L+1 (17%)
56	31771	315	0.046	H-20->LUMO (27%), H-5->L+3 (11%), H-4->L+3 (26%)
57	32090	312	0.0021	H-19->L+1 (37%), H-18->L+1 (40%)
58	32563	307	0.0017	H-21->LUMO (22%), H-21->L+1 (10%), H-20->L+1 (19%)
59	32704	306	0.0086	H-21->L+1 (11%), H-20->L+1 (14%), H-19->L+1 (29%), H-18->L+1 (20%)
60	32883	304	0.0267	H-22->LUMO (32%), H-18->L+1 (14%)

Table S12. Electronic transitions calculated for **ClNCP**IrCp* by means of TD DFT

No.	Energy (cm^{-1})	Wavelength (nm)	Osc. Strength	Major contribs
1	12298	813	0.0271	H-1->LUMO (14%), HOMO->LUMO (83%)
2	14347	697	0.0944	H-1->LUMO (69%), HOMO->LUMO (10%)
3	14889	672	0.0102	H-2->LUMO (82%), HOMO->L+1 (12%)
4	15767	634	0.1108	H-2->LUMO (11%), H-1->L+1 (14%), HOMO->L+1 (62%)
5	17204	581	0.0834	H-4->LUMO (21%), H-1->L+1 (70%)
6	17743	564	0.0219	H-3->LUMO (84%), H-2->L+1 (13%)
7	18085	553	0.0735	H-3->LUMO (10%), H-2->L+1 (74%)
8	20751	482	0.0059	H-5->LUMO (85%)
9	20791	481	0.0133	H-3->L+1 (95%)
10	22402	446	0.0954	H-6->LUMO (69%), H-4->LUMO (12%)
11	22746	440	0.3407	H-7->LUMO (22%), H-4->L+1 (42%)
12	23586	424	0.6873	H-7->LUMO (14%), H-6->LUMO (18%), H-5->L+1 (15%), H-4->LUMO (24%)

13	24051	416	0.2828	H-7->LUMO (11%), H-5->L+1 (56%)
14	24386	410	0.1126	H-7->LUMO (27%), H-5->L+1 (17%), HOMO->L+2 (22%)
15	24859	402	0.0674	H-9->LUMO (12%), H-8->LUMO (42%), HOMO->L+3 (11%)
16	24960	401	0.0324	H-8->LUMO (40%), H-7->LUMO (11%), H-1->L+3 (11%), HOMO->L+3 (15%)
17	25211	397	0.0149	H-9->LUMO (13%), H-6->L+1 (15%), H-1->L+3 (10%), HOMO->L+2 (34%), HOMO->L+3 (13%)
18	25661	390	0.1571	H-9->LUMO (33%), H-6->L+1 (49%)
19	26088	383	0.1483	H-9->LUMO (13%), H-6->L+1 (12%), H-1->L+2 (32%), HOMO->L+2 (12%)
20	26181	382	0.0114	H-2->L+3 (63%), H-2->L+4 (10%)
21	26942	371	0.1186	H-10->LUMO (18%), H-1->L+2 (43%)
22	27203	368	0.1594	H-7->L+1 (76%)
23	27537	363	0.062	H-11->LUMO (19%), H-10->LUMO (63%)
24	27830	359	0.1039	H-11->LUMO (43%), H-2->L+2 (22%)
25	27868	359	0.1033	H-11->LUMO (26%), H-2->L+2 (59%)
26	27969	358	0.0032	H-8->L+1 (92%)
27	28827	347	0.0307	H-9->L+1 (91%)
28	29692	337	0.029	H-13->LUMO (20%), H-12->LUMO (57%), H-10->L+1 (13%)
29	29834	335	0.0009	H-12->LUMO (10%), H-10->L+1 (79%)
30	30019	333	0.0107	H-3->L+2 (16%), H-3->L+3 (51%)
31	30226	331	0.0113	H-11->L+1 (88%)
32	30325	330	0.0628	H-13->LUMO (58%), H-12->LUMO (14%)
33	30893	324	0.0153	H-3->L+2 (77%)
34	31185	321	0.0396	H-21->LUMO (27%), H-20->LUMO (16%), H-19->LUMO (28%)
35	31387	319	0.0048	H-14->LUMO (30%), HOMO->L+5 (15%)
36	31542	317	0.0047	H-14->LUMO (16%), HOMO->L+5 (36%)
37	31584	317	0.0156	HOMO->L+4 (52%), HOMO->L+5 (16%)
38	31713	315	0.0188	H-4->L+2 (23%), H-1->L+3 (11%), HOMO->L+6 (21%)
39	31789	315	0.0016	H-16->LUMO (10%), H-14->LUMO (10%), H-4->L+2 (21%), H-1->L+3 (13%), HOMO->L+3 (14%)
40	31890	314	0.003	H-15->LUMO (27%), HOMO->L+7 (34%)
41	31909	313	0.0048	H-1->L+3 (15%), HOMO->L+6 (40%), HOMO->L+7 (14%)
42	31953	313	0.0004	H-16->LUMO (11%), H-15->LUMO (38%), HOMO->L+7 (11%)
43	32087	312	0.0007	H-16->LUMO (15%), HOMO->L+8 (51%)
44	32255	310	0.0163	H-16->LUMO (12%), H-14->LUMO (12%), H-12->L+1 (26%), H-4->L+2 (22%), HOMO->L+6 (10%)
45	32292	310	0.0074	H-16->LUMO (25%), H-12->L+1 (20%), HOMO->L+8 (17%), HOMO->L+9 (18%)
46	32403	309	0.0049	H-17->LUMO (38%), HOMO->L+9 (18%)
47	32649	306	0.0013	H-17->LUMO (36%), HOMO->L+9 (26%)
48	32906	304	0.0039	H-1->L+11 (19%), HOMO->L+11 (38%)
49	32977	303	0.0812	H-18->LUMO (47%), HOMO->L+9 (13%)
50	33155	302	0.0016	H-18->LUMO (10%), H-13->L+1 (71%)
51	33301	300	0.0009	H-1->L+5 (10%), H-1->L+6 (13%), H-1->L+7 (54%)
52	33456	299	0.0052	H-21->LUMO (20%), H-19->LUMO (32%)
53	33645	297	0.0241	H-19->LUMO (10%), H-1->L+4 (24%), H-1->L+9 (19%)

54	33794	296	0.0065	H-5->L+2 (82%)
55	34017	294	0.0069	H-20->LUMO (14%), H-19->L+1 (30%), H-14->L+1 (34%)
56	34069	294	0.0392	H-21->LUMO (21%), H-20->LUMO (22%), H-1->L+5 (16%)
57	34114	293	0.0193	H-21->LUMO (10%), H-20->LUMO (11%), H-1->L+5 (40%), H-1->L+7 (13%)
58	34168	293	0.0097	H-18->L+1 (13%), H-17->L+1 (25%), H-14->L+1 (20%)
59	34254	292	0.0004	H-1->L+4 (17%), H-1->L+6 (48%), H-1->L+7 (10%)
60	34379	291	0.0376	H-15->L+1 (72%)

References

[S1] Gaussian 16, Revision C.01, Frisch, M. J.; Trucks, G. W.; Schlegel, H. B.; Scuseria, G. E.; Robb, M. A.; Cheeseman, J. R.; Scalmani, G.; Barone, V.; Petersson, G. A.; Nakatsuji, H.; Li, X.; Caricato, M.; Marenich, A. V.; Bloino, J.; Janesko, B. G.; Gomperts, R.; Mennucci, B.; Hratchian, H. P.; Ortiz, J. V.; Izmaylov, A. F.; Sonnenberg, J. L.; Williams-Young, D.; Ding, F.; Lipparini, F.; Egidi, F.; Goings, J.; Peng, B.; Petrone, A.; Henderson, T.; Ranasinghe, D.; Zakrzewski, V. G.; Gao, J.; Rega, N.; Zheng, G.; Liang, W.; Hada, M.; Ehara, M.; Toyota, K.; Fukuda, R.; Hasegawa, J.; Ishida, M.; Nakajima, T.; Honda, Y.; Kitao, O.; Nakai, H.; Vreven, T.; Throssell, K.; Montgomery, J. A., Jr.; Peralta, J. E.; Ogliaro, F.; Bearpark, M. J.; Heyd, J. J.; Brothers, E. N.; Kudin, K. N.; Staroverov, V. N.; Keith, T. A.; Kobayashi, R.; Normand, J.; Raghavachari, K.; Rendell, A. P.; Burant, J. C.; Iyengar, S. S.; Tomasi, J.; Cossi, M.; Millam, J. M.; Klene, M.; Adamo, C.; Cammi, R.; Ochterski, J. W.; Martin, R. L.; Morokuma, K.; Farkas, O.; Foresman, J. B.; Fox, D. J. Gaussian, Inc., Wallingford CT, 2016.
[S2] A. D. Becke, *J. Chem. Phys.*, **1993**, *98*, 5648.
[S3] C. Lee, W. Yang, and R. G. Parr, *Phys. Rev. B*, **1988**, *37*, 785.
[S4] F. Weigend and R. Ahlrichs, *Phys. Chem. Chem. Phys.*, **2005**, *7*, 3297.
[S5] N. M. O'Boyle, A. L. Tenderholt, K. M. Langner, *J. Comp. Chem.* **2008,** *29* 839.
